# Novel Structural and Functional Motifs in *cellulose synthase* (*CesA*) Genes of Bread Wheat (*Triticum aestivum*, L.)

**DOI:** 10.1371/journal.pone.0147046

**Published:** 2016-01-15

**Authors:** Simerjeet Kaur, Kanwarpal S. Dhugga, Kulvinder Gill, Jaswinder Singh

**Affiliations:** 1 Department of Plant Science, McGill University, Sainte Anne de Bellevue, QC, Canada; 2 Genetic Discovery, DuPont Pioneer, 7300 NW 62nd Avenue, Johnston, IA, United States of America; 3 Department of Crop and Soil Science, Washington State University, Pullman, WA, United States of America; University of California Davis, UNITED STATES

## Abstract

Cellulose is the primary determinant of mechanical strength in plant tissues. Late-season lodging is inversely related to the amount of cellulose in a unit length of the stem. Wheat is the most widely grown of all the crops globally, yet information on its *CesA* gene family is limited. We have identified 22 *CesA* genes from bread wheat, which include homoeologs from each of the three genomes, and named them as *TaCesAXA*, *TaCesAXB* or *TaCesAXD*, where X denotes the gene number and the last suffix stands for the respective genome. Sequence analyses of the CESA proteins from wheat and their orthologs from barley, maize, rice, and several dicot species (Arabidopsis, beet, cotton, poplar, potato, rose gum and soybean) revealed motifs unique to monocots (Poales) or dicots. Novel structural motifs CQIC and SVICEXWFA were identified, which distinguished the CESAs involved in the formation of primary and secondary cell wall (PCW and SCW) in all the species. We also identified several new motifs specific to monocots or dicots. The conserved motifs identified in this study possibly play functional roles specific to PCW or SCW formation. The new insights from this study advance our knowledge about the structure, function and evolution of the *CesA* family in plants in general and wheat in particular. This information will be useful in improving culm strength to reduce lodging or alter wall composition to improve biofuel production.

## Introduction

Cellulose is the primary determinant of mechanical strength in plants [1, 2]. It is also the world's most abundant renewable carbon source [3, 4]. In plants, the secondary cell wall is deposited inside the primary wall and, because of its greater thickness, it generally constitutes majority of the vegetative biomass [5]. Primary cell wall is deposited during cell division and expansion stages, whereas the secondary cell wall begins to form as cell expansion approaches cessation [6]. Cellulose in plants is synthesized by multimeric protein complexes, which consist of hexameric, rosette-like structures in the plasma membrane [7]. Individual members of each of the hexameric components are referred to as *cellulose synthase A* (*CesA*), where the letter *A* stands for the catalytic subunit [3]. Arabidopsis (*Arabidopsis thaliana*) genome contains at least 10 *CesA* genes, which cluster into six groups [8–10]. Mutational genetics established that six of the ten genes each had a nonredundant function in primary or secondary cell wall (PCW or SCW) formation. Three of the genes, *AtCesA1*, *AtCesA3*, and *AtCesA6*, are involved in PCW formation and another three, *AtCesA4*, *AtCesA7* and *AtCesA8* in SCW formation [11, 12]. The remaining genes, *AtCesA2*, *5* and *9*, are partially redundant with *AtCesA6* [13, 14]. *AtCesA10* remains uncharacterized. Maize and rice possess 13 and 11 *CesA* genes, respectively [15, 16]. Barley has nine genes, of which *HvCesA1*, *HvCesA2*, *and HvCesA6* make PCW, and *HvCesA4*, *HvCesA7*, and *HvCesA8* form SCW [17]. *HvCesA3*, *5* and *9* are different from both the groups because of their unique tissue-specific transcript levels [18]. Mapping studies from Arabidopsis, maize and rice revealed that the members of the *CesA* gene family were spread across the genome although some genes were clustered together [16, 19].

Plant CESAs belong to family 2 of glycosyl transferases (GT2), which catalyze beta linkage between the glycosyl residues. CESAs are intrinsic plasma membrane proteins with their catalytic domains extending into the cytoplasm [20]. Each of the six subunits of a cellulose synthase complex (CSC) is believed to be composed of 6 enzymatically active CESAproteins. Each of the CESA proteins catalyzes the synthesis of an individual beta-1,4-linked chain [21, 22]. Multiple chains extruded from the CSC then polymerize through hydrogen bond formation into a microfibril outside the plasma membrane.

A CESA protein of higher plants possesses eight transmembrane domains (TMDs), which are believed to form a pore in the plasma membrane to allow extrusion of the newly synthesised glucan chain. Two zinc-finger domains (ZnF), which are highly homologous to the RING-finger motif, are present on the cytoplasmic face close to the amino terminus [23]. The central or the catalytic domain, is located between the second and third TMDs [24]. Three aspartyl residues (referred to as D1, D2, and D3) and a QXXRW motif in the catalytic domain of the CESA proteins are conserved across all the species studied thus far. The D1 and D2 residues are believed to coordinate UDP binding while D3 provides a catalytic base for glucan chain extension [25] The QXXRW motif acts as a binding site for the terminal disaccharide of the glucan [21].

Motifs are the conserved groups of residues in proteins, which can be associated with structural and functional variability across species. A highly conserved motif, CXXC, which is located within the ZnF, distnguishes CESAs from the CSL (cellulose synthase-like) proteins [9, 10]. Crystal structure of the cellulose synthase subunit A (BcsA) and accessory protein BcsB of *Rhodobacter sphaeroides* demonstrated the involvement of a single catalytic site in the formation of beta-1,4-glycosidic bond of the glucan chain [21]. Computationally predicted model of GhCESA1 revealed two class-specific regions (C-SR-I and C-SR-II), which distinguished different CESAs, and two plant-conserved regions (P-CR), which were absent in the bacterial BcsA but highly conserved in all the plant CESAs [22, 26–28]. The P-CR might be potentially involved in the multimerization of the plant CESA polypeptides, leading to the formation of rosettes. C-SRs are probably responsible for regulating cellulose synthesis at different developmental stages.

The *CesA* gene family has not yet been compiled from wheat, the most widely grown crop in global agriculture. Functional classification of the *CesA* genes in cereal crops has proved helpful in associating various genes with culm or stalk strength [2, 17]. In this report, we present the *CesA* gene family from wheat. To understand the involvement of the different *CesAs* in primary or secondary wall formation in grasses or dicot plants, we have identified unique sequence motifs. Sequence comparisons of the PCW and SCW *TaCesA* genes were performed at both the DNA and protein levels. Phases of intron evolution were predicted and compared between the groups of the *TaCesA* genes involved in the formation of PCW or SCW. Unique motifs were identified among the representative monocot and dicot species. RNA-seq expression profiling of the *TaCesA* genes revealed unique, homoeolog-specific expression patterns in different tissues.

## Methods and Materials

### Identification of *CesA*s in wheat and their true orthologs from different species

**Fig 1:**
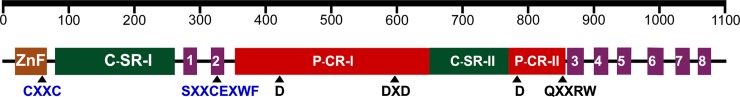
Predicted protein features of wheat cellulose synthase genes. The numbers 1 to 8 in the purple rectangles refers to the transmembrane domains (TMDs). Black triangles localize the conserved motifs. The newly identified motifs CXXC and SXXCEXWF are highlighted in blue and previously reported motifs in black.

The conserved cellulose synthase domains from barley CESA proteins was used as query to perform tBLASTn search with Chromosome Survey Sequence (CSS) (http://plants.ensembl.org/Triticum_aestivum/Info/Index) generated by the International Wheat Genome Sequencing Consortium (IWGSC) [29]. Availability of whole genome sequence of barley (http://webblast.ipk-gatersleben.de/barley/) made it possible to isolate full-length barley *CesA* sequences [18]. Genome databases of *Triticum urartu* and *Aegilops tauschii*, A and D genome progenitors of wheat, respectively, were also explored to identify full-length *CesA* genes for the sequences missing in hexaploid wheat. The homoeologs were first identified from Ensembl Plant database followed by amino acid sequence alignment for the presence of conserved motifs and domains. Highly variable class-specific regions (C-SRs) present in different *CesAs* were used to differentiate the homoeologous genes from each other (Fig 1).

Orthologs of various *CesA* genes were identified through alignment of the wheat *CesA*s with those from Arabidopsis, barley, rice and maize. The ortholog of each gene was selected based on the sequence identity and query coverage, presence of all domains and motifs similar to the query sequence, Amino acid content/size and distance among various new motifs identified in this study relative to the query sequence. Arabidopsis, rice and maize *CesA* sequences were retrieved from Phytozome v9.1: Home (http://www.phytozome.net/) [30].

### Gene structure analysis

Although in this study we identified 22 *TaCesA* genes, comparative studies for gene structure were performed only for the genes that were specific for PCW and SCW cellulose synthesis. Based on analysis of the orthologs, *TaCesA4*, *7* and *8* were characterized as one-to-one orthologs of SCW-specific, *TaCesA1*, *2*, and *6* as PCW-specific, and *TaCesA3*, *5* and *9* as partially redundant to the PCW *CesAs*. The homoeologous copies of each gene shared 95–99% sequence identity in addition to all the motifs and domains. Therefore only one copy among the three homoeologs was used for comparative analysis. Intron-exon boundaries and translation start and stop sites were predicted through alignments of full-length genomic copies of *TaCesA* genes with their corresponding cDNA sequences. The introns and exons were drawn to scale for all the genes as indicated by the cDNA-genomic sequence comparisons. Phases of intron evolution were predicted using Plant Intron Exon Comparison and Evolution database (PIECE) (http://wheat.pw.usda.gov/piece/) [31].

### Protein structure and motif identification

Amino acid sequence similarity of TaCESA protein sequences was determined by multiple sequence alignment (http://www.genome.jp/tools/clustalw/). Color Align Conservation tool (http://www.bioinformatics.org/sms2/color_align_cons.html) was used to differentiate the conserved patterns of aligned sequences. Conserved domains and motifs were identified by manual search in the aligned sequences.

### Phylogenetic analysis

22 newly identified wheat CESA proteins were used to deduce their phylogenetic relationships. Protein sequences for *Arabidopsis thaliana* (AtCESA), *Beta vulgaris* (BvCESA), *Eucalyptus grandis* (EgCESA), *Glycine max* (GmCESA), *Gossypium hirsutum* (GhCESA), *Hordeum vulgare* (HvCESA), *Oryza sativa* (OsCESA), *Populus trichocarpa* (PtCESA), *Solanum tuberosum* (StCESA), *Zea mays* (ZmCESA) were retrieved from NCBI (http://www.ncbi.nlm.nih.gov) [32]. An unrooted phylogenetic tree was constructed with bootstrap analysis over 1000 replicates, using the Neighbor-Joining method using the MEGA6 program [33, 34]. Evolutionary distances were computed using Poisson correction method [35]. All positions containing gaps and missing data were eliminated.

GenBank accession numbers for CESA amino acid sequences used to generate the phylogenetic tree are: AtCESA1, AF027172; AtCESA2, AF027173; AtCESA3, AF027174; AtCESA4, AB006703; AtCESA5, AB016893; AtCESA6, AF062485; AtCESA7, AF088917; AtCESA8, AL035526; AtCESA9, AC007019; AtCESA10, At2G25540; ZmCESA1, AF200525; ZmCESA2, AF200526; ZmCESA3, NP_001292792.1; ZmCESA4, AF200528; ZmCESA5, AF200529; ZmCESA6, AF200530; ZmCESA7, AF200531; ZmCESA8, AF200532; ZmCESA9, AF200533; ZmCESA10, AY372244; ZmCESA11, AY372245; ZmCESA12, AY372246; ZmCESA13, KJ874174; OsCESA1, AF030052; OsCESA2, D48636, OsCESA3, BAD30574; OsCESA4, AK100475; OsCESA5, BAD30574; OsCESA6, XM_477282; OsCESA7, XM_477282; OsCESA8, XM_477093; OsCESA9, XM_477093; OsCESA10, LOC_O*-4*2g29300; OsCESA11, LOC_OS06g39970; HvCESA1, AY483150; HvCESA2, AY483152; HvCESA3, AY483151; HvCESA4, AY483154; HvCESA5/7, AY483153; HvCESA6, AY483155; HvCESA8, AY483156; HvCESA9, AK367031; PtCESA6, XP_002319002; EgCESA5, XP_010063196; StCESA3, XP_006354075; GmCESA2, XP_003531396; GhCESA5, AFB18634 and BvCESA2, XP_010678670.

### RNA-seq expression profiling of *TaCesA* genes

Gene expression profiling for 21 of the wheat *CesA* genes was performed using publicly available RNA-seq data from two different databases (http://wheat-urgi.versailles.inra.fr/Seq-Repository/RNA-Seq) at McGill University and Genome Quebec Innovation Center. First dataset was a non-oriented library with five wheat organs analysed in duplicates at three development stages for each of the organs. The five organs taken into consideration with respect to developmental stages were root (at seedling, three leaves, and meiosis stages), leaf (seedling, three tillers, and 2 days after anthesis), stem (spike at 1 cm, 2 nodes, and anthesis), spike (2 nodes, meiosis, and anthesis) and grain (2, 14, and 30 days after anthesis). The second dataset was the oriented library with five wheat organs (root, leaf, stem, spike, and grain) with five conditions pooled for 4 lines per organ [36].

The abundance of transcripts from RNA-Seq data was reported using the estimated counts quantified by a programme Kallisto (v0.42.1) [37]. Counts-per-million reads were obtained using Bioconductor's edgeR [38]. Ward’s linkage method was applied to the matrix of Pearson's correlation distances for cluster analysis. Heat map of the candidate transcripts was reported by log2 counts per million (CPM) standard deviation [39].

## Results

### Identification and mapping of *CesA* gene family in wheat

We queried the Chromosome Survey Sequence (CSS) (http://plants.ensembl.org/Triticum_ aestivum/Info/Index) generated by the International Wheat Genome Sequencing Consortium to identify the orthologs of various *CesA* genes from bread wheat corresponding to the barley *CesA* sequences [32]. Twenty-two *TaCesA* genes were isolated, six of which were partial (S1 Text). The identified genes were named following the nomenclature of barley, which shares synteny with wheat. To simplify the nomenclature, we attached a suffix corresponding to the specific wheat genome identifier (A, B, or D) at the end of the gene number. For example, *CesA1* in genomes A, B, and D is named as *TaCesA1A*, *TaCesA1B*, and *TaCesA1D*, respectively. As expected, we found three copies for majority of the nine *CesA* orthologs corresponding to the barley genes. For *CesA6*, *7*, and *8* we were able to find only two *CesA* homoeologs. Only one copy was identified for *TaCesA9*. The missing homoeolog of *CesA6* belonged to the D genome but we obtained it from the D genome progenitor *Aegilops tauschii*. The *TaCesA7* homoeolog, which was absent in the A genome, was recovered from the A genome progenitor *Triticum urartu*. We were unable to find the A genome copy of *TaCesA8* from bread wheat as well as the A genome donor, *Triticum urartu*. The three homoeologous copies of each of the *CesA* genes shared 95–99% sequence identity. Different *CesA* genes within a species possessed two highly variable class-specific regions (C-SR-I and C-SR-II) that differentiated them from each other. The wheat orthologs of the CESA proteins of other species exhibited a similarity of 70–80% at the amino acid level with Arabidopsis and 90–95% with rice and barley. The *TaCesA* genes ranged from 4044 to 5251 bp in length and contained 9–13 introns. The ensembl IDs of all the newly identified wheat *CesA* genes are given in Table 1.

**Table 1:** *CesA* genes and their chromosomal locations in hexaploid wheat.

*CesA* gene	Map position (MB)	Ensembl ID
*TaCesA1A*	NA	Traes_2AS_665AF9500.1
*TaCesA1B*	23.50	Traes_2BS_064B02A89.3
*TaCesA1D*	18.70	Traes_2DS_C80293002.1
*TaCesA2A*	176.7	Traes_4AL_941C0E3EF.2
*TaCesA2B*	262.70	Traes_5BL_3A1A752B7.1
*TaCesA2D*	151.78	Traes_5DL_3B0E69498.2
*TaCesA3A*	125.80	Traes_5AL_E176291CC.1
*TaCesA3B*	247.07	Traes_5BL_CFCBFDA99.2
*TaCesA3D*	144.92	Traes_5DL_BBFD06D43.1
*TaCesA4A*	93.16	Traes_1AL_F420A1BBE.1
*TaCesA4B*	48.70	Traes_1BL_B34FCB150.1
*TaCesA4D*	NA	Traes_1DL_129574E44.1/ EMT11949
*TaCesA5A*	29.31	Traes_1AS_10C467127.1
*TaCesA5B*	103.00	Traes_1BS_64E9CC6E0.1
*TaCesA5D*	NA	Traes_1DS_65C1FDCD8.2
*TaCesA6A*	9.23	Traes_6AS_CF6D8CD28.2
*TaCesA6B*	25.84	Traes_6BS_8DA635027.1
*TaCesA7B*	514.04	TRAES3BF028900030CFD_t1
*TaCesA7D*	42.80	Traes_3DL_B2FD2FBFA
*TaCesA8B*	163.48	Traes_5BL_51C858A97.1
*TaCesA8D*	60.35	Traes_5DL_E82D6D246.2
*TaCesA9B*	NA	Traes_2BS_9B34A7A43.2

NA- Precise location of these genes on the respective chromosomes is not known because of the incomplete assembly of the wheat genome.

The newly identified wheat *CesA* genes were mapped to respective chromosomes based on the physical mapping information available in the wheat IWGSC survey sequence annotation database (http://www.wheatgenome.org/). As expected the chromosomal locations of different *CesA* genes followed the trend reported earlier in the syntenic species barley [18]. *TaCesA4A*, *B*, and *D* mapped in respective genomes to chromosome 1; *TaCesA7B* and *D* to chromosome 3; and *TaCesA8B* and *D* to chromosome 5. Similarly, the homoeologs of *TaCesA1*, *2*, *3*, *5*, and *6* mapped to chromosomes 2, 5, 5, 1 and 6 of the respective genomes. However, *TaCesa9B* mapped to chromosome 2B, unlike its ortholog from barley, which is located on chromosome 6. The approximate location of *TaCesA* genes and their homoeologs is presented in Table 1.

### DNA sequence comparison of primary and secondary cell wall *TaCesA* genes

**Fig 2:**
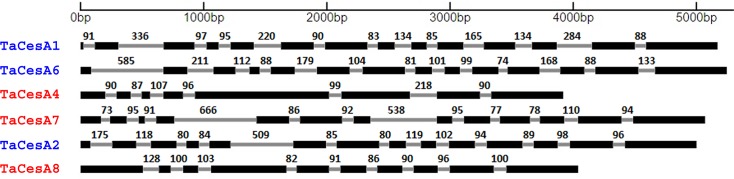
Structural features of the *TaCesA* genes. Drawn to scale, exons are represented by black boxes and introns by grey lines. Intron lengths are presented on top of each intron. PCW and SCW *CesA* genes are shown in blue and red colors, respectively.

On average, a PCW forming gene was longer than the one involved in SCW formation. The longest gene, *TaCesA6*, was 5251 base pairs (bp) and the shortest, *TaCesA4*, was 3923 bp in length. The size variations among different *CesA* genes arose mainly from the number and length of introns (Table 2). *TaCesA1*, *2*, and *6* had 13 introns each, whereas *TaCesA4*, 7 and *8* had 7, 12, and 9 introns, respectively (Fig 2).

**Table 2:** Structures of the *TaCesA* genes for PCW and SCW synthesis.

*CesA* gene	PCW or SCW	Gene length (nt)	Introns (#)	ORF length (AA)	Map to Chromosome
*TaCesA1*	PCW	5175	13	1080	2
*TaCesA2*	PCW	5005	13	1091	5
*TaCesA3*	PCW	5127	13	1105	5
*TaCesA4*	SCW	3923	7	1044	1
*TaCesA5*	PCW	4,085	14	1078	1
*TaCesA6*	PCW	5251	13	1075	6
*TaCesA7*	SCW	5072	12	991	3
*TaCesA8*	SCW	4044	9	1055	5
*TaCesA9*	PCW	2184	5	537	2

The introns in PCW *TaCesA1*, *2*, and *6* accounted for 1732–2026 bp of the genes, approximately double that of the 791 and 879 bp for the SCW *TaCesA4* and *8* genes. One of the SCW genes, *TaCesA7*, possessed a large total intronic region of 2095 bp, which was similar to the PCW *TaCesA* genes. Exonic regions in all the PCW forming genes (~3.2 kb) were similar in length to those of the SCW forming genes (2.9–3.2kb). Exon-intron boundaries were random in all the genes studied, which was in contrast to the conserved boundaries reported in other species [40]. The PCW and SCW genes, across groups, were 45–52% similar. Sequence similarity within the PCW and SCW groups was 54–56% and 46–63% respectively.

### Evolution of introns in *TaCesA* gene family

**Fig 3:**
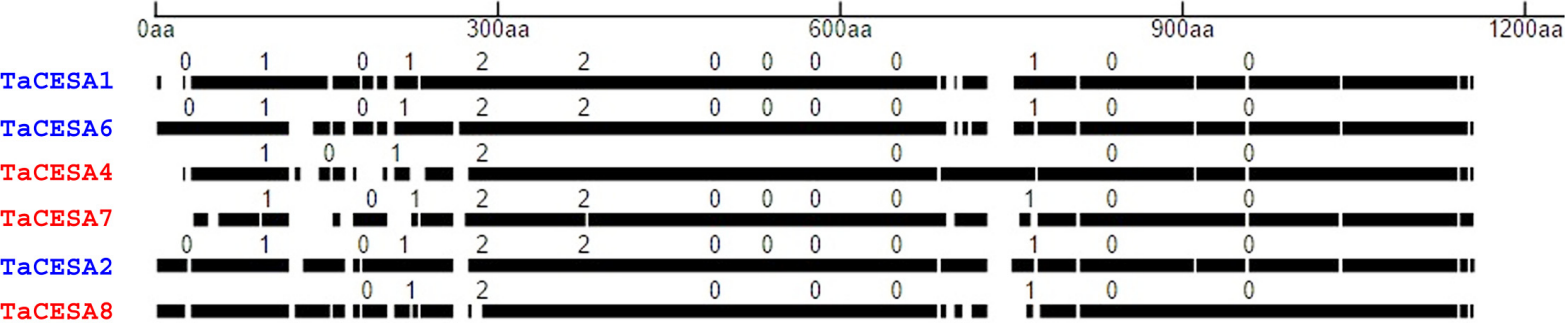
Amino acid sequence alignment of wheat CESA proteins. Drawn to scale with solid lines representing conserved amino acid sequences and the gaps representing the mismatches and deletions. Corresponding phases of intron evolution (0, 1, 2) for the CESA proteins are shown on the top of the solid lines. Primary and secondary cell wall CESAs are shown in blue and red color, respectively.

Three different phases of intron evolution were predicted. Phase 0, 1, or 2 referred to the insertion of an intron between two consecutive codons, between the first and the second base or second and the third base of a codon, respectively [41]. In PCW *TaCesA* genes, all of the introns had identical phase distributions: introns 1, 3, 7, 8, 9, 10, 12, and 13 occurred in 0 phase, introns 2, 4, and 11 were in phase 1, and introns 5 and 6 occurred in phase 2. In contract, SCW *TaCesA* genes exhibited variable patterns of intron phase distribution. Introns 2, 5, 6, and 7 in *CesA4* had 0 phase distribution, introns 1 and 3 had 1, and intron 4 had a phase distribution of 2. *TaCesA7* also had introns with all three types of phase distribution; introns 2, 6, 7, 8, 9, 11, 12 were in phase 0, introns 1, 3, and 10 in phase 1, and introns 4 and 5 in phase 2. *CesA8* similarly had introns 1, 4, 5, 6, 8, and 9 in phase 0, introns 2 and 7 in phase 1, and intron 3 in phase 2 (Fig 3). The largest proportion of introns (57–66%) in all the studied genes was found to be in phase 0, followed by phase 1 (22–28%) and phase 2 (11–16%).

### Amino acid variability of predicted TaCESA proteins

The predicted size of PCW and SCW TaCESAs ranged between 1075–1091 and 991–1055 amino acids (AA), respectively. To identify group-specific changes in primary and secondary cell wall CESA proteins, AA sequences from all TaCESAs were aligned. All the complete CESA proteins possessed the already known, specific CESA domains, such as a ZnF (CX2-CX14-ACX2-CX4-CX2-CX7-GX3-CX2-C) near the N-terminus of the derived amino acid sequence (S2 Text). All the TaCESAs possessed eight TMDs; two towards the N-terminus and six near the C-terminus, as well as the conserved D, DXD, D, QXXRW signatures (Fig 1).

Major differences among TaCESAs resulted from the deletion of up to 45 AAs in hypervariable regions. The N-terminii of the PCW TaCESAs possessed more highly conserved motifs and fewer deletions in comparison to the SCW TaCESAs. ZnF consisted of 46 AAs in the predicted TaCESAs, with the exception of an 8 AAs deletion in TaCESA7 and its homoeologs, resulting in the following domain: CX2-CX6-ACX2-CX4-CX2-CX7-GX3-CX2-C as compared to the known domain (CX2-CX12-FXACX2-CX2PXCX2-CXEX5-GX3-CX2C), where X is any amino acid [42]. Four of the TaCESAs out of 22 were missing the ZnF as did TaCESA9 because they were incomplete on the N-terminal end.

### New motifs distinguishing PCW CESAs from SCW CESAs

**Fig 4:**
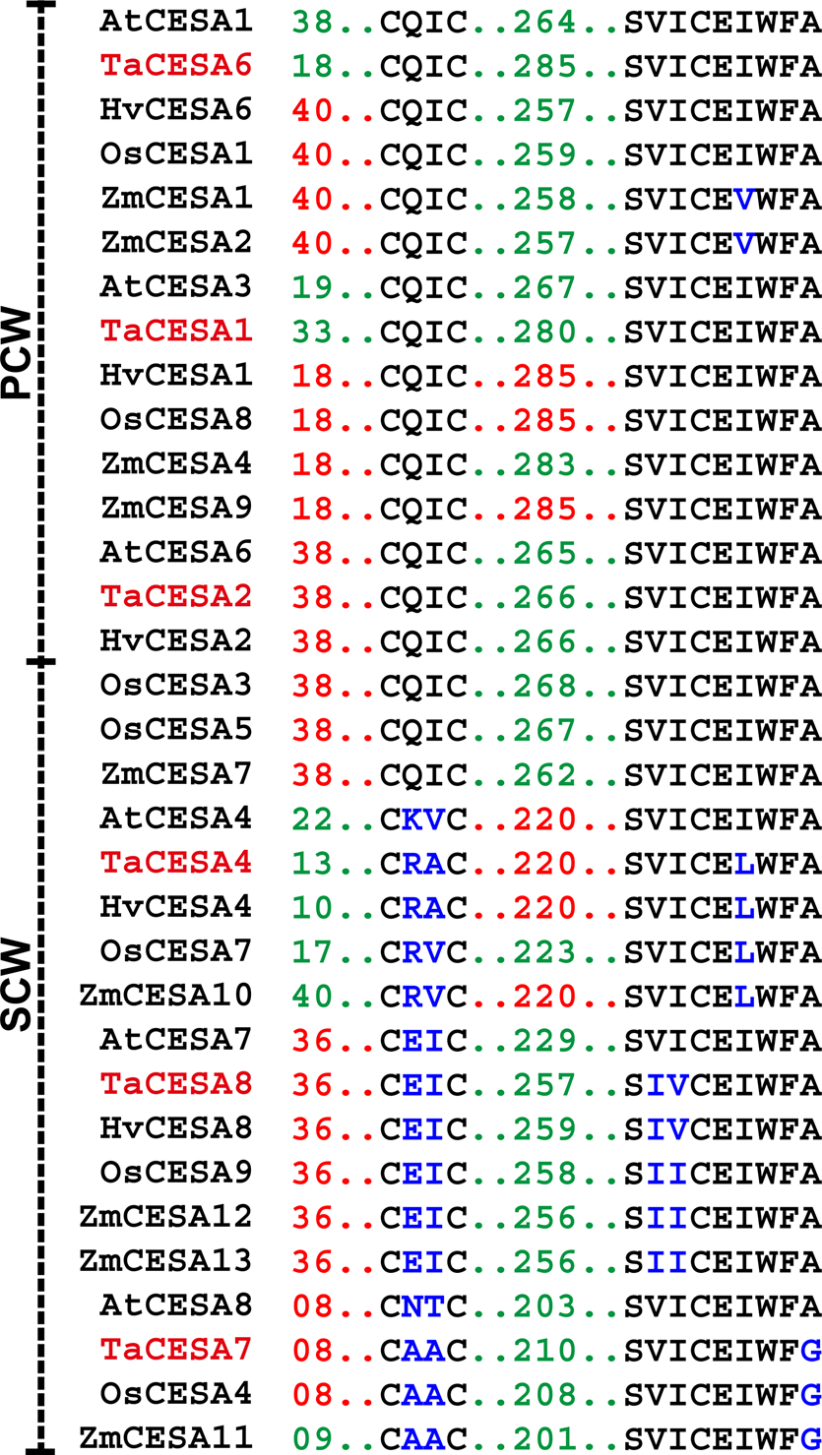
Motifs differentiating PCW and SCW CESA orthologs from different species. Amino acid changes in the motifs are shown in blue.

A new motif distinguishing the PCW CESAs from the SCW CESAs was found within the ZnF. The motif, CQIC, was identified within the small motif, CXXC, reported earlier for differentiating CESAs from the CSL genes [8]. This motif was present in all the PCW TaCESAs. Although SCW TaCESAs also possessed a "CXXC" motif, the two middle amino acids in these proteins were variable. In the SCW TaCESA4, the polar amino acid glutamine was replaced by the negatively charged amino acid, glutamate; in TaCESA7, both the amino acids were replaced by the marginally hydrophobic amino acid alanine; and in TaCESA8, glutamine was replaced by a highly basic (positively charged) amino acid, arginine, and isoleucine was replaced by a relatively conservative substitution of alanine (Fig 4). Another conserved motif, SVICEXWFA, was located within the second transmembrane domain in all the PCW CESAs. In the SCW-specific CESAs, TaCESA4, 7, and 8 this motif was variable but all the amino acid replacements were conservative. For example, isoleucine, a hydrophobic amino acid next to glutamate was replaced by an iso-amino acid, leucine, in CESA4; alanine was replaced by glycine, both somewhat hydrophobic, in CESA7; and valine and isoleucine, both hydrophobic amino acids, switched places in CESA8.

### Conservation of motifs in monocots and dicots

The two motifs, CQIC and SVICEXWFA, distinguished the PCW from the SCW CESAs (Fig 4). That these motifs were conserved was confirmed by analysing the CESA proteins in the PCW and SCW groups from dicot (Arabidopsis) and monocot (barley, maize, rice, and wheat) species. Alignment results demonstrated that the CQIC and SVICEXWFA motifs were completely conserved only in the PCW-specific CESAs in all the plant species studied. The completely conserved amino acid residues in each motif across all the CESA proteins were CXXC and SXXCEXWF (Fig 1).

### Unique motifs conserved among the CESA orthologs from different species

**Fig 5:**
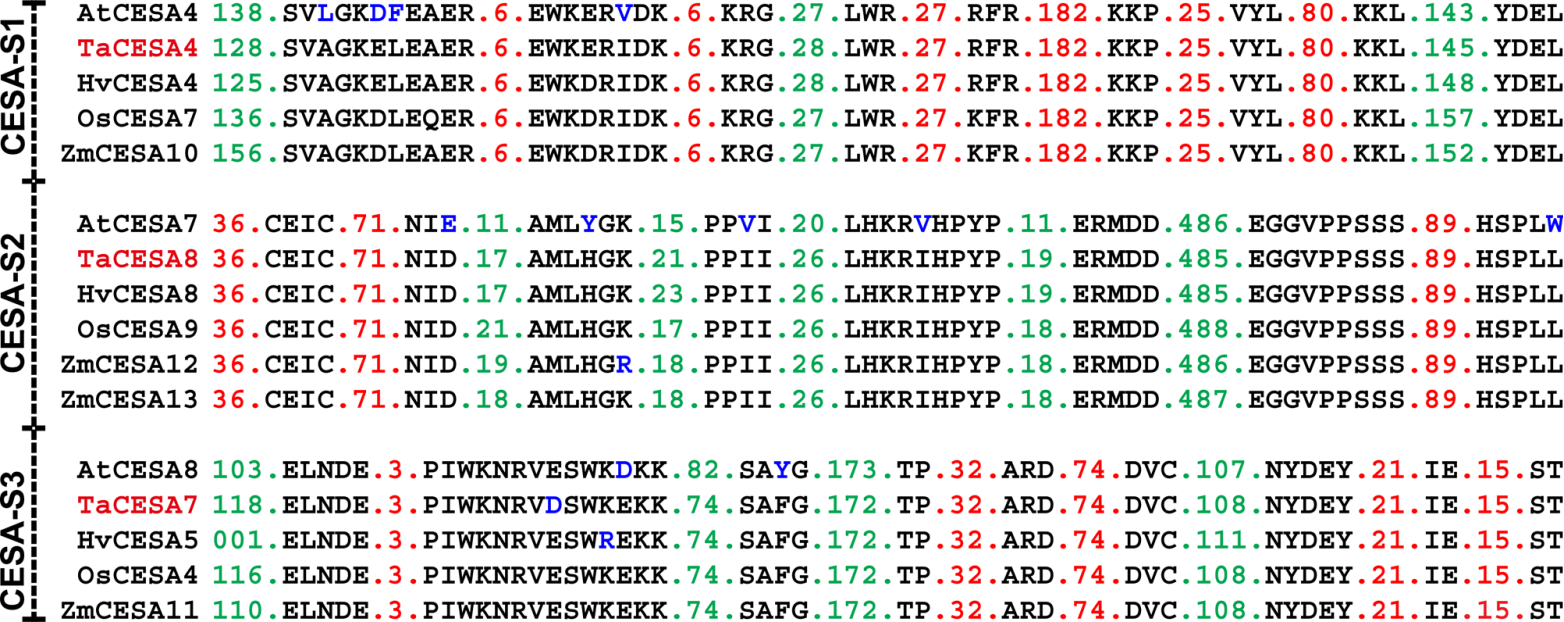
Conserved motifs differentiating the orthologs of SCW CESAs from *Triticum aestivum* (TaCESA), *Arabidopsis thaliana* (AtCESA), *Hordeum vulgare* (HvCESA), *Oryza sativa* (OsCESA), and *Zea mays* (ZmCESA). Amino acid changes in the motifs are shown in blue.

Motif analysis was performed by aligning CESA proteins from Arabidopsis, barley, maize, rice and wheat. Arabidopsis CESA4 and its orthologs from wheat, barley, maize, and rice exhibited 73–74% sequence similarity. In the case of SCW, nine motifs ranging from 2–15 amino acid residues in length provided ortholog-specific identity to the SCW CESAs from different species (Fig 5). These motifs were highly conserved among the orthologs from the five species analysed in this study. Only one gene from each species, with the exception of maize which had two closely related copies for one of the three SCW genes (CESA12 and 13), shared these motifs including a dicot, Arabidopsis. This suggests that the genes for SCW had already duplicated before the separation of monocots and dicots. The number of amino acid residues among most of these motifs was also conserved among different species (Fig 5). CESA7 and 8 from wheat showed 71–75% and 77–79% sequence similarity with the corresponding orthologs from different species, respectively. Although the motifs were unique for CESA4, 7 and 8, they were highly conserved among the orthologs form different species (Fig 5).

**Fig 6:**
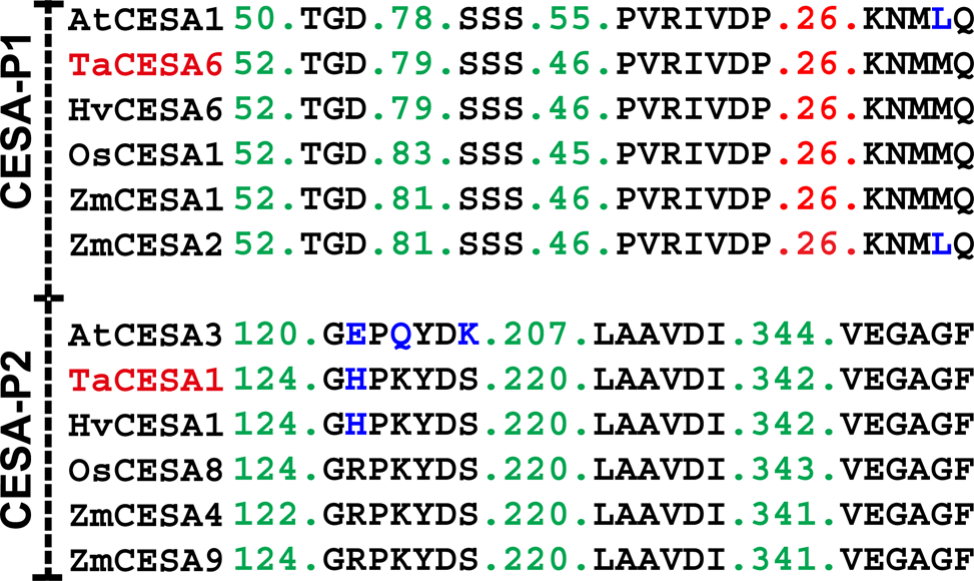
Conserved motifs differentiating the orthologs of PCW CESAs from *Triticum aestivum* (TaCESA), *Arabidopsis thaliana* (AtCESA), *Hordeum vulgare* (HvCESA), *Oryza sativa* (OsCESA), and *Zea mays* (ZmCESA). Amino acid changes in the motifs are shown in blue.

Two PCW CESAs, AtCESA1, 3 and their orthologs from other species differed from AtCESA6 and its orthologs in structural features. AtCESA1 and 3 were highly similar (77–79%) to the corresponding orthologs from barley, maize, rice and wheat. Four motifs in TaCESA6 and three in TaCESA1 orthologs differentiated them from each other and all other CESAs (Fig 6).

### Motifs differentiating CESAs from monocots and dicots

**Fig 7:**
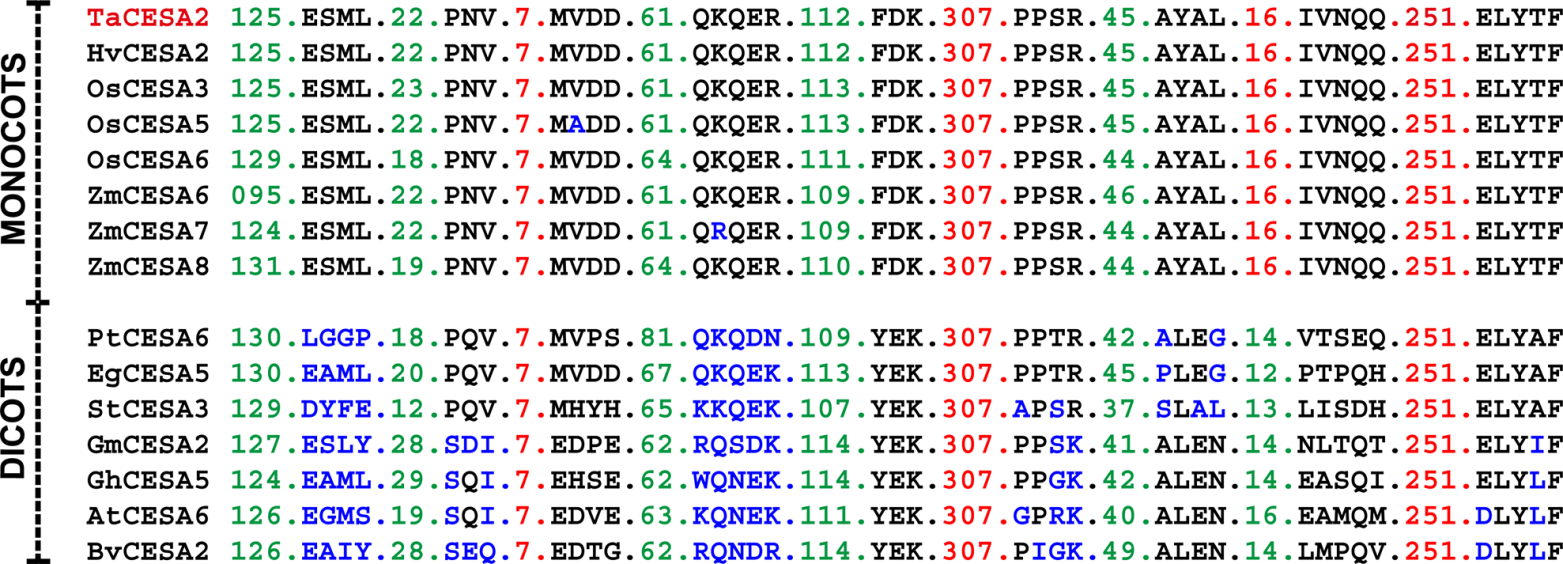
Motifs of CESA orthologs from *Triticum aestivum* (TaCESA), *Arabidopsis thaliana* (AtCESA), *Beta vulgaris* (BvCESA), *Eucalyptus grandis* (EgCESA), *Glycine max* (GmCESA), *Gossypium hirsutum* (GhCESA), *Hordeum vulgare* (HvCESA), *Oryza sativa* (OsCESA), *Populus trichocarpa* (PtCESA), *Solanum tuberosum* (StCESA) and *Zea mays* (ZmCESA). Conserved and non-conserved amino acids residues are highlighted in red and green respectively. Amino acid changes in the motifs are highlighted in blue.

Arabidopsis CESA6 and its orthologs from other species in this study exhibited 68–70% sequence similarity but lacked any specific patterns that could differentiate them from the other CESAs. However, this group possessed motifs that were only conserved in the orthologs from monocots (grasses) but not in Arabidopsis. To confirm the specificity of these motifs for grasses, we retrieved the sequences of TaCESA2 orthologs from seven dicot species: *Arabidopsis thaliana* (AtCESA6), *Beta vulgaris* (BvCESA2), *Eucalyptus grandis* (EgCESA5), *Glycine max* (GmCESA2), *Gossypium hirsutum* (GhCESA5), *Populus trichocarpa* (PtCESA6) and *Solanum tuberosum* (StCESA3). The CESA2 and its orthologs from grasses were compared with its orthologs from dicot species. For this particular gene nine motifs were highly conserved in the orthologs from grasses (Fig 7). But in dicots, these motifs were replaced by variable amino acid residues.

### Phylogenetic analysis

**Fig 8:**
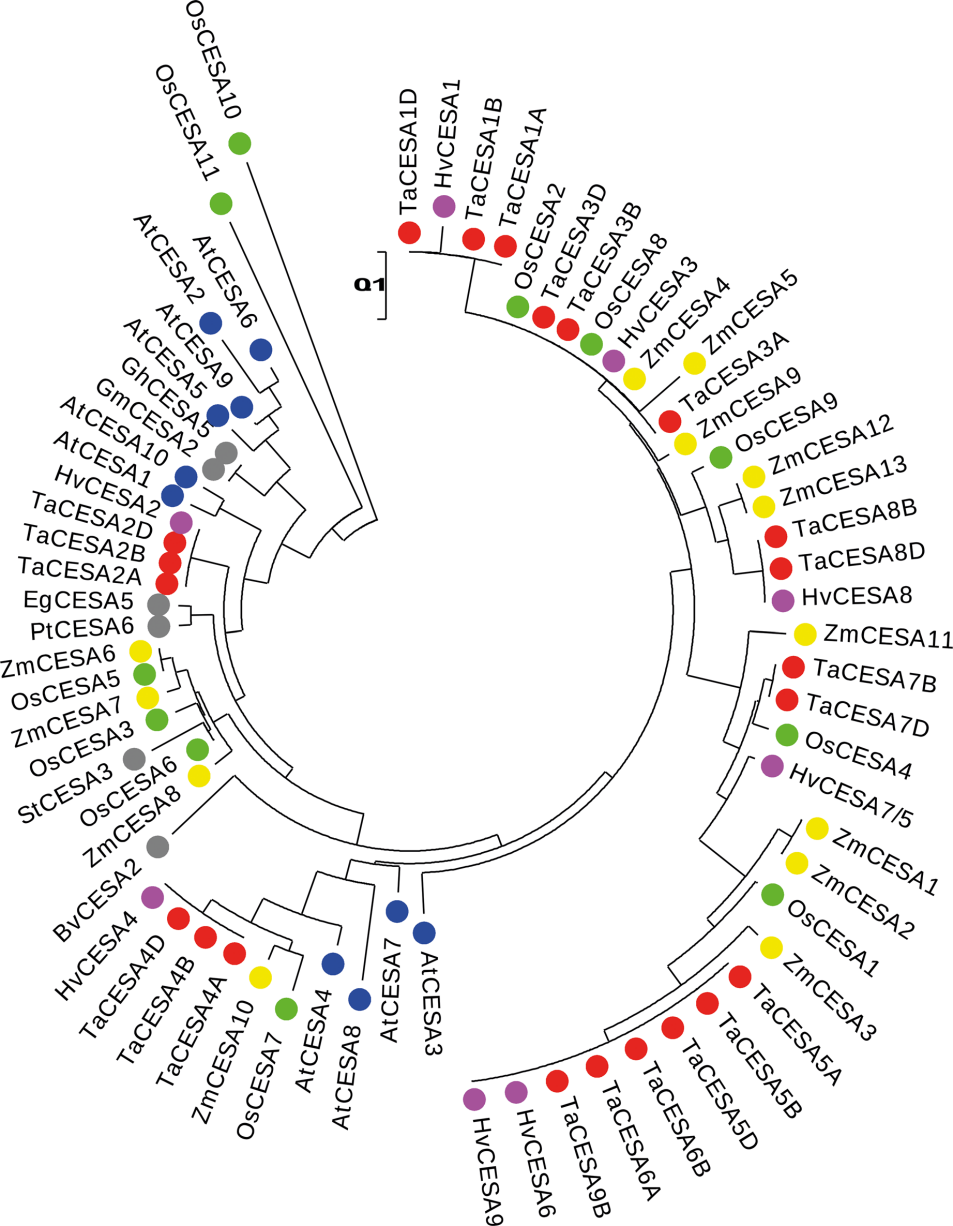
Unrooted phylogenetic tree of the CESAs from *Triticum aestivum* (TaCESA), *Arabidopsis thaliana* (AtCESA), *Beta vulgaris* (BvCESA), *Eucalyptus grandis* (EgCESA), *Glycine max* (GmCESA), *Gossypium hirsutum* (GhCESA), *Hordeum vulgare* (HvCESA), *Oryza sativa* (OsCESA), *Populus trichocarpa* (PtCESA), *Solanum tuberosum* (StCESA) and *Zea mays* (ZmCESA). The bar provides a scale for the branch length in the horizonal dimension. The line segment with the number '0.1' means that an equal length of the branch between the CESA proteins represents a change of 0.1 AA. Color codes for different species: Red—TaCESA, blue–AtCESA, purple—HvCESA, yellow—ZmCESA, green—OsCESA, and grey–BvCESA, EgCESA, GmCESA, GhCESA, PtCESA, StCESA.

The evolutionary history of the CESAs was inferred from the analysis involving 70 CESA protein sequences from different species. An unrooted phylogenetic tree revealed that the orthologs from Arabidopsis, barley, beet, cotton, maize, poplar, potato, rice, rose gum, soybean and wheat were grouped together. Branch lengths, which are indicative of the evolutionary distances were used to interpret the phylogenetic tree (Fig 8). The paralogs from various species were grouped in different clades from those of the orthologs. This suggests, again, that divergence of the *CesA* genes had occurred prior to the separation of monocots and dicots.

### RNA-seq analysis of *TaCesA* genes

**Fig 9:**
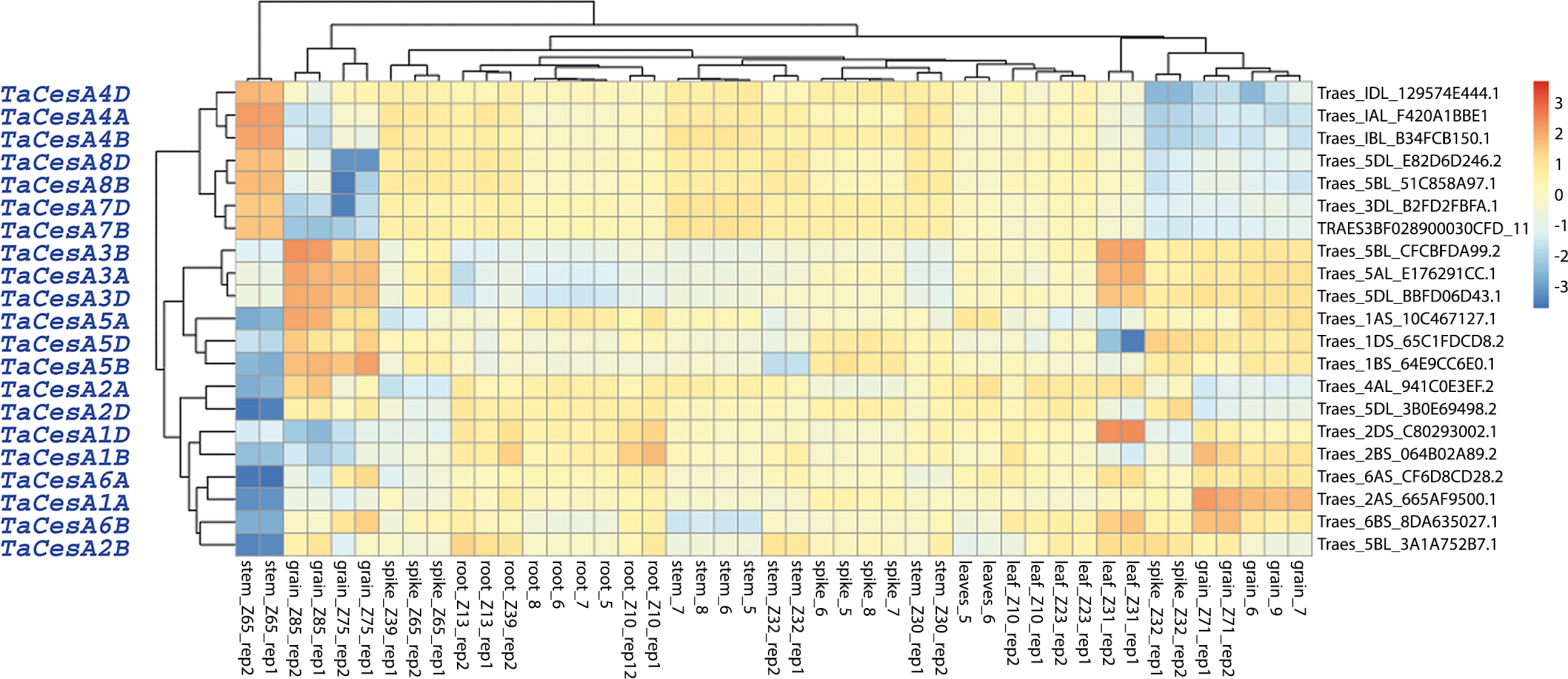
Heat map of 21 *TaCesA* transcripts expressed as deviation of log2 counts per million (CPM) standard deviation in hexaploid wheat.

Gene expression of 21 of the 22 *TaCesA* genes was studied in five organs at three development stages. We left out the *TaCesA9* gene because it was represented by a highly truncated cDNA. A heat map displaying transcript abundance of the *CesA* genes from different wheat tissues is shown in Fig 9.

Transcript abundance data revealed the presence of two distinct groups. Group I consisted of *TaCesA4A*, *B*, *D*, *TaCesA7B*, *D* and *TaCesA8B*, *D* genes, all involved in SCW synthesis. These genes were highly expressed in the mature tissues, for example, stem collected soon after anthesis, and at very low levels in the PCW formation (Fig 9). For example, *TaCesA7B*, *D* and *TaCesA8B*, *D* genes were expressed at extremely low levels in the spike and grain tissues (Fig 9).

Group II comprised the PCW *TaCesA* genes: *TaCesA1*, *2*, *3*, *5* and *6* along with their homoeologs from A, B and D genomes. These genes were expressed at lower levels in the mature tissues and at relatively high levels in the PCW forming cells (Fig 9). For example, all three homoeologous copies of the *TaCesA3* gene were expressed in the grain and the leaf tissues. These genes were expressed moderately in the developing grain, which agrees with grain having a relatively low cell wall fraction. The expression of the *TaCesA5A* and *B* genes was highest in the grain tissues from 14 and 30 DAAs, whereas the *TaCesA5D* was moderately expressed in these tissues. The expression of *TaCesA5D* homoeolog was dramatically lower in the leaf tissues at 2 days after anthesis (DAA), whereas *TaCesA1D* was expressed at higher level. The transcript abundance of *TaCesA1A* was highest in the grain tissues at 2DAAs whereas *TaCesA6B* homoeolog was moderately expressed. The expression level of *TaCesA1B* was moderate in the root and grain tissues.

## Discussion

Cellulose consists of paracrystalline microfibrils of multiple, unbranched beta-1,4-glucan chains, which are synthesized by the individual CESA polypeptides in the plasma membrane-localized rosette. *CesA* is a multigene family consisting of more than eight members in higher plants [43]. Structure and function of the *CesA* genes in wheat remains undocumented. Most studies about structural and functional characterization of *CesAs* have been performed in Arabidopsis [10, 44, 45], maize [2, 19], and rice [46, 47]. Bread wheat, an allohexaploid, has a complex genome, ~17 Gb in size, ~80–90% of which consists of repetitive DNA [29]. The availability of large-scale genomic sequence information and conserved synteny between barley and wheat is valuable in exploring wheat gene function and structure [48]. In barley, the *CesA* gene family consists of nine genes (*HvCesA1* to *HvCesA9*. Three genes, *HvCesA1*, *HvCesA2*, and *HvCesA6*, are expressed during primary wall formation, and another three, *HvCesA4*, *HvCesA7*, and *HvCesA8*, during secondary wall formation [18]. In this report, we document 22 *CesA* genes from wheat, which we identified using a comparative genomics approach using barley sequences as anchors. As expected, most of the *TaCesA* genes each have three paralogs in the homoeologous genomes A, B and D. Four of the 22 genes deviated from this pattern: only one paralog was identified for *TaCesA9*, and two each for *TaCesA6*, *7*, and *8*. One of the genes, *TaCesA2*, had two paralogous copies on chromosomes 5B and 5D but the third on chromosome 4A, which was most likely because of a translocation between chromosomes 5A and 4A (Table 1) [49].

All the CESAs possess domains known to be highly conserved among all the plant species studied thus far [10]. Sequences in the non-conserved domains, however, are useful for the identification of the orthologs of individual *CesA* genes (Table 3). In the case of gene families, it is often difficult to determine true orthology among different species solely based on sequence similarity. Many previous studies reported *CesA* orthologs based on phylogenetic analyses [16, 18]. We supplemented the phylogenetic analysis as a tool for the identification of the *CesA* orthologs by searching for the conserved motifs in addition to the ones already known [49].

**Table 3:** *TaCesA* genes and their orthologs from Arabidopsis, barley, maize, and rice involved in the formation of primary cell wall (PCW) or secondary cell wall (SCW).

Gene Function	Wheat	Barley	Maize	Rice	Arabidopsis
PCW	*CesA5*, *6* and *9*	*CesA6* and *9*	*CesA1*, *2* and *3*	*CesA1*	*CesA1*and *10*
	*CesA1*and *3*	*CesA1*and *3*	*CesA4*, *5*, and *9*	*CesA2*, *8*,*10* and *11*	*CesA3*
	*CesA2*	*CesA2*	*CesA6*, *7*, and *8*	*CesA3*, *5*, and *6*	*CesA2*, *5*, *6*, and *9*
SCW	*CesA4*	*CesA4*	*CesA10*	*CesA7*	*CesA4*
	*CesA8*	*CesA8*	*CesA12* and *13*	*CesA9*	*CesA7*
	*CesA7*	*CesA5* and *7*	*CesA11*	*CesA4*	*CesA8*

Knowledge about the conserved structural motifs that can distinguish *CesA* genes involved in PCW and SCW formation as well as *CesAs* between monocots and dicots is limited. Distinct patterns of intron placement, removal, and the phases of insertion in *TaCesA* genes suggested that the phases of intron insertion remained conserved during the evolution of these genes [50]. Deviation of phase distribution from the expected 33% suggested a bias in intron insertions towards the 0 phase, that is, between the codons rather than within the codons [41].

The motif CQIC in ZnF distinguishes the PCW and SCW CESAs from both the monocots and dicots. Distinct CSCs for the synthesis of primary and secondary cell walls have been reported [44–46]. The high level of conservation of the CQIC motif suggests that it is possibly related to cellulose synthesis. This concurs with the observation in other major gene families, where domains and motifs were conserved during the evolution [44–45].

A similar trend of intron phase distribution and motif conservation was observed when we compared CESA1 of *Arabidopsis thaliana* with its orthologs from angiosperms (*Arabidopsis lyrata*, *Aquilegia coerulea*, *Brachypodium distachyon*, *Carica papaya*, *Citrus clementina*, *Citrus sinensis*, *Cucumis sativus*, *Eucalyptus grandis*, *Glycine max*, *Manihot esculenta*, *Medicago truncatula*, *Mimulus guttatus*, *Oryza sativa*, *Populus trichocarpa*, *Physcomitrella patens*, *Prunus persica*, *Ricinus communis*, *Setaria italica*, *Sorghum bicolor*, *Vitis vinifera*, *Zea mays*), Chlorophytes (*Chlamydomonas reinhardtii*, *Volvox carteri)*, and pteridophyte (*Selaginella moellendorffii*).

We also identified new, highly conserved motifs among the CESA orthologs of five species (Arabidopsis, barley, maize, rice and wheat). Despite the variable protein sequence of each member of the CESA family among the orthologs from various species, the organization of the motifs remained conserved.

RNA-seq expression profiling revealed that the three SCW genes (*TaCesA4*, *7*, *8*) and their homoeologs were co-expressed in the mature stem tissues (Fig 9). This observation provided support for these genes being functionally orthologous to the secondary wall-forming genes from other species, for example, Arabidopsis [10, 44, 45], barley [18], maize [2, 19], and rice [46, 47]. Five genes (*TaCesA1*, *2*, *3*, *5*, *6*) and their homoeologs from the A, B and D genomes of wheat constituted a second group involved in PCW synthesis.

**Fig 10:**
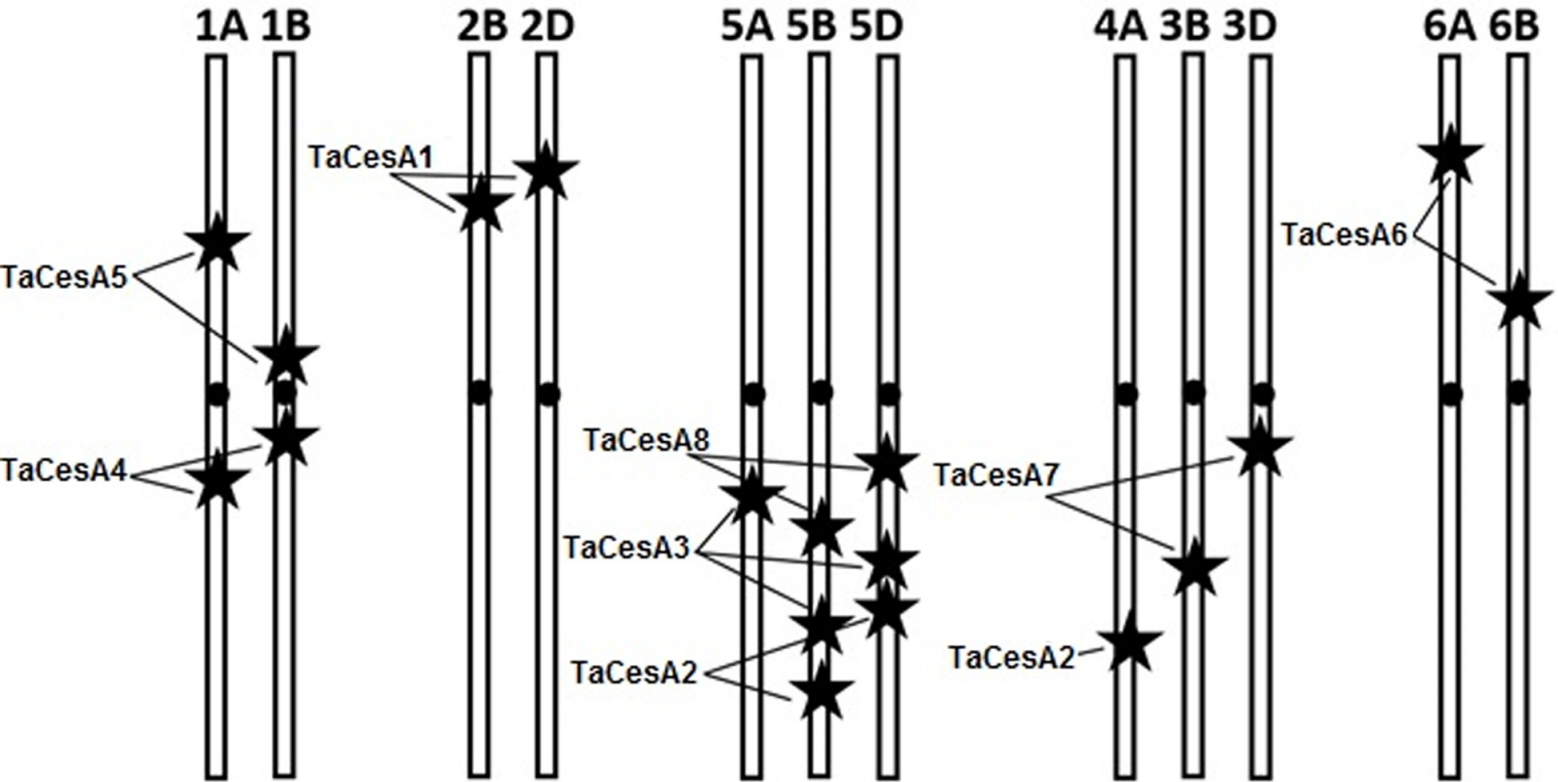
Map positions of *TaCesA* genes in the wheat genome. The exact locations are shown in Table 1.

Most of the *TaCesA* genes were differential expressed among three different genomes of bread wheat, which is a common phenomenon in hexaploid wheat [51]. This differential expression pattern is attributable to the genetic divergence of paralogous genes during the evolution [52]. *TaCesA* genes are distributed across the wheat genome (Fig 10). Similar distribution patterns were observed in Arabidopsis, barley and maize [18, 19].

Our study compiles a list of the *CesA* genes in bread wheat, classified them into PCW and SCW formation, and maps them to the chromosomes. This information will be useful in breeding wheat for culm strength and biofuel-related traits.

## Conclusion

We have identified 22 *CesA* genes from bread wheat and compared them with their orthologs from Arabidopsis, barley, maize, and rice. New structural motifs were identified, which allowed differentiation of the CESA proteins for their roles in primary or secondary wall (PCW or SCW) formation in higher plants. Further characterization of the motifs would be needed, however, to establish their respective biological roles. Several new motifs identified in this study would be useful as signatures for the identification of orthologs of the *CesA* genes from various species. The compilation of the *CesA* gene family in bread wheat along with the expression patterns and genomic map positions of individual members will be helpful in improving culm strength for reduced lodging as well as improving the straw for biofuels.

## Supporting Information

S1 Text Amino acid sequences of wheat *CesA* genes. (DOCX)

S2 Text Amino acid alignments of wheat, barley, rice and maize *CesA* genes. (DOCX)
