# Supplementary material for: Novel Structural and Functional Motifs in cellulose synthase (CesA) Genes of Bread Wheat (Triticum aestivum, L.)
Source: PLoS One. 2016 Jan 15;11(1):e0147046. doi: 10.1371/journal.pone.0147046 (PMC4714848; doi:10.1371/journal.pone.0147046)
Supplement: S1 Text — (DOCX) [file pone.0147046.s001.docx]

TACESA1

>Traes_2AS_665AF9500.1_Triticum_aestivum

MDGDADALKSGRHGAGDVCQICADGLGTTLDGDVFTACDVCRFPVCRPCYEHERKEGTQA

CLQCKTKYKRHRGSPAIRGEEGDDTDADDGSDFNYPASGTEDQKQKIADRMRSWRMNTGG

SGNVGHPKYDSGEIGLSKYDSGEIPRGYVPSVTNSQMSGEIPGASPDHHMMSPTGNISRR

APFPYVNHSPNPSREFSGSIGNVAWKERVDGWKMKQDKGAIPMTNGTSIAPSEGRAATDI

DASTEYNMEDALLNDETRQPLSRKVPIASSKINPYRMVIVLRLVVLSIFLHYRLTNPVRN

AYPLWLLSVICEIWFALSWILDQFPKWFPINRETYLDRLALRYDREGEPSQLAAVDIFVS

TVDPLKEPPIVTANTVLSILAVDYPVDKVSCYVSDDGASMLTFDALAETSEFARKWVPFV

KKYDIEPRAPEFYFCQKIDYLKDKVQPSFVKDRRAMKREYEEFKIRINALVSKALKVPEE

GWIMQDGTPWPGNNTRDHPGMIQVFLGHSGGLDTEGNELPRLVYVSREKRPGFQHHKKAG

AMNALVRVSAVLTNGQYMLNLDCDHYINNSKAVREAMCFLMDPNLGPQVCYVQFPQRFDG

IDRNDRYANRNTVFFDINLRGLDGIQGPVYVGTGCVFNRTAIYGYEPPIKAKKPGFLASL

CGGKKKASKSKKRSSDKKKSNKHVDSSVPVFNLEDIEEGVEGAGFDDEKSVLMSQMSLEK

RFGQSAAFVASTLMEYGGVPQSSTPESLLKEAIHVISCGYEDKSEWGTEIGWIYGSVTED

ILTGFKMHARGWRSVYCMPKRPAFKGSAPINLSDRLNQVLRWALGSVEILFSRHCPLWYG

YGGRLKFLERFAYINTTIYPLTSLPLLVYCILPAICLLTGKFIMPEISNLASIWFIALFL

SIFATGILEMRWSGVGIDEWWRNEQFWVIGGISAHLFAVFQGLLKVLAGIDTNFTVTSKA

NDEEGDFAELYMFKWTTLLIPPTTILIINMVGVVAGTSYAINSGYQSWGPLFGKLFFAFW

VIVHLYPFLKGLMGRQNRTPTIVIVWAVLLASIFSLLWVRVDPFTTRLAGPNIQTCGINC

>Traes_2BS_064B02A89.2_Triticum_aestivum

MDGDADALKSGRHGAGDVCQICADGLGTTVDGEVFTACDVCRFPVCRPCYEHERKEGTQ

ACLQCKTKYKRHRGSPPIRGEEGDDTDADDGSDFNYPASGTEDQKQKIADRMRSWRMNTG

GSGNVGHPKYDSGEIGLSKYDSGEIPRGYVPSVTNSQMSGEIPGASPDHHMMSPTGNISR

RAPFPYVNHSPNPSREFSGSIGNVAWKERVDGWKMKQDKGAIPMTNGTSIAPSEGRAATD

IDASTEYNMEDALLNDETRQPLSRKVPIASSKINPYRMVIVLRLVVLSIFLHYRLTNPVR

NAYPLWLLSVICEIWFALSWILDQFPKWFPINRETYLDRLALRYDREGEPSQLAAVDIFV

STVDPLKEPPIVTANTVLSILAVDYPVDKVSCYVSDDGASMLTFDALAETSEFARKWVPF

VKKYDIEPRAPEFYFCQKIDYLKDKVQPSFVKDRRAMKREYEEFKIRINALVSKALKVPE

EGWIMQDGTPWPGNNTRDHPGMIQVFLGHSGGLDTEGNELPRLVYVSREKRPGFQHHKKA

GAMNALVRVSAVLTNGQYMLNLDCDHYINNSKAVREAMCFLMDPNLGPQVCYVQFPQRFD

GIDRNDRYANRNTVFFDINLRGLDGIQGPVYVGTGCVFNRTAIYGYEPPIKAKKPGFLAS

LCGGKKKTSKSKKRSSDKKKSNKHVDSSVPVFNLEDIEEGVEGAGFDDEKSVLMSQMSLE

KRFGQSAAFVASTLMEYGGVPQSSTPESLLKEAIHVISCGYEDKSEWGTEIGWIYGSVTE

DILTGFKMHARGWRSVYCMPKRPAFKGSAPINLSDRLNQVLRWALGSVEILFSRHCPLWY

GYGGRLKFLERFAYINTTIYPLTSLPLLVYCILPAICLLTGKFIMPEISNLASIWFIALF

LSIFATGILEMRWSGVGIDEWWRNEQFWVIGGISAHLFAVFQGLLKVLAGIDTNFTVTSK

ANDEEGDFAELYMFKWTTLLIPPTTILIINMVGVVAGTSYAINSGYQSWGPLFGKLFFAF

WVIVHLYPFLKGLMGRQNRTPTIVIVWAVLLASIFSLLWVRVDPFTTRLAGPNIQTCGIN

C

>Traes_2DS_C80293002.1_Triticum_aestivum

MASSGSPAIRGEEGDDTDADDGSDFNYPASGTEDQKQKIADRMRSWRMNTGGSGNVGHPK

YDSGEIGLSKYDSGEIPRGYVPSVTNSQMSGEIPGASPDHHMMSPTGNISRRAPFPYVNH

SPNPSREFSGSIGNVAWKERVDGWKMKQDKGAIPMTNGTSIAPSEGRAATDIDASTEYNM

EDALLNDETRQPLSRKVPIASSKINPYRMVIVLRLVVLSIFLHYRLTNPVRNAYPLWLLS

VICEIWFALSWILDQFPKWFPINRETYLDRLALRYDREGEPSQLAAVDIFVSTVDPLKEP

PIVTANTVLSILAVDYPVDKVSCYVSDDGASMLTFDALAETSEFARKWVPFVKKYDIEPR

APEFYFCQKIDYLKDKVQPSFVKDRRAMKREYEEFKIRINALVSKALKVPEEGWIMQDGT

PWPGNNTRDHPGMIQVFLGHSGGLDTEGNELPRLVYVSREKRPGFQHHKKAGAMNALVRV

SAVLTNGQYMLNLDCDHYINNSKAVREAMCFLMDPNLGPQVCYVQFPQRFDGIDRNDRYA

NRNTVFFDINLRGLDGIQGPVYVGTGCVFNRTAIYGYEPPIKAKKPGFLASLCGGKKKAS

KSKKRSSDKKKSNKHVDSSVPVFNLEDIEEGVEGAGFDDEKSVLMSQMSLEKRFGQSAAF

VASTLMEYGGVPQSSTPESLLKEAIHVISCGYEDKSEWGTEIGWIYGSVTEDILTGFKMH

ARGWRSVYCMPKRPAFKGSAPINLSDRLNQVLRWALGSVEILFSRHCPLWYGYGGRLKFL

ERFAYINTTIYPLTSLPLLVYCILPAICLLTGKFIMPEISNLASIWFIALFLSIFATGIL

EMRWSGVGIDEWWRNEQFWVIGGISAHLFAVFQGLLKVLAGIDTNFTVTSKANDEEGDFA

ELYMFKWTTLLIPPTTILIINMVGVVAGTSYAINSGYQSWGPLFGKLFFAFWVIVHLYPF

LKGLMGRQNRTPTIVIVWAVLLASIFSLLWVRVDPFTTRLAGPNIQTCGINC

TACESA2

>Traes_4AL_941C0E3EF.2_Triticum_aestivum

MEASAGLVAGSHNRNELVVIRRDGEPGARPLKQQNRGACQICGDDLGLGPGGDPFVACNE

CAFPVCRDCYEYERREGTQNCPQCKTRYKRLKGCARVPGDEEEDGADDLEDEFNWRDRDD

SQYAAESMLHAHMTYGRGGDLDGVHQPFQPNPNVPLLTNGQMVDDIPPEQHALVPSFVGG

GGKRIHPLPYADSNLPVQPRSMDPSKDIGSYGYGSVAWKERMESWKQKQERLHQARNDGG

KDWNGDGDDADLPLMDEARQPLSRKVPIPSSLINPYRMIIVIRLVIVCLFFHYRVMHPVH

DAFVLWLISVICEIWFAMSWILDQFPKWFPIERETYLDRLTLRFDKEGQPSQLAPVDFFV

STVDPAKEPPLVTANTILSILAVDYPVDKLSCYVSDDGAAMLTFEGLSETSEFAKKWVPF

CKKYSIEPRAPEWYFQQKIDYLKDKVVPNFVRDRRAMKREYEEFKIRINALVAKAQKVPE

EGWTMQDGTPWPGNNVRDHPGMIQVFLGQSGGLDVEGNELPRLVYVSREKRPGYNHHKKA

GAMNALVRVSAVLTNAPYMLNLDCDHYVNNSKAVKEAMCFMMDPLVGKKVCYVQFPQRFD

SIDRHDRYANKNVVFFDINMKGLDGIQGPIYVGTGCVFRRQALYGYDAPKTKKPPSRTCN

CWPKWCVCCFCFGNRKNKKKVTKPKTEKKKRLFFKKEENQSPAYALSEIDEAAAGAETQK

AGIVNQQKLEKKFGQSAVFVASTLLENGGTLRCDSPASLLKEAIHVIGCGYEDKTDWGKE

IGWIYGSVTEDILTGFKMHCHGWRSIYCIPKRPAFKGSAPLNLSDRLNQVLRWALGSIEI

FFSNHCPLWYGYGGGLKFLERFSYINSIVYPWTSIPLLAYCTLPAICLLTGKFITPELSN

LASIWYMSLFICIFATGILEMRWARVAVDDWWRNEQFWVIGGVSAHLFAVFQGLLKVIAG

VDTSFTVTTKAGDDEEFSELYTFKWTTLLIPPTTLLLLNFIGVVAGISNAINNGYESWGP

LFGKLFFAFWVIVHLYPFLKGLLGRQNRTPTIVIVWSILLASIISLLWVRVNPFLAKTDG

PLLEECGLDCT

>Traes_5BL_3A1A752B7.1_Triticum_aestivum

MEASAGLVAGSHNRNELVVIRRDGEPGARPLKQQNRGACQICGDDLGLGPGGDPFVACNE

CAFPVCRDCYEYERREGTQNCPQCKTRYKRLKGCARVPGDEEEDGADDLEDEFNWRDRDD

SQYAAESMLHAHMTYGRGGDLDGVHQPFQPNPNVPLLTNGQMVDDIPPEQHALVPSFVGG

GGKRIHPLPYADSNLPVQPRSMDPSKDIGSYGYGSVAWKERMESWKQKQERLHQARNDGG

KDWNGDGDDADLPLMDEARQPLSRKVPIPSSLINPYRMIIVIRLVIVCLFFHYRVMHPVH

DAFVLWLISVICEIWFAMSWILDQFPKWFPIERETYLDRLTLRFDKEGQPSQLAPVDFFV

STVDPAKEPPLVTANTILSILAVDYPVDKLSCYVSDDGAAMLTFEGLSETSEFAKKWVPF

CKKYSIEPRAPEWYFQQKIDYLKDKVVPNFVRDRRAMKREYEEFKIRINALVAKAQKVPE

EGWTMQDGTPWPGNNVRDHPGMIQVFLGQSGGLDVEGNELPRLVYVSREKRPGYNHHKKA

GAMNALVRVSAVLTNAPYMLNLDCDHYVNNSKAVKEAMCFMMDPLVGKKVCYVQFPQRFD

SIDRHDRYANKNVVFFDINMKGLDGIQGPIYVGTGCVFRRQALYGYDAPKTKKPPSRTCN

CWPKWCVCCFCFGNRKNKKKVTKPKTEKKKRLFFKKEENQSPAYALSEIDEAAAGAETQK

AGIVNQQKLEKKFGQSAVFVASTLLENGGTLRCDSPASLLKEAIHVIGCGYEDKTDWGKE

IGWIYGSVTEDILTGFKMHCHGWRSIYCIPKRPAFKGSAPLNLSDRLNQVLRWALGSIEI

FFSNHCPLWYGYGGGLKFLERFSYINSIVYPWTSIPLLAYCTLPAICLLTGKFITPELSN

LASIWYMSLFICIFATGILEMRWARVAVDDWWRNEQFWVIGGVSAHLFAVFQGLLKVIAG

VDTSFTVTTKAGDDEEFSELYTFKWTTLLIPPTTLLLLNFIGVVAGISNAINNGYESWGP

LFGKLFFAFWVIVHLYPFLKGLLGRQNRTPTIVIVWSILLASIISLLWVRVNPFLAKTDG

PLLEECGLDCT

>Traes_5DL_3B0E69498.2_Triticum_aestivum

MEASAGLVAGSHNRNELVVIRRDGEPGARPLKQQNRGACQICGDDLGLGPGGDPFVACNE

CAFPVCRDCYEYERREGTQNCPQCKTRYKRLKGCARVPGDEEEDGADDLEDEFNWRDRDD

SQYAAESMLHAHMTYGRGGDLDGVHQPFQPNPNVPLLTNGQMVDDIPPEQHALVPSFVGG

GGKRIHPLPYADSNLPVQPRSMDPSKDIGSYGYGSVAWKERMESWKQKQERLHQARNDGG

KDWNGDGDDADLPLMDEARQPLSRKVPIPSSLINPYRMIIVIRLVIVCLFFHYRVMHPVH

DAFVLWLISVICEIWFAMSWILDQFPKWFPIERETYLDRLTLRFDKEGQPSQLAPVDFFV

STVDPAKEPPLVTANTILSILAVDYPVDKLSCYVSDDGAAMLTFEGLSETSEFAKKWVPF

CKKYSIEPRAPEWYFQQKIDYLKDKVVPNFVRDRRAMKREYEEFKIRINALVAKAQKVPE

EGWTMQDGTPWPGNNVRDHPGMIQVFLGQSGGLDVEGNELPRLVYVSREKRPGYNHHKKA

GAMNALVRVSAVLTNAPYMLNLDCDHYVNNSKAVKEAMCFMMDPLVGKKVCYVQFPQRFD

SIDRHDRYANKNVVFFDINMKGLDGIQGPIYVGTGCVFRRQALYGYDAPKTKKPPSRTCN

CWPKWCVCCFCFGNRKNKKKVTKPKTEKKKRLFFKKEENQSPAYALSEIDEAAAGAETQK

AGIVNQQKLEKKFGQSAVFVASTLLENGGTLRCDSPASLLKEAIHVIGCGYEDKTDWGKE

IGWIYGSVTEDILTGFKMHCHGWRSIYCIPKRPAFKGSAPLNLSDRLNQVLRWALGSIEI

FFSNHCPLWYGYGGGLKFLERFSYINSIVYPWTSIPLLAYCTLPAICLLTGKFITPELSN

LASIWYMSLFICIFATGILEMRWARVAVDDWWRNEQFWVIGGVSAHLFAVFQGLLKVIAG

VDTSFTVTTKAGDDEEFSELYTFKWTTLLIPPTTLLLLNFIGVVAGISNAINNGYESWGP

LFGKLFFAFWVIVHLYPFLKGLLGRQNRTPTIVIVWSILLASIISLLWVRVNPFLAKTDG

PLLEECGLDCT

TACESA3

>Traes_5DL_BBFD06D43.1_Triticum_aestivum

IIRRLVSLCCPVFLFSGACPQKSGTGRHGGGQVCQICGDGVGAAADGELFAACDVCAFPV

CRPCYEYERKEGTQACPQCKTKYKRHKGSPPARGDESEDDASDFNYPASANQDQKNKIPE

KMLTWRRNSGASDDIGLTKFGSGEIGLHKYDSGEIPHGYIPRFSHSQVSGEISGASPDHM

MSPAGNAGKRGHPFAYVNHSPNPSREFSGSLGNVAWKERVDGWKMKDKGAIPMTNGTSIA

PSEGRGNGDIDACTDYGMEDPLLNDETRQPLSRKVPIPSSRINPYRMVIVLRLIVLCIFL

HYRITNPVRNAYPLWLLSVICEIWFAFSWILDQFPKWSPVNRETYLDRLALRYDRDGELS

QLAPVDIFVSTVDPMKEPPLVTANTVLSILAVDYPVDKVSCYVSDDGAAMLTFDALAETS

EFARKWVPFCKKYNIEPRAPEWYFAQKIDFLKDKVQTSFVKDRRAMKREYEEFKVRVNSL

VAKAEKVPEEGWIMQDGTPWPGNNTRDHPGMLQVFLGHSGGLDSDGNELPRLVYVSREKR

AGFQHHKKAGAMNALVRVSAVLTNGQYMLNLDCDHYINNSSALREAMCFLMDPNLGRKIC

YVQFPQRFDGIDTNDRYANRNTVFFDINLRGLDGIQGPVYVGTGCVFNRTALYGYEPPMK

SKESGLFSKLCGGRTSKSKSTGSKKSDKHADGSVPMFNLEDIEEGIEGSGFDDEKSLLMS

QMSLEKRFGQSSVFVASTLMEYGGVPQSATPESLLKEAIHVISCGYEDRSDWGREIGWIY

GSVTEDILTGFKMHARGWRSIYCMPKRPAFKGSAPINLSDRLNQVLRWALGSVEILFSRH

CPIWYGYGGRLKFLERFAYINTTIYPLTSIPLLIYCILPAVCLLTGKFIIPQISNIASIW

FISLFISIFATGILEMRWSGVGIDEWWRNEQFWVIGGISAHLFAVFQGLLKVLAGIDTSF

TVTSKASDEDNDFAELYMFKWTTLLIPPTTILIINLVGVVAGTSYAINSGYQSWGPLFGK

LFFAFWVIIHLYPFLKGLMGRQNRTPTIVVVWAILLASIFSLLWVRIDPFTTRVTGPDIR

MCGINC

>Traes_5AL_E176291CC.1_Triticum_aestivum

MMSPAGNVGKRGHPFAYVNHSPNPSREFSGSLGNVAWKERVDGWKMKDKGAIPMTNGTSI

APSEGRGNGDIDACTDYGMEDPLLNDETRQPLSRKVPIPSSRINPYRMVIVLRLIVLCIF

LHYRITNPVRNAYPLWLLSVICEIWFAFSWILDQFPKWSPVNRETYLDRLALRYDRDGEL

SQLAPVDIFVSTVDPMKEPPLVTANTVLSILAVDYPVDKVSCYVSDDGAAMLTFDALAET

SEFARKWVPFCKKYNIEPRAPEWYFAQKIDFLKDKVQTSFVKDRRAMKREYEEFKVRVNS

LVAKAEKVPEEGWIMQDGTPWPGNNTRDHPGMLQVFLGHSGGLDSDGNELPRLVYVSREK

RAGFQHHKKAGAMNALVRVSAVLTNGQYMLNLDCDHYINNSSALREAMCFLMDPNLGRKI

CYVQFPQRFDGIDTNDRYANRNTVFFDINLRGLDGIQGPVYVGTGCVFNRTALYGYEPPM

KNKESGLFSKLCGGRTSKSKSTGSKKSDKHADGSVPVFNLEDIEEGIEGSGFDDEKSLLM

SQMSLEKRFGQSSVFVASTLMEYGGVPQSATPESLLKEAIHVISCGYEDRSDWGREIGWI

YGSVTEDILTGFKMHARGWRSIYCMPKRPAFKGSAPINLSDRLNQVLRWALGSVEILFSR

HCPIWYGYGGRLKFLERFAYINTTIYPLTSIPLLIYCILPAVCLLTGKFIIPQISNIASI

WFISLFISIFATGILEMRWSGVGIDEWWRNEQFWVIGGISAHLFAVFQGLLKVLAGIDTS

FTVTSKASDEDNDFAELYMFKWTTLLIPPTTILIINLVGVVAGTSYAINSGYQSWGPLFG

KLFFAFWVIIHLYPFLKGLMGRQNRTPTIVVVWAILLASIFSLLWVRIDPFTTRVTGPDI

QMCGINC

>Traes_5BL_CFCBFDA99.2_Triticum_aestivum

SGDWSHSAAPSFYFLVRACAQKSGTGRHGGGRVCQICGDGVGAAADGELFAACDVCAFPV

CRPCYEYERKEGTQACPQCKTKYKRHKGSPPARGDESEDDASDFNYPASANQDQKNKVPE

KMLTWRRNSGASDDIGLTKFGSGEIGLHKYDSGEIPHGYIPRFSHSQVSGEISGASPDHM

LSPAGNVGKRGHPFAYVNHSSNPSREFSGSLGNVAWKERVDGWKMKDKGAIPMTNGTSIA

PSEGRGNGDIDACTDYGMEDPLLNDETRQPLSRKVPIPSSRINPYRMVIVLRLIVLCIFL

HYRITNPVRNAYPLWLLSVICEIWFAFSWILDQFPKWSPVNRETYLDRLALRYDRDGELS

QLAPVDIFVSTVDPMKEPPLVTANTVLSILAVDYPVDKVSCYVSDDGAAMLTFDALAETS

EFARKWVPFCKKYNIEPRAPEWYFAQKIDFLKDKVQTSFIKDRRAMKREYEEFKVRVNSL

VAKAEKVPEEGWIMQDGTPWPGNNTRDHPGMLRVFLGHSGGLDSDGNELPRLVYVSREKR

AGFQHHKKAGAMNALVRVSAVLTNGQYMLNLDCDHYINNSSALREAMCFLMDPNLGRKIC

YVQFPQRFDGIDTNDRYANRNTVFFDINLRGLDGIQGPVYVGTGCVFNRTALYGYEPPMK

SKESGLFSKLCGGRTSKSKSTGSKKSDKHADGSVPVFNLEDIEEGIEGSGFDDEKSLLMS

QMSLEKRFGQSSVFVASTLMEYGGVPQSATPESLLKEAIHVISCGYEDRSDWGREIGWIY

GSVTEDILTGFKMHARGWRSIYCMPKRPAFKGSAPINLSDRLNQVLRWALGSVEILFSRH

CPIWYGYGGRLKFLERFAYINTTIYPLTSIPLLIYCILPAVCLLTGKFIIPQISNIASIW

FISLFISIFATGILEMRWSGVGIDEWWRNEQFWVIGGISAHLFAVFQGLLKVLAGIDTSF

TVTSKASDEDNDFAELYMFKWTTLLIPPTTILIINLVGVVAGTSYAINSGYQSWGPLFGK

LFFAFWVIIHLYPFLKGLMGRQNRTPTIVVVWAILLASIFSLLWVRIDPFTTRVTGPDIQ

MCGINC

TACESA4

>Traes_1AL_F420A1BBE.1_Triticum_aestivum

MDTGEPKAKAAKVCRACGDDVGAREDGSPFVACAECGFPVCRPCYEYERSDGTQCCPQCN

TRYKRHKGCPRVEGDEEDGDMDDLEDEFQVKSPKKPHEPVPFDVYSENGEQPPQKWRPGG

PAMSSFGGSVAGKELEAEREMEGSMEWKERIDKWKTKQEKRGKLNRDNSDDDDDDKNDDE

YMLLAEARQPLWRKLPVPSSQINPYRIVIVLRLVVLCFFLRFRIMTPANDAIPLWLVSVI

CELWFALSWILDQLPKWSPVTRETYLDRLALRYDREGEPSRLSPIDFFVSTVDPLKEPPI

ITANTVLSILAVDYPVDRNSCYVSDDGASMLCFDTLSETAEFARRWVPFCKKFAIEPRAP

EFYFSQKIDYLKDKVQPTFVKERRAMKREYEEFKVRINGLVAKAEKKPEEGWVMQDGTPW

PGNNTRDHPGMIQVYLGSQGALDVEGHELPRLVYVSREKRPGHNHHKKAGAMNALVRVSA

VLTNAPFILNLDCDHYVNNSKAVREAMCFLMDPQLGKKLCYVQFPQRFDGIDLHDRYANR

NVVFFDINMKGLDGIQGPVYVGTGCVFNRQALYGYDPPRPEKRPKMTCDCWPSWCCCCCC

FGGGKHRKSDKDKKGGDDEPRRGLLGFYKKRGKKDKLGGGPKKGSYRKQQRGYELEEIEE

GIEGYDELERSSLMSQKSFQKRFGQSPVFIASTLVEDGGLPQGAAADPAGLIKEAIHVIS

CGYEEKTEWGKEIGWIYGSVTEDILTGFKMHCRGWKSVYCTPTRPAFKGSAPINLSDRLH

QVLRWALGSVEIFMSRHCPLWYAYGGRLKWLERFAYTNTIVYPFTSIPLIAYCTIPAVCL

LTGKFIIPTLNNLASIWFIALFMSIIATGVLELRWSGVSIEDWWRNEQFWVIGGVSAHLF

AVFQGFLKVLGGVDTNFTVTSKAGADEADAFGDLYLFKWTTLLIPPTTLIIINMVGIVAG

VSDAVNNGYGSWGPLFGKLFFSFWVIVHLYPFLKGLMGRQNRTPTIVVLWSVLLASIFSL

VWVRIDPFIAKPKGPILKPCGVQC

>Traes_1BL_B34FCB150.1_Triticum_aestivum

MDTGEPKAKAAKVCRACGDDVGTREDGSPFVACAECGFPVCRPCYEYERSDGTQCCPQCN

TRYKRHKGCPRVEGDEEDGDMDDLEDEFQVKSPKKPHEPVPFDVYSENGEQPPQKWRSGG

PAMSSFGGSVAGKELEAEREMEGSMEWKERIDKWKTKQEKRGKLNRDNSDDDDDDKNDDE

YMLRXXXXQPLWRKLPVPSSQINPYRIVIVLRLVVLCFFLRFRIMTPANDAIPLWLVSVI

CELWFALSWILDQLPKWSPVTRETYLDRLALRYDREGEPSRLSPIDFFVSTVDPLKEPPI

ITANTVLSILAVDYPVDRNSCYVSDDGASMLCFDTLSETAEFARRWVPFCKKFAIEPRAP

EFYFSQKIDYLKDKVQPTFVKERRAMKREYEEFKVRINGLVAKAEKKPEEGWVMQDGTPW

PGNNTRDHPGMIQVYLGSQGALDVEGHELPRLVYVSREKRPGHNHHKKAGAMNALVRVSA

VLTNAPFILNLDCDHYVNNSKAVREAMCFLMDPQLGKKLCYVQFPQRFDGIDLHDRYANR

NVVFFDVTPHPPSLPPSDTSCFTPAQIGWIYGSVTEDILTGFKMHCRGWKSVYCTPTRPA

FKGSAPINLTDRLHQVLRWALGSVEIFMSRHCPLWYAYGGRLKWLERFAYTNTIVYPFTS

IPLIAYCTIPAVCLLTGKFIIPTLNNLASIWFIALFMSIIATGVLELRWSGVSIEDWWRN

EQFWVIGGVSAHLFAVFQGFLKVLGGVDTNFTVTSKAGADEADAFGDLYLFKWTTLLIPP

TTLIIINMVGIVAGVSDAVNNGYGSWGPLFGKLFFSFWVIVHLYPFLKGLMGRQNRTPTI

VVLWSVLLASIFSLVWVRIDPFIAKPKGPILKPCGVQC

>Traes_ Traes_1DL_129574E44.1_Triticum_aestivum

MDTGEPKAKAAKVCRACGDDVGTREDGSPFVACAECGFPVCRPCYEYERSDGTQCCPQCN

TRYKRHKGGCPRVEGDEEDGDMDDLEDEFQVKSPKKPHEPVPFDVYSENGEQPPQKWRSG

GPAMSSFGGSVAGKELEAEREMEGSMEWKERIDKWKTKQEKRGKLNRDNSDDDDDDDK

NDIGWIYGSVTEDILTGFKMHCRGWKSVYCTPTRPAFKGSAPINLSDRLHQVLRWALFAYTNTIVYPFTSIPLIAYCTIPAVCLLTGKFIIPTLNNLASIWFIALFMSIIATGVLELRWSGVSIEDWWRNEQFWVIGGVSAHLFAVF NGYGSWGPLFGKLFFSFWVIVHLYPFLKGLMGRQNRTPTIVVLWS

VLLASIFSLVWVRIDPFIAKPKGPIL

TACESA5

>Traes_1AS_10C467127.1_Triticum_aestivum

MAANRGMVAGSHNRNEFVMIRHDGDAPAPGKEVKGAGGQGCQICGDTVGVSATGDVFV

ACNECAFPVCRPCYEYERKDGVKCCPQCKTRYKRLKGSPRVPGDEEEEDVDDLDNEFN

YKQGNGKGPEWQLQGQGEDIDLSSSSRHEPHHRIPRLTSGQQISGEIPDASPDRHSIR

SPTSSYVDPSVPGIPVRIVDPSKDLNSYGLNSVDWKERVESWRVKQDKN

MMQVTNKYPDARGGGDMEGTGSNGEDMQMVDDARLPLSRIVPIPANQLNLYRIVIILRLI

ILCFFFQYRVTHPVRDAYGLWLVSVICEIWFALSWLLDQFPKWYPINRETYLDRLALRYD

REGEPSQLCPIDIFVSTVDPLKEPPLITANTVLSILAVDYPVDKVSCYVSDDGSAMLTFE

SLSETAEFARKWVPFCKKHNIEPRAPEFYFQQKIDYLKDKIQPSFVKERRAMKREYEEFK

IRINALVAKAQKVPEEGWTMADGTAWPGNNPRDHPGMIQVFLGHSGGLDTEGNELPWLVY

VSREKRPGFQHHKKAGAMNALIRVSAVLTNGAYLLNVDCDHYFNSSKALREAMCFMMDPA

LGRKTCYVQFPQRFDGIDLHDRYANRNIVFFDINMKGLDGIQGPMYVGTGCCFNRQALYG

YDPVLTEADLEPNIVVKSCCGGRKKKSKSYMDNKNRMMKRTESSAPIFNMDDIEEGIEGY

EDERSMLMSQKRLEKRFGQSPIFTASTFMTQGGIPPSTNPASLLKEAIHVISCGYEDKTE

WGKEIGWIYGSVTEDILTGFKMHARGWISIYCMPPRPCFKGSAPINLSDRLNQVLRWALG

SVEILFSRHCPIWYNYGGRLKLLERVAYINTIVYPITSLPLIAYCVLPAICLLTNKFIIP

EISNYAGTFFILMFASIFATGILELRWSGVGIEDWWRNEQFWVIGGTSAHLFAVFQGLLK

VLAGIDTNFTVTSKANDEDGDFAELYVFKWTSLLIPPTTVLVINLVGMVAGISYAINSGY

QSWGPLFGKLFFSIWVILHLYPFLKGLMGKQNRTPTIVIVWSILLASIFSLLWVKIDPFI

SDTQKAVAMGQCGVNC

>Traes_1BS_64E9CC6E0.1_Triticum_aestivum

MASNRGMVAGSHNRNEFVMIRHDGDAPAPGKEVKGAGGQGCQICGDTVGVSATGDVFVAC

NECAFPVCRPCYEYERKDGVKCCPQCKTRYKRLKGSPRVPGDEEEEDVDDLDNEFNYKQG

NGKGPEWQLRGQGEDIDLSSSSRHEPHHRIPRLTSGQQISGEIPDASPDRHSIRSPTSSY

VDPSVPVPVRIVDPSKDLNSYGLNSVDWKERVESWRVKQDKNMMQVTNKYPDARGGGDME

GTGSNGEDMQMVDDARLPLSRIVPIPANQLNLYRIVIILRLIILCFFFQYRVTHPVRDAY

GLWLVSVICEIWFALSWLLDQFPKWYPINRETYLDRLALRYDREGEPSQLCPIDIFVSTV

DPLKEPPLITANTVLSILAVDYPVDKVSCYVSDDGSAMLTFESLSETAEFARKWVPFCKK

HNIEPRAPEFYFQQKIDYLKDKIQPSFVKERRAMKREYEEFKIRINALVAKAQKVPEEGW

TMADGTAWPGNNPRDHPGMIQVFLGHSGGLDTDGNELPRLVYVSREKRPGFQHHKKAGAM

NALIRVSAVLTNGAYLLNVDCDHYFNSSKALREAMCFMMDPALGRKTCYVQFPQRFDGID

LHDRYANRNIVFFDINMKGLDGIQGPMYVGTGCCFNRQALYGYDPVLTEADLEPNIVVKS

CCGGRKKKSKSYMDNKNRMMKRTESSAPIFNMDDIEEGIEGYEDERSMLMSQKRLEKRFG

QSPIFTASTFMTQGGIPPSTNPASLLKEAIHVISCGYEDKTEWGKEIGWIYGSVTEDILT

GFKMHARGWISIYCMPPRPCFKGSAPINLSDRLNQVLRWALGSVEILFSRHCPIWYNYGG

RLKLLERVAYINTIVYPITSLPLIAYCVLPAICLLTNKFIIPEISNYAGMFFILMFASIF

ATGILELRWSGVGIEDWWRNEQFWVIGGTSAHLFAVFQGLLKVLAGIDTNFTVTSKANDE

DGDFAELYVFKWTSLLIPPTTVLVINLVGMVAGISYAINSGYQSWGPLFGKLFFSIWVIL

HLYPFLKGLMGKQNRTPTIVIVWSILLASIFSLLWVKIDPFISDTQKAVAMGQCGVNC

>Traes_1DS_65C1FDCD8.2_Triticum_aestivum

MAANRGMVAGSHNRNEFVMIRHDGDAPAPGKEVKGAGGQGCQICGDTVGVSASGDVFVAC

NECAFPVCRPCYEYERKDGVKCCPQCKTRYKRLKGSPRVPGDEEEEDVDDLDNEFNYKQG

NGKGPEWQLRGQGEDIDLSSSSRHEPHHRIPRLTSGQQISGEIPDASPDRHSIRSPTSSY

VDPSVPVPVRIVDPSKDLNSYGLNSVDWKERVESWRVKQDKNMMQVTNKYPDARGGGGDM

EGTGSNGEDMQMVDDARLPLSRIVPIPANQLNLYRIVIILRLIILCFFFQYRVTHPVRDA

YGLWLVSVICEIWFALSWLLDQFPKWYPINRETYLDRLALRYDREGEPSQLCPIDIFVST

VDPLKEPPLITANTVLSILAVDYPVDKVSCYVSDDGSAMLTFESLSETAEFARKWVPFCK

KHNIEPRAPEFYFQQKIDYLKDKIQPSFVKERRAMKREYEEFKIRINALVAKAQKVPEEG

WTMADGTAWPGNNPRDHPGMIQVFLGHSGGLDTDGNELPRLVYVSREKRPGFQHHKKAGA

MNALIRVSAVLTNGAYLLNVDCDHYFNSSKALREAMCFMMDPALGRKTCYVQFPQRFDGI

DLHDRYANRNIVFFDINMKGLDGIQGPMYVGTGCCFNRQALYGYDPVLTEADLEPNIVVK

SCCGGRKKKSKSYMDNKNRMMKRTESSAPIFNMDDIEEGIEGYEDERSMLMSQKRLEKRF

GQSPIFTASTFMTQGGIPPSTNPASLLKEAIHVISCGYEDKTEWGKEIGWIYGSVTEDIL

TGFKMHARGWISIYCMPPRPCFKGSAPINLSDRLNQVLRWALGSVEILFSRHCPIWYNYG

GRLKLLERVAYINTIVYPITSLPLIAYCVLPAICLLTNKFIIPEISNYAGMFFILMFASI

FATGILELRWSGVGIEDWWRNEQFWVIGGTSAHLFAVFQGLLKVLAGIDTNFTVTSKAND

EDGDFAELYVFKWTSLLIPPTTVLVINLVGMVAGISYAINSGYQSWGPLFGKLFFSIWVI

LHLYPFLKGLMGKQNRTPTIVIVWSILLASIFSLLWVKIDPFISDTQKAVAMGQCGVNC

TACESA6

>Traes_6BS_8DA635027.1_Triticum_aestivum

MVAGSHNRNEFVMIRNDGDAPAPGKEVKGTVGQACQICGDTVGVSATGDVFVACNECAFP

VCRPCYEYERKDGVKCCPQCKTRYKRLKGSPRVPGDEEEEDVDDLDNEFNYKQGNGKGPE

WQGEDIDLSSSSRHEPHHRIPRLTSGQQMSGEIPDASPDRHSIRSPTSSYVDPSVPVPVR

IVDPSKDLNSYGLNSVDWKERVESWRVKQDKNMMQVTNKYPDARGGGGDMEGTGSNGEDM

QMVDDARLPLSRIVPIPANQLNLYRIVIILRLIILCFFFQYRVSHPVRDAYGLWLVSVIC

EIWFALSWLLDQFPKWYPINRETYLDRLALRYDREGEPSQLCPIDIFVSTVDPLKEPPLI

TANTVLSILAVDYPVDKVSCYVSDDGSAMLTFESLSETAEFARKWVPFCKKHNIEPRAPE

FYFQQKIDYLKDKIQPSFVKERRAMKREYEEFKIRINALVAKAQKVPEEGWTMADGTAWP

GNNPRDHPGMIQVFLGHSGGLDTDGNELPRLVYVSREKRPGFQHHKKAGAMNALIRVSAV

LTNGAYLLNVDCDHYFNSSKALREAMCFMMDPALGRKTCYVQFPQRFDGIDLHDRYANRN

IVFFDINMKGLDGIQGPMYVGTGCCFNRQALYGYDPVLTEADLEPNIVVKSCCGGRKKKS

KSYMDNKNRMMKRTESSAPIFNMDDIEEGIEGYEDERSMLMSQKRLEKRFGQSPIFTAST

FMTQGGIPPSTNPASLLKEAIHVISCGYEDKTEWGKEIGWIYGSVTEDILTGFKMHARGW

ISIYCMPPRPCFKGSAPINLSDRLNQVLRWALGSVEILFSRHCPIWYNYGGRLKLLERVA

YINTIVYPLTSLPLIAYCVLPAICLLTNKFIIPEISNYAGMFFILMFASIFATGILELRW

SGVGIEDWWRNEQFWVIGGTSAHLFAVFQGLLKVLAGIDTNFTVTSKANDEDGDFAELYV

FKWTSLLIPPTTVLVINLVGMVAGISYAINSGYQSWGPLFGKLFFSIWVILHLYPFLKGL

MGKQNRTPTIVIVWSILLASIFSLLWVKIDPFISDTQKAVAMGQCGVNC

>Traes_6AS_CF6D8CD28.2_Triticum_aestivum

MAANRGMVAGSHNRNEFVMIRNDGDAPAPGKEVKGTVGQACQICGDTVGVSATGDVFVAC

NECAFPVCRPCYEYERKDGVKCCPQCKTRYKRLKGSPRVPGDEEEEDVDDLDNEFNYKQG

NGKGPEWQGEDIDLSSSSRHEPHHRIPRLTSGQQMSGEIPDASPDRHSIRSPTSSYVDPS

VPVPVRIVDPSKDLNSYGLNSVDWKERVESWRVKQDKNMMQVTNKYPDARGGGGDMEGTG

SNGEDMQMVDDARLPLSRIVPIPANQLNLYRIVIILRLIILCFFFQYRVSHPVRDAYGLW

LVSVICEIWFALSWLLDQFPKWYPINRETYLDRLALRYDREGEPSQLCPIDIFVSTVDPL

KEPPLITANTVLSILAVDYPVDKVSCYVSDDGSAMLTFESLSETAEFARKWVPFCKKHNI

EPRAPEFYFQQKIDYLKDKIQPSFVKERRAMKREYEEFKIRINALVAKAQKVPEEGWTMA

DGTAWPGNNPRDHPGMIQVFLGHSGGLDTDGNELPRLVYVSREKRPGFQHHKKAGAMNAL

IRVSAVLTNGAYLLNVDCDHYFNSSKALREAMCFMMDPALGRKTCYVQFPQRFDGIDLHD

RYANRNIVFFDINMKGLDGIQGPMYVGTGCCFNRQALYGYDPVLTEADLEPNIVVKSCCG

GRKKKSKSYMDNKNRMMKRTESSAPIFNMEDIEEGIEGYEDERSMLMSQKRLEKRFGQSP

IFTASTFMTQGGIPPSTNPASLLKEAIHVISCGYEDKTEWGKEIGWIYGSVTEDILTGFK

MHARGWISIYCMPPRPCFKGSAPINLSDRLNQVLRWALGSVEILFSRHCPIWYNYGGRLK

LLERVAYINTIVYPLTSLPLIAYCVLPAICLLTNKFIIPEISNYAGMFFILMFASIFATG

ILELRWSGVGIEDWWRNEQFWVIGGTSAHLFAVFQGLLKVLAGIDTNFTVTSKANDEDGD

FAELYVFKWTSLLIPPTTVLVINLVGMVAGISYAINSGYQSWGPLFGKLFFSIWVILHLY

PFLKGLMGKQNRTPTIVIVWSILLASIFSLLWVKIDPFISDTQKAVAMGQCGVNC

TACESA7

>TRAES3BF028900030CFD_t1_Triticum_aestivum

MEPGAHPPCAACGDDAHAACRACSYTLCKACLDEDVAEGRAACARCGGEYAVSDPANGKG

SAVEEEEAAVEDQLVAEGLRGRVTMANQLSDRQDVVSHARTLSSMSGIGSELNDESGKPI

WKNRVDSWKEKKNEKKASAKKAAAKAQVPPVEEQIMEEKDLTDAYEPLSRIIPISKNKLT

PYRAVIIMRLVVLGLFFHYRITNPVDSAFGLWLTSVICEIWFGFSWILDQFPKWCPVNRE

TYVDRLIARYGDGEDSGLAPVDFFVSTVDPLKEPPLITANTVLSILAVDYPVEKISCYVS

DDGAAMLTFESLAETAEFARRWVPFCKKFSIEPRTPEFYFSQKIDYLKDKIHPSFVKERR

AMKRDYEEFKVRINALVAKAQKTPEEGWVMQDGTPWPGNNSRDHPGMIQVFLGETGARDY

DGNELPRLVYVSREKRPGYQHHKKAGAMNALVRVSAVLTNAPYILNLDCDHYVNNSKAVR

EAMCFMMDPSVGRDVCYVQFPQRFDGIDRSDRYANRNVVFFDVNMKGLDGIQGPVYVGTG

CCFYRQALYGYGPPSLPALPKSSACSFCCCCCPKKKVEKTEKEMHRDSRREDLESAIFNL

REIDNYDEYERSMLISQMSFEKSFGQSSVFIESTLMENGGVPESVDPSTLIKEAIHVISC

GYEEKTEWGKELGWIYGSVTEDILTGFKMHCRGWRSIYCMPIRPAFKGSAPINLSDRLHQ

VLRWALGSVEIFFSRHCPLWYGYGGGRLRWLQRLSYINTIVYPFTSVPLVAYCCLPAICL

LTGKFIIPILSNAATIWFLGLFTSIILTSVLELRWSGIGIEDWWRNEQFWVIGGVSAHLF

AVFQGILKMVIGLDTNFTVTSKAAEDGDFAELYVFKWTTVLIPPTTILVLNLVGVVAGFS

DALNSGYESWGPLFGKVFFAMWVIMHLYPFLKGLMGRQNRTPTIVILWSVLLASVFSLLW

VKIDPFITGAETVATGACSSIDC

>Traes_3DL_B2FD2FBFA.1_Triticum_aestivum

MLDVILVQQDVVSHARTLSSMSGVGSELNDESGKPIWKNRVDSWKEKKNEKKASVKKAAA

KAQVPPVEEQIMEEKDLTDAYEPLSRIIPISKNKLTPYRAVIIMRLVVLGLFFHYRITNP

VDSAFGLWLTSVICEIWFGFSWILDQFPKWCPVNRETYVDRLIARYGDGEDSGLAPVDFF

VSTVDPLKEPPLITANTVLSILAVDYPVEKISCYVSDDGAAMLTFESLAETAEFARRWVP

FCKKFSIEPRTPEFYFSQKIDYLKDKIHPSFVKERRAMKRDYEEFKVRINALVAKAQKTP

EEGWVMQDGTPWPGNNSRDHPGMIQVFLGETGARDYDGNELPRLVYVSREKRPGYQHHKK

AGAMNALVRVSAVLTNAPYILNLDCDHYVNNSKAVREAMCFMMDPSVGRDVCYVQFPQRF

DGIDRSDRYANRNVVFFDVNMKGLDGIQGPVYVGTGCCFYRQALYGYGPPSLPALPKSSA

CSFCCCCCPKKKVEKTEKEMHRDSRREDLESAIFNLREIDNYDEYERSMLISQMSFEKSF

GQSSVFIESTLMENGGVPESVDPSTLIKEAIHVISCGYEEKTEWGKELGWIYGSVTEDIL

TGFKMHCRGWRSIYCMPIRPAFKGSAPINLSDRLHQVLRWALGSVEIFFSRHCPLWYGYG

GGRLRWLQRLSYINTIVYPFTSVPLVAYCCLPAICLLTGKFIIPILSNAATIWFLGLFTS

IILTSVLELRWSGIGIEDWWRNEQFWVIGGVSAHLFAVFQGILKMVIGLDTNFTVTSKAA

EDGDFAELYVFKWTTVLIPPAGFSDALNSGYESWGPLFGKVFFAMWVIMHLYPFLKGLMGR

QNRTPTIVILWSVLLASVFSLLWVKIDPFITGAETVATGACSSIDC

TACESA8

>Traes_5BL_51C858A97.1_Triticum_aestivum

MEAGAGLVAGSHNRNELVLIRGHEDHKPARALSGQVCEICGDEVGRTVDGDLFVACNECG

FPVCRPCYEYERREGTQNCPQCKTRYKRLKGSPRVEGDEDEEDIDDLEHEFNIDDDKQQQ

HGALQNSHITEAMLHGKMSYGRASEDGGEGNNTPMVGIPPIITGNRSMPVSGEFPMSAGH

GHGDFSSSLHKRIHPYPMSEPGSAKWGDEKKEVSWKERMDDWKSKQGIYGAADPDDMDAD

VPLNDEARQPLSRKVSIASSKVNPYRMVIILRLFVLCVFLRYRILNPVPEAIPLWLTSIV

CEIWFAVSWILDQFPKWYPIDRETYLDRLSLRYEREGEPSMLSPVDLFVSTVDPLKEPPL

VTANTVLSILAVDYPVDKVSCYVSDDGASMLSFESLSETAEFARKWVPFCKKFNIEPRAP

EFYFSRKVDYLKDKVQPTFVQERRAMKREYEEFKVRINALVSKAQKVPEEGWIMKDGTPW

PGNNTRDHPGMIQVFLGHSGGLDTEGNELPRLVYVSREKRPGFQHHKKAGAMNALIRVSA

VLTNAPFMLNLDCDHYINNSKAIRESMCFLMDPQVGRKVCYVQFPQRFDGIDAHDRYANR

NTVFFDINMKGLDGIQGPVYVGTGCVFRRQALYGYNPPSGPKRPKMVTCDCCPCFGRKKR

KGGKDGLPEGVADGGMDGDKEQMMSQMNFEKRFGQSAAFVTSTFMEEGGVPPSSSPAALL

KEAIHVISCGYEDKTDWGLELGWIYGSITEDILTGFKMHCRGWRSIYCMPKLAAFKGSAP

INLSDRLNQVLRWALGSVEIFFSRHSPLLYGYKGGNLKWLERFAYINTTIYPFTSLPLLA

YCTLPAVCLLTGKFIMPPVSISTFASLFFISLFISIFATGILELRWSGVSIEEWWRNEQF

WVIGGVSAHLFAVIQGLLKVLAGIDTNFTVTSKATGDEDDEFAELYAFKWTTLLIPPTTL

LVINIIGVVAGISDAINNGYQSWGPLFGKLFFAFWVIVHLYPFLKGLMGRQNRTPTIVII

WSVLLASIFSLLWVRIDPFTVKAKGPDVKQCGINC

>Traes_5DL_E82D6D246.2_Triticum_aestivum

KPARALSGQVCEICGDEVGRTVDGDLFVACNECGFPVCRPCYEYERREGTQNCPQCKTRY

KRLKGSPRVEGDEDEEDIDDLEHEFNIDDDKQLQQHGALQNSHITEAMLHGRMSYGRASE

DGGEGNNTPVSGEFPMSAGYGHGDFSSSMHKRIHPYPMSEPGSAKWGDEKKEVSWKERM

DDWKSKQGIYGAADPDDMDADVPLNDEARKEPPLVTANTVLSILAVDYPVDKVSCYVSDDGA

AMLTFESLSETAEFARKWVPFCKKFNIEPRAPEFYFSRKVDYLKDKVQPTFVQERRAMK

MKREYEEFKVRINALVSKAQKVPEEGWIMKDGTPWPGNNTRDHPGMIQVFLGHSGGLDTE

GNELPRLVYVSREKRPGFQHHKKAGAMNALIRVSAVLTNAPFMLNLDCDHYINNSKAIRE

SMCFLMDPQVGRKVCYVQFPQRFDGIDAHDRYANRNTVFFDINMKGLDGIQGPVYVGTGC

VFRRQALYGYNPPSGPKRPKMVTCDCCPCFGRKKRKGGKDGLPEGVADGGMDGDKEQMMS

QMNFEKRFGQSAAFVTSTFMEEGGVXXXXXXXLKEAIHVISCGYEDKTDWGLELGWIYGS

ITEDILTGFKMHCRGWRSIYCMPKLAAFKGSAPINLSDRLNQVLRWALGSVEIFFSRHSP

LLYGYKGGNLKWLERFAYINTTIYPFTSLPLLAYCTLPAVCLLTGKFIMPPISTFASLFF

ISLFISIFATGILELRWSGVSIEEWWRNEQFWVIGGVSAHLFAVIQGLLKVLAGIDTNFT

VTSKATGDEDDEFAELYAFKWTTLLIPPTTLLVINIIGVVAGISDAINNGYQSWGPLFGK

LFFAFWVIVHLYPFLKGLMGRQNRTPTIVIIWSVLLASIFSLLWVRIDPFTVKAKGPDVK

QCGINC

TACESA9

>Traes_2BS_9B34A7A43.2_Triticum_aestivum

MNALIRVSAVLTNGAYLLNVDCDHYFNSSKALREAMCFMMDPALGRKTCYVQFPQRFDGI

DLHDRYANRNIVFFDINMKGLDGIQGPMYVGTGCCFNRQALYGYDPVLTEADLEPNIVVK

SCCGGRKKKSKSYMDNKNRMMKRTESSAPIFNMDDIEEGIEGYEDERSMLMSQKRLEKRF

GQSPIFTASTFMTQGGIPPSTNPASLLKEAIHVISCGYEDKTEWGKEIGWIYGSVTEDIL

TGFKMHARGWISIYCMPPRPCFKGSAPINLSDRLNQVLRWALGSVEILFSRHCPIWYNYG

GRLKLLERVAYINTIVYPLTSLPLIAYCVLPAICLLTNKFIIPEISNYAGMFFILMFASI

FATGILELRWSGVGIEDWWRNEQFWVIGGTXXXXXXXXXXXXXXXXXXXXXXXXXXNDED

GDFAELYVFKWTSLLIPPTTVLVINLVGMVAGISYAINSGYQSWGPLFGKLFFSIWVILH

LYPFLKGLMGKQNRTPTIVIVWSILLASIFSLLWVKIDPFISDTQKAVAMGQCGVNC
