# Supplementary material for: Novel Structural and Functional Motifs in cellulose synthase (CesA) Genes of Bread Wheat (Triticum aestivum, L.)
Source: PLoS One. 2016 Jan 15;11(1):e0147046. doi: 10.1371/journal.pone.0147046 (PMC4714848; doi:10.1371/journal.pone.0147046)
Supplement: S2 Text — (DOCX) [file pone.0147046.s002.docx]

OsCESA10 1 ------------------------------------------------------------
OsCESA11 1 ------------------------------------------------------------
ZmCESA10 1 MDAGSVTGGLAAGSHMRDELHVMRAREEPNA----KVRSADVKTCRVCADEVGTREDGQP
OsCESA7 1 MDTASVT-----------------------G----GEHKGKEKTCRVCGEEVAAREDGKP
TaCESA4D 1 ----MDT-----------------------G----EPKAKAAKVCRACGDDVGTREDGSP
TaCESA4B 1 ----MDT-----------------------G----EPKAKAAKVCRACGDDVGTREDGSP
TaCESA4A 1 ----MDT-----------------------G----EPKAKAAKVCRACGDDVGAREDGSP
HvCESA4 1 ----MDT-----------------------G----EPKA---KVCRACGDDVGTREDGSP
ZmCESA11 1 -----------------------------------MMESAAAQSCAACGDDA--------
OsCESA4 1 ------------------------------------MMESGVPPCAACGDDA--------
HvCESA7/5 1 ------------------------------------MEPGAHPPCAACGDDA--------
TaCESA7B 1 ------------------------------------MEPGAHPPCAACGDDA--------
TaCESA7D 1 ------------------------------------------------------------
TaCESA8D 1 -----------------------------K-----PARALSGQVCEICGDEVGRTVDGDL
TaCESA8B 1 ---MEAGAGLVAGSHNRNELVLIRGHEDHK-----PARALSGQVCEICGDEVGRTVDGDL
HvCESA8 1 ---MEAGAGLVAGSHNRNELVLIRGHEDHK-----PVRALSGQVCEICGDEVGRTVDGDL
OsCESA9 1 ---MEASAGLVAGSHNRNELVLIRGHEEPK-----PLRALSGQVCEICGDEVGRTVDGDL
ZmCESA12 1 ---MEASAGLVAGSHNRNELVLIRGHEDPK-----PLRALSGQVCEICGDEVGLTVDGDL
ZmCESA13 1 ---MEANAGLVAGSHNRNELVLIRGHEDPK-----PLRALSGQVCEICGDEVGLTVDGDL
OsCESA6 1 ---MEASAGLVAGSHNRNELVVIRRDGGGGGGVGGRRAAEAKAACQICGDDVGEGPDGEP
ZmCESA8 1 ---MEASAGLVAGSHNRNELVVIRRDRESGA-AGGGAARRAEAPCQICGDEVGVGFDGEP
TaCESA2A 1 ---MEASAGLVAGSHNRNELVVIRRDGEPGARPL---KQQNRGACQICGDDLGLGPGGDP
TaCESA2B 1 ---MEASAGLVAGSHNRNELVVIRRDGEPGARPL---KQQNRGACQICGDDLGLGPGGDP
TaCESA2D 1 ---MEASAGLVAGSHNRNELVVIRRDGEPGARPL---KQQNRGACQICGDDLGLGPGGDP
HvCESA2 1 ---MEASAGLVAGSHNRNELVVIRRDGEPGARPL---KQQNRGACQICGDDLGLGPGGDP
ZmCESA7 1 ---MEASAGLVAGSHNRNELVVIRRDGDPGPKPP---REQNGQVCQICGDDVGLAPGGDP
ZmCESA6 1 ---------------------------------M---DQRNGQVCQICGDDVGRNPDGEP
OsCESA3 1 ---MEASAGLVAGSHNRNELVVIRRDGDPGPKPL---RQQNGQVCQICGDDVGLNPDGEP
OsCESA5 1 ---MEASAGLVAGSHNRNELVVIRRDGEPGPKPV---KHTNGQVCQICGDDVGLTPDGEP
TaCESA9B 1 ------------------------------------------------------------
ZmCESA3 1 ---MEANRGMVAGSR--GGVVTIRHDGDGAA-AK-QLKNVNEQICQICGDTLGLSATGDI
ZmCESA1 1 ---MAANKGMVAGSHNRNEFVMIRHDGDVPGSAK-PTKSANGQVCQICGDSVGVSATGDV
ZmCESA2 1 ---MAANKGMVAGSHNRNEFVMIRHDGDAPVPAK-PTKSANGQVCQICGDTVGVSATGDV
OsCESA1 1 ---MAANAGMVAGSRNRNEFVMIRPDGDAPPPAK-PGKSVNGQVCQICGDTVGVSATGDV
TaCESA5A 1 ---MAANRGMVAGSHNRNEFVMIRHDGDAPAPGK-EVKGAGGQGCQICGDTVGVSATGDV
HvCESA6 1 ---MAANRGMVAGSHNRNEFVMIRNDGDAPAPGK-EVKGAGGQACQICGDTVGVSATGDV
TaCESA6B 1 ---------MVAGSHNRNEFVMIRNDGDAPAPGK-EVKGTVGQACQICGDTVGVSATGDV
TaCESA6A 1 ---MAANRGMVAGSHNRNEFVMIRNDGDAPAPGK-EVKGTVGQACQICGDTVGVSATGDV
TaCESA5D 1 ---MAANRGMVAGSHNRNEFVMIRHDGDAPAPGK-EVKGAGGQGCQICGDTVGVSASGDV
TaCESA5B 1 ---MASNRGMVAGSHNRNEFVMIRHDGDAPAPGK-EVKGAGGQGCQICGDTVGVSATGDV
HvCesA9 1 ------------------------------------------------------------
ZmCESA5 1 ---------------------MDG--GDATN----SGKHVAGQVCQICGDGVGTAADGDL
OsCESA2 1 ----------------------------MDG----AKSGKQCHVCQICGDGVGTAADGEL
HvCESA3 1 -----------------------MAGAAKSG----TGRHGAGQVCQICGDGVGAAADGEL
TaCESA3B 1 -------SGDWSHSAAPSFYFLVRACAQKSG----TGRHGGGRVCQICGDGVGAAADGEL
TaCESA3D 1 ------IIRRLV-SLCCPVFLFSGACPQKSG----TGRHGGGQVCQICGDGVGAAADGEL
TaCESA3A 1 ------------------------------------------------------------
HvCESA1 1 ----------------------MDGDADALK----SGRHGAGDVCQICADSLGTTVDGEV
TaCESA1D 1 ------------------------------------------------------------
TaCESA1A 1 ----------------------MDGDADALK----SGRHGAGDVCQICADGLGTTLDGDV
TaCESA1B 1 ----------------------MDGDADALK----SGRHGAGDVCQICADGLGTTVDGEV
OsCESA8 1 ----------------------MDGDADAVK----SGRHGSGQACQICGDGVGTTAEGDV
ZmCESA4 1 ----------------------MEGDADGVK----SGRRGGGQVCQICGDGVGTTAEGDV
ZmCESA9 1 ----------------------MEGDADGVK----SGRRGGGQVCQICGDGVGTTAEGDV
OsCESA10 1 ------------------------------------------------------------
OsCESA11 1 ------------------------------------------------------------
ZmCESA10 57 FVACAECGFPVCRPCYEYERSEGTQCCPQCNTRYKRQK-GCPRVEGDEEEGPEMDDFEDE
OsCESA7 34 FVACAECGFPVCKPCYEYERSEGTQCCPQCNTRYKRHK-GCPRVEGDEDDGGDMDDFEEE
TaCESA4D 30 FVACAECGFPVCRPCYEYERSDGTQCCPQCNTRYKRHKGGCPRVEGDEE-DGDMDDLEDE
TaCESA4B 30 FVACAECGFPVCRPCYEYERSDGTQCCPQCNTRYKRHK-GCPRVEGDEE-DGDMDDLEDE
TaCESA4A 30 FVACAECGFPVCRPCYEYERSDGTQCCPQCNTRYKRHK-GCPRVEGDEE-DGDMDDLEDE
HvCESA4 27 FVACAECGFPVCRPCYEYERSDGTQCCPQCNARYKRHK-GCPRVEGDDE-DGDMDDLEEE
ZmCESA11 18 RAACRACSYALCRACLDEDAAEGRTTCARCGGDYAAIN-PARASEGT---EAEE-EVVEN
OsCESA4 17 HAACRACSYALCKACLDEDAAEGRTTCARCGGEYGAPD-PAHGQGAVVEEEVEE-S---H
HvCESA7/5 17 HAACRACSYALCRACLDEDVAEGRAACARCGGEYAVSD-PAHAKGSAMEEEEEE-AAVED
TaCESA7B 17 HAACRACSYTLCKACLDEDVAEGRAACARCGGEYAVSD-PANGKGSAVEE--EE-AAVED
TaCESA7D 1 ------------------------------------------------------------
TaCESA8D 27 FVACNECGFPVCRPCYEYERREGTQNCPQCKTRYKRLK-GSPRVEGDEDEEDID-DLEHE
TaCESA8B 53 FVACNECGFPVCRPCYEYERREGTQNCPQCKTRYKRLK-GSPRVEGDEDEEDID-DLEHE
HvCESA8 53 FVACNECGFPVCRPCYEYERREGTQNCPQCKTRYKRLK-GSPRVEGDEDEEDID-DLEHE
OsCESA9 53 FVACNECGFPVCRPCYEYERREGTQNCPQCKTRYKRLK-GSPRVPGDEDEEDID-DLEHE
ZmCESA12 53 FVACNECGFPVCRPCYEYERREGTQNCPQCKTRYKRLK-GSPRVAGDDDEEDID-DLEHE
ZmCESA13 53 FVACNECGFPVCRPCYEYERREGTQNCPQCKTRYKRLK-GSPRVAGDDDEEDID-DLEHE
OsCESA6 58 FVACNECAFPVCRNCYDYERREGSQACPQCKTRFKRLK-GCPRVAGDEEEDGVD-DLEGE
ZmCESA8 57 FVACNECAFPVCRACYEYERREGSQACPQCRTRYKRLK-GCPRVAGDEEEDGVD-DLEGE
TaCESA2A 55 FVACNECAFPVCRDCYEYERREGTQNCPQCKTRYKRLK-GCARVPGDEEEDGAD-DLEDE
TaCESA2B 55 FVACNECAFPVCRDCYEYERREGTQNCPQCKTRYKRLK-GCARVPGDEEEDGAD-DLEDE
TaCESA2D 55 FVACNECAFPVCRDCYEYERREGTQNCPQCKTRYKRLK-GCARVPGDEEEDGAD-DLEDE
HvCESA2 55 FVACNECAFPVCRDCYEYERREGTQNCPQCKTRYKRLK-GCARVPGDEEEDGAD-DLEDE
ZmCESA7 55 FVACNECAFPVCRDCYEYERREGTQNCPQCKTRYKRLK-GCQRVTGDEEEDGVD-DLDNE
ZmCESA6 25 FVACNECAFPICRDCYEYERREGTQNCPQCKTRFKRFK-GCARVPGDEEEDGVD-DLENE
OsCESA3 55 FVACNECAFPVCRDCYEYERREGTQNCPQCKTRFKRLR-GCARVPGDEEEDGVD-DLENE
OsCESA5 55 FVACNECAFPVCRDCYEYERREGTQNCPQCKTRFKRLK-GCARVPGDEEEEDVD-DLENE
TaCESA9B 1 ------------------------------------------------------------
ZmCESA3 54 FVACNECAFPVCRPCYEYERKEGNQCCPQCKTRYKRHK-GSPRVRGDEEEDGVD-DLDNE
ZmCESA1 57 FVACNECAFPVCRPCYEYERKEGNQCCPQCKTRYKRQK-GSPRVHGDEDEEDVD-DLDNE
ZmCESA2 57 FVACNECAFPVCRPCYEYERKEGNQCCPQCKTRYKRQK-GSPRVHGDDEEEDVD-DLDNE
OsCESA1 57 FVACNECAFPVCRPCYEYERKEGNQCCPQCKTRYKRHK-GSPRVQGDEEEEDVD-DLDNE
TaCESA5A 57 FVACNECAFPVCRPCYEYERKDGVKCCPQCKTRYKRLK-GSPRVPGDEEEEDVD-DLDNE
HvCESA6 57 FVACNECAFPVCRPCYEYERKDGVQCCPQCKTRYKRLK-GSPRVPGDEEEEDVD-DLDNE
TaCESA6B 51 FVACNECAFPVCRPCYEYERKDGVKCCPQCKTRYKRLK-GSPRVPGDEEEEDVD-DLDNE
TaCESA6A 57 FVACNECAFPVCRPCYEYERKDGVKCCPQCKTRYKRLK-GSPRVPGDEEEEDVD-DLDNE
TaCESA5D 57 FVACNECAFPVCRPCYEYERKDGVKCCPQCKTRYKRLK-GSPRVPGDEEEEDVD-DLDNE
TaCESA5B 57 FVACNECAFPVCRPCYEYERKDGVKCCPQCKTRYKRLK-GSPRVPGDEEEEDVD-DLDNE
HvCesA9 1 ------------------------------------------------------------
ZmCESA5 34 FTACDVCGFPVCRPCYEYERKDGTQACPQCKTKYKRHK-GSPPVHGEENEDVDA-DDVSD
OsCESA2 29 FTACDVCGFPVCRPCYEYERKDGSQACPQCKTKYKRHK-GSPPILGDESDDVDA-DDASD
HvCESA3 34 FAACDVCGFPVCRPCYEYERKEGTQACPQCKTKYKRHK-GSPPARGDESE-----DDASD
TaCESA3B 50 FAACDVCAFPVCRPCYEYERKEGTQACPQCKTKYKRHK-GSPPARGDESE-----DDASD
TaCESA3D 50 FAACDVCAFPVCRPCYEYERKEGTQACPQCKTKYKRHK-GSPPARGDESE-----DDASD
TaCESA3A 1 ------------------------------------------------------------
HvCESA1 35 FTACDVCRFPVCRPCYEHERKEGTQACLQCKTKYKRHK-GSPVIRGEEGDDTDA-DDGSD
TaCESA1D 1 ----------------------------------MASS-GSPAIRGEEGDDTDA-DDGSD
TaCESA1A 35 FTACDVCRFPVCRPCYEHERKEGTQACLQCKTKYKRHR-GSPAIRGEEGDDTDA-DDGSD
TaCESA1B 35 FTACDVCRFPVCRPCYEHERKEGTQACLQCKTKYKRHR-GSPPIRGEEGDDTDA-DDGSD
OsCESA8 35 FAACDVCGFPVCRPCYEYERKDGTQACPQCKTKYKRHK-GSPAIRGEEGEDTDA-DDVSD
ZmCESA4 35 FAACDVCGFPVCRPCYEYERKDGTQACPQCKTKYKRHK-GSPAIRGEEGDDTDA-D--SD
ZmCESA9 35 FTACDVCGFPVCRPCYEYERKDGTQACPQCKNKYKRHK-GSPAIRGEEGDDTDA-DDASD
OsCESA10 1 ------------------------------------------------------------
OsCESA11 1 ------------------------------------------------------------
ZmCESA10 116 FPAKSPK-------------------------------------------------KPHE
OsCESA7 93 FQIKSPTKQK----------------------------------------------PPHE
TaCESA4D 89 FQVKSPK-------------------------------------------------KPHE
TaCESA4B 88 FQVKSPK-------------------------------------------------KPHE
TaCESA4A 88 FQVKSPK-------------------------------------------------KPHE
HvCESA4 85 FQVKSPK-------------------------------------------------KPHE
ZmCESA11 73 H-HTAGG--------------------LRERVTMGSHL----------------------
OsCESA4 72 E-PAAGG--------------------VRERVTMASQL----------------------
HvCESA7/5 75 Q-LAAEG--------------------LRGRVTMANQL----------------------
TaCESA7B 73 Q-LVAEG--------------------LRGRVTMANQL----------------------
TaCESA7D 1 ---------------------------------MLDVI----------------------
TaCESA8D 85 FNIDDDKQLQ---QHGALQNSHITEAMLHGRMSYGRASEDGGEG----------NNTP--
TaCESA8B 111 FNIDDDKQ-Q---QHGALQNSHITEAMLHGKMSYGRASEDGGEG----------NNTPM-
HvCESA8 111 FNIDDDKH-Q---QHAALHSTHITDAMLHGKMSYGRASEDGGDG----------NNTPMV
OsCESA9 111 FNIDDEKQKQLQQDQDGMQNSHITEAMLHGKMSYGRGPDDGD-G----------NST---
ZmCESA12 111 FNIDDENQQR--QLEGNMQNSQITEAMLHGRMSYGRGPDDGD-G----------NNT---
ZmCESA13 111 FNIDDEK-QR--QLEGNMQNSQITEAMLHGKMSYGRGADDGE-G----------NNT---
OsCESA6 116 FGLDG---RE-------DDPQYIAESMLRANMSYGRGGD---------------LQP-FQ
ZmCESA8 115 FGLQDGAAHE-------DDPQYVAESMLRAQMSYGRGGD---------------AHPGFS
TaCESA2A 113 FNWR----DR-------DDSQYAAESMLHAHMTYGRGG------------DLDGVHQPFQ
TaCESA2B 113 FNWR----DR-------DDSQYAAESMLHAHMTYGRGG------------DLDGVHQPFQ
TaCESA2D 113 FNWR----DR-------DDSQYAAESMLHAHMTYGRGG------------DLDGVHQPFQ
HvCESA2 113 FNWR----DR-------DDSQYAAESMLHAHMTYGRGG------------DLDGVHQPFQ
ZmCESA7 113 FNWD----G--------HDSQSVAESMLYGHMSYGRGG------------DPNGAPQAFQ
ZmCESA6 83 FNWS----DK-------HDSQYLAESMLHAHMSYGRG-A-----------DLDGVPQPFH
OsCESA3 113 FNWR----DR-------NDSQYVAESMLHAHMSYGRGGV-----------DVNGVPQPFQ
OsCESA5 113 FNWR----DK-------TDSQYVAESMLHGHMSYGRGG------------DLDGVPQHFQ
TaCESA9B 1 ------------------------------------------------------------
ZmCESA3 112 FNYTQGNVQG-----------------PQWQLR-GQGEDVDIS-----------SSSRHE
ZmCESA1 115 FNYKQGSGKG-----------------PEWQLQ---GDDADLS-----------SSARHE
ZmCESA2 115 FNYKQGNGKG-----------------PEWQLQ---GDDADLS-----------SSARHD
OsCESA1 115 FNYKHGNGKG-----------------PEWQIQ-RQGEDVDLS-----------SSSRHE
TaCESA5A 115 FNYKQGNGKG-----------------PEWQLQ-GQGEDIDLS-----------SSSRHE
HvCESA6 115 FNYKQGNGKG-----------------PEWQ-----GEDIDLS-----------SSSCHD
TaCESA6B 109 FNYKQGNGKG-----------------PEWQ-----GEDIDLS-----------SSSRHE
TaCESA6A 115 FNYKQGNGKG-----------------PEWQ-----GEDIDLS-----------SSSRHE
TaCESA5D 115 FNYKQGNGKG-----------------PEWQLR-GQGEDIDLS-----------SSSRHE
TaCESA5B 115 FNYKQGNGKG-----------------PEWQLR-GQGEDIDLS-----------SSSRHE
HvCesA9 1 ------------------------------------------------------------
ZmCESA5 92 YNYQASGNQD--------QKQKIAERMLTWRTNSR-GSDIGLAKYDSGEIGHGKYDSGEI
OsCESA2 87 VNYPTSGNQD--------HKHKIAERMLTWRMNSGRNDDIVHSKYDSGEIGHPKYDSGEI
HvCESA3 88 FNYPASGNQD--------HKHRAPEKMLTWRRNSGASDDIGLTKFGSGEIGLHKYDSGEI
TaCESA3B 104 FNYPASANQD--------QKNKVPEKMLTWRRNSGASDDIGLTKFGSGEIGLHKYDSGEI
TaCESA3D 104 FNYPASANQD--------QKNKIPEKMLTWRRNSGASDDIGLTKFGSGEIGLHKYDSGEI
TaCESA3A 1 ------------------------------------------------------------
HvCESA1 93 FNYPASGTED--------QKQKIADRMRSWRMNTGGSGNVGHPKYDSGEIGLSKYDSGEI
TaCESA1D 25 FNYPASGTED--------QKQKIADRMRSWRMNTGGSGNVGHPKYDSGEIGLSKYDSGEI
TaCESA1A 93 FNYPASGTED--------QKQKIADRMRSWRMNTGGSGNVGHPKYDSGEIGLSKYDSGEI
TaCESA1B 93 FNYPASGTED--------QKQKIADRMRSWRMNTGGSGNVGHPKYDSGEIGLSKYDSGEI
OsCESA8 93 YNYPASGSAD--------QKQKIADRMRSWRMNAGGGGDVGRPKYDSGEIGLTKYDSGEI
ZmCESA4 91 FNYLASGNED--------QKQKIADRMRSWRMNVGGSGDVGRPKYDSGEIGLTKYDSGEI
ZmCESA9 93 FNYPASGNDD--------QKQKIADRMRSWRMNAGGSGDVGRPKYDSGEIGLTKYDSGEI
OsCESA10 1 ------------------------------------------------------------
OsCESA11 1 ------------------------------------------------------------
ZmCESA10 127 PVAFDVYSE-------NGEH----PAQKWRTGGQTLSSFTGSV----A------------
OsCESA7 107 PVNFDVYSE-------NGEQ----PAQKWRPGGPALSSFTGSV----A------------
TaCESA4D 100 PVPFDVYSE-------NGEQ----PPQKWRSGGPAMSSFGGSV----A------------
TaCESA4B 99 PVPFDVYSE-------NGEQ----PPQKWRSGGPAMSSFGGSV----A------------
TaCESA4A 99 PVPFDVYSE-------NGEQ----PPQKWRPGGPAMSSFGGSV----A------------
HvCESA4 96 PVPFDVYSE-------NGEQ----PPQKWRPGGPAMSSFGGSV----A------------
ZmCESA11 90 -------------NDRQDEVSHA----------RTMSSLSGIG-----------------
OsCESA4 89 -------------SDHQDEGVHARTMSTH---ARTISSVSGVG-----------------
HvCESA7/5 92 -------------SDRQDEVS-------H---ARTLSSMSGVG-----------------
TaCESA7B 90 -------------SDRQDVVS-------H---ARTLSSMSGIG-----------------
TaCESA7D 6 -------------LVQQDVVS-------H---ARTLSSMSGVG-----------------
TaCESA8D 130 ---------------VSGEFPMS-AGYGH---GDF------SSSMHKRIHPYPMSEPGS-
TaCESA8B 156 -VGIPPIITGNRSMPVSGEFPMS-AGHGH---GDF------SSSLHKRIHPYPMSEPGS-
HvCESA8 157 TVGIPPIITGNRSMPVSGEFPMS-AGHGH---GDF------SSSLHKRIHPYPMSEPGS-
OsCESA9 157 -P-LPPIITGARSVPVSGEFPIS-NSHGH---GEF------SSSLHKRIHPYPVSEPGS-
ZmCESA12 155 -PQIPPIITGSRSVPVSGEFPIT-NGYGH---GEV------SSSLHKRIHPYPVSEPGS-
ZmCESA13 154 -PQMPPIITGARSVPVSGEFPIT-NGYGH---GEL------SSSLHKRIHPYPVSEPGS-
OsCESA6 150 PIPNVPLLTNG---QMVDDI----PPEQH---ALVPSYMGGGGGGGKRIHPLPFADPSVP
ZmCESA8 153 PVPNVPLLTNG---QMVDDI----PPEQH---ALVPSYMSGGGGGGKRIHPLPFADPNLP
TaCESA2A 150 PNPNVPLLTNG---QMVDDI----PPEQH---ALVPSFVGG---GGKRIHPLPYADSNLP
TaCESA2B 150 PNPNVPLLTNG---QMVDDI----PPEQH---ALVPSFVGG---GGKRIHPLPYADSNLP
TaCESA2D 150 PNPNVPLLTNG---QMVDDI----PPEQH---ALVPSFVGG---GGKRIHPLPYADSNLP
HvCESA2 150 PNPNVPLLTNG---QMVDDI----PPEQH---ALVPSFVGG---GGKRIHPLPYADSNLP
ZmCESA7 149 LNPNVPLLTNG---QMVDDI----PPEQH---ALVPSFMGG---GGKRIHPLPYADPSLP
ZmCESA6 120 PIPNVPLLTNG---QMVDDI----PPDQH---ALVPSFVGG---GGKRIHPLPYADPNLP
OsCESA3 151 PNPNVPLLTDG---QMVDDI----PPEQH---ALVPSFMGG---GGKRIHPLPYADPNLP
OsCESA5 150 PIPNVPLLTNG---EMADDI----PPEQH---ALVPSFMGG---GGKRIHPLPYADPNLP
TaCESA9B 1 ------------------------------------------------------------
ZmCESA3 143 PHHRIPRLTTG--QQMSGDIPDA-SPDRH---SIRSPTPS-YVDPSIP-VPV-------R
ZmCESA1 144 PHHRIPRLTSG--QQISGEIPDA-SPDRH---SIRSPTSS-YVDPSVP-VPV-------R
ZmCESA2 144 PHHRIPRLTSG--QQISGEIPDA-SPDRH---SIRSPTSS-YVDPSVP-VPV-------R
OsCESA1 146 Q-HRIPRLTSG--QQISGEIPDA-SPDRH---SIRSGTSS-YVDPSVP-VPV-------R
TaCESA5A 146 PHHRIPRLTSG--QQISGEIPDA-SPDRH---SIRSPTSS-YVDPSVPGIPV-------R
HvCESA6 142 PHHRIPRLTTR--QQISGEIPDA-SPDRH---SIRSPTSS-YVDPSVP-VPV-------R
TaCESA6B 136 PHHRIPRLTSG--QQMSGEIPDA-SPDRH---SIRSPTSS-YVDPSVP-VPV-------R
TaCESA6A 142 PHHRIPRLTSG--QQMSGEIPDA-SPDRH---SIRSPTSS-YVDPSVP-VPV-------R
TaCESA5D 146 PHHRIPRLTSG--QQISGEIPDA-SPDRH---SIRSPTSS-YVDPSVP-VPV-------R
TaCESA5B 146 PHHRIPRLTSG--QQISGEIPDA-SPDRH---SIRSPTSS-YVDPSVP-VPV-------R
HvCesA9 1 ------------------------------------------------------------
ZmCESA5 143 PRGYIPSLT-H--SQISGEIPGA-SPDH----MMSPVGN--IG---RRGHQF-------P
OsCESA2 139 PRIYIPSLT-H--SQISGEIPGA-SPDH----MMSPVGN--IG---RRGHPF-------P
HvCESA3 140 PHGYIPRFS-H--SQASGEIPGA-SPDH----MMSPAGN--VG---KRGHPF-------A
TaCESA3B 156 PHGYIPRFS-H--SQVSGEISGA-SPDH----MLSPAGN--VG---KRGHPF-------A
TaCESA3D 156 PHGYIPRFS-H--SQVSGEISGA-SPDH----MMSPAGN--AG---KRGHPF-------A
TaCESA3A 1 --------------------------------MMSPAGN--VG---KRGHPF-------A
HvCESA1 145 PRGYVPSVT-N--SQMSGEIPGA-SPDHH---MMSPTGN--IS---RR-APF-------P
TaCESA1D 77 PRGYVPSVT-N--SQMSGEIPGA-SPDHH---MMSPTGN--IS---RR-APF-------P
TaCESA1A 145 PRGYVPSVT-N--SQMSGEIPGA-SPDHH---MMSPTGN--IS---RR-APF-------P
TaCESA1B 145 PRGYVPSVT-N--SQMSGEIPGA-SPDHH---MMSPTGN--IS---RR-APF-------P
OsCESA8 145 PRGYIPSVT-N--SQISGEIPGA-SPDHH---MMSPTGN--IG---KR-APF-------P
ZmCESA4 143 PRGYIPSVT-N--SQISGEIPGA-SPDHH---MMSPTGN--IG---KR-APF-------P
ZmCESA9 145 PRGYIPSVT-N--SQISGEIPGA-SPDHH---MMSPTGN--IG---RR-APF-------P
OsCESA10 1 ------------------------------------------------------------
OsCESA11 1 -------------------------MD-------------GESPEIMPVEC-------PD
ZmCESA10 160 --GKDL----EAEREMEGSMEWKDRIDKWKTKQEKRGKLNHDDSDD-------------D
OsCESA7 140 --GKDL----EQEREMEGGMEWKDRIDKWKTKQEKRGKLNRDDSDD-------------D
TaCESA4D 133 --GKEL----EAEREMEGSMEWKERIDKWKTKQEKRGKLNRDNSDD------------DD
TaCESA4B 132 --GKEL----EAEREMEGSMEWKERIDKWKTKQEKRGKLNRDNSDD------------DD
TaCESA4A 132 --GKEL----EAEREMEGSMEWKERIDKWKTKQEKRGKLNRDNSDD------------DD
HvCESA4 129 --GKEL----EAEREMEGSMEWKDRIDKWKTKQEKRGKLNRDNSDD------------DD
ZmCESA11 110 ----------SELNDESGKPIWKNRVESWKEKKNEKKASAKKTAAKA--------QPPP-
OsCESA4 116 ----------SELNDESGKPIWKNRVESWKEKKKEKKASAKKAAAKA--------QAPP-
HvCESA7/5 112 ----------SELNDESGKPIWKNRVDSWKEKKNEKKASAKKAAAKA--------QVPP-
TaCESA7B 110 ----------SELNDESGKPIWKNRVDSWKEKKNEKKASAKKAAAKA--------QVPP-
TaCESA7D 26 ----------SELNDESGKPIWKNRVDSWKEKKNEKKASVKKAAAKA--------QVPP-
TaCESA8D 164 ----------AKWGDEKKEVSWKERMDDWKSKQGIY----------------GAAD----
TaCESA8B 204 ----------AKWGDEKKEVSWKERMDDWKSKQGIY----------------GAAD----
HvCESA8 206 ----------AKWGDEKKEVSWKERMDDWKSKQGIY----------------GAAD----
OsCESA9 204 ----------AKWD-EKKEVSWKERMDDWKSKQGIVA--------------GGAPD----
ZmCESA12 203 ----------AKWD-EKKEVSWKERMDDWKSKQGILG--------------G-GAD----
ZmCESA13 202 ----------AKWD-EKKEVSWKERMDDWKSKQGILG--------------GGGGD----
OsCESA6 200 VQPRSMDPSKDLAAYGYGSVAWKERMEGWKQKQERMQQLRS---------EGG-GDWDG-
ZmCESA8 203 VQPRSMDPSKDLAAYGYGSVAWKERMEGWKQKQERLQHVRS---------EGG-GDWDG-
TaCESA2A 197 VQPRSMDPSKDIGSYGYGSVAWKERMESWKQKQERLHQARN---------DG-GKDWNG-
TaCESA2B 197 VQPRSMDPSKDIGSYGYGSVAWKERMESWKQKQERLHQARN---------DG-GKDWNG-
TaCESA2D 197 VQPRSMDPSKDIGSYGYGSVAWKERMESWKQKQERLHQARN---------DG-GKDWNG-
HvCESA2 197 VQPRSMDPSKDIGSYGYGSVAWKERMESWKQKQERLHQTRN---------DG-GKDWNG-
ZmCESA7 196 VQPRSMDPSKDLAAYGYGSVAWKERMENWKQRQERMHQTGN---------DGGGD-----
ZmCESA6 167 VQPRSMDPSKDLAAYGYGSVAWKERMESWKQKQERMHQTRN---------DGGGD-----
OsCESA3 198 VQPRSMDPSKDLAAYGYGSVAWKERMESWKQKQERLHQMRN---------DGGGKDWDG-
OsCESA5 197 VQPRSMDPSKDLAAYGYGSVAWKERMESWKQKQERLHQMRN---------DGGGKDWDG-
TaCESA9B 1 ------------------------------------------------------------
ZmCESA3 188 IV----DPSKDLNSYGVGSVDWKERVESWKVRQDKNMIQVTHKYPA---E--GKGDIEG-
ZmCESA1 189 IV----DPSKDLNSYGLNSVDWKERVESWRVKQDKNMMQVTNKYPE---AR--GGDMEG-
ZmCESA2 189 IV----DPSKDLNSYGLNSVDWKERVESWRVKQDKNMLQVTNKYPE---AR---GDMEG-
OsCESA1 190 IV----DPSKDLNSYGINSVDWQERVASWRNKQDKNMMQVANKYPE---AR--GGDMEG-
TaCESA5A 192 IV----DPSKDLNSYGLNSVDWKERVESWRVKQDKNMMQVTNKYPD---ARG-GGDMEG-
HvCESA6 187 IV----DPSKDLNSYGLNSVDWKERVESRRVKQDKNMMQVTNKYPD---PRG-GGDMEG-
TaCESA6B 181 IV----DPSKDLNSYGLNSVDWKERVESWRVKQDKNMMQVTNKYPD---ARGGGGDMEG-
TaCESA6A 187 IV----DPSKDLNSYGLNSVDWKERVESWRVKQDKNMMQVTNKYPD---ARGGGGDMEG-
TaCESA5D 191 IV----DPSKDLNSYGLNSVDWKERVESWRVKQDKNMMQVTNKYPD---ARGGGGDMEG-
TaCESA5B 191 IV----DPSKDLNSYGLNSVDWKERVESWRVKQDKNMMQVTNKYPD---ARG-GGDMEG-
HvCesA9 1 ------------------------------------------------------------
ZmCESA5 183 YVNHSPNPSRE-FSGSLGNVAWKERVDGWKMK-DKGAIPMTNGTSIAPSEGRGVADIDAS
OsCESA2 179 YVNHSPNPSRE-FSGSLGNVAWKERVDGWKMK-DKGAIPMANGTSIAPSEGRGVGDIDAS
HvCESA3 180 YVNHSPNPSRE-FSGSLGNVAWKERVDGWKMK-DKGAIPMTNGTSIAPSEGRGNGDIDAC
TaCESA3B 196 YVNHSSNPSRE-FSGSLGNVAWKERVDGWKMK-DKGAIPMTNGTSIAPSEGRGNGDIDAC
TaCESA3D 196 YVNHSPNPSRE-FSGSLGNVAWKERVDGWKMK-DKGAIPMTNGTSIAPSEGRGNGDIDAC
TaCESA3A 17 YVNHSPNPSRE-FSGSLGNVAWKERVDGWKMK-DKGAIPMTNGTSIAPSEGRGNGDIDAC
HvCESA1 185 YVNHSPNPSRE-FSGSIGNVAWKERVDGWKMKQDKGAIPMTNGTSIAPSEGRAATDIDAS
TaCESA1D 117 YVNHSPNPSRE-FSGSIGNVAWKERVDGWKMKQDKGAIPMTNGTSIAPSEGRAATDIDAS
TaCESA1A 185 YVNHSPNPSRE-FSGSIGNVAWKERVDGWKMKQDKGAIPMTNGTSIAPSEGRAATDIDAS
TaCESA1B 185 YVNHSPNPSRE-FSGSIGNVAWKERVDGWKMKQDKGAIPMTNGTSIAPSEGRAATDIDAS
OsCESA8 185 YVNHSPNPSRE-FSGSIGNVAWKERVDGWKLKQDKGAIPMTNGTSIAPSEGRGVGDIDAS
ZmCESA4 183 YVNHSPNPSRE-FSGSIGNVAWKERVDGWKMKQDKGTIPMTNGTSIAPSEGRGVGDIDAS
ZmCESA9 185 YMNHSSNPSRE-FSGSVGNVAWKERVDGWKMKQDKGTIPMTNGTSIAPSEGRGVGDIDAS
OsCESA10 1 ------------------------------------------------------------
OsCESA11 16 PEPASSESGDDHDIPEPLSSRLSVPSGELNLYRAAVALRLVLLAAFFRYRVTRPVADAHA
ZmCESA10 201 DDKNEDEYMLLAEARQPLWRKVPIPSSMINPYRIVIVLRLVVLCFFLKFRITTPATDAVP
OsCESA7 181 DDKNDDEYMLLAEARQPLWRKVPIPSSKINPYRIVIVLRLVVLCFFLKFRITTPAMDAVP
TaCESA4D 175 DDD---------------------------------------------------------
TaCESA4B 174 DDKNDDEYMLRXXXXQPLWRKLPVPSSQINPYRIVIVLRLVVLCFFLRFRIMTPANDAIP
TaCESA4A 174 DDKNDDEYMLLAEARQPLWRKLPVPSSQINPYRIVIVLRLVVLCFFLRFRIMTPANDAIP
HvCESA4 171 DDKNDDEYMLLAEARQPLWRKLPVPSSQINPYRIVIVLRLVVLCFFLRFRIMTPANDAIP
ZmCESA11 151 VEEQIMDEKDLTDAYEPLSRVIPISKNKLTPYRAVIIMRLIVLGLFFHYRITNPVNSAFG
OsCESA4 157 VEEQIMDEKDLTDAYEPLSRIIPISKNKLTPYRAVIIMRLVVLGLFFHYRITNPVYSAFG
HvCESA7/5 153 VEEQIMEEKDLTDAYEPLSRIIPISKNKLTPYRAVIIMRLVVLGLFFHYRITNPVDSAFG
TaCESA7B 151 VEEQIMEEKDLTDAYEPLSRIIPISKNKLTPYRAVIIMRLVVLGLFFHYRITNPVDSAFG
TaCESA7D 67 VEEQIMEEKDLTDAYEPLSRIIPISKNKLTPYRAVIIMRLVVLGLFFHYRITNPVDSAFG
TaCESA8D 194 PDDMDADVPLNDEAR---------------------------------------------
TaCESA8B 234 PDDMDADVPLNDEARQPLSRKVSIASSKVNPYRMVIILRLFVLCVFLRYRILNPVPEAIP
HvCESA8 236 PDDMDADVPLNDEARQPLSRKVSIASSKVNPYRMVIILRLFVLCVFLRYRILNPVPEAIP
OsCESA9 235 PDDYDADVPLNDEARQPLSRKVSIASSKVNPYRMVIILRLVVLGFFLRYRILHPVPDAIP
ZmCESA12 233 PEDMDADVALNDEARQPLSRKVSIASSKVNPYRMVIVVRLVVLAFFLRYRILHPVPDAIG
ZmCESA13 233 PEDMDADVPLNDEARQPLSRKVSIASSKVNPYRMVIVVRLVVLAFFLRYRILHPVPDAIG
OsCESA6 249 -DGD-ADLPLMDEARQPLSRKVPISSSRINPYRMIIIIRLVVLGFFFHYRVMHPVNDAFA
ZmCESA8 252 -D-D-ADLPLMDEARQPLSRKVPISSSRINPYRMIIVIRLVVLGFFFHYRVMHPAKDAFA
TaCESA2A 246 -DGDDADLPLMDEARQPLSRKVPIPSSLINPYRMIIVIRLVIVCLFFHYRVMHPVHDAFV
TaCESA2B 246 -DGDDADLPLMDEARQPLSRKVPIPSSLINPYRMIIVIRLVIVCLFFHYRVMHPVHDAFV
TaCESA2D 246 -DGDDADLPLMDEARQPLSRKVPIPSSLINPYRMIIVIRLVIVCLFFHYRVMHPVHDAFV
HvCESA2 246 -DGDDADLPLMDEARQPLSRKVPIPSSLINPYRMIIVIRLVIVCLFFHYRVMHPVHDAFV
ZmCESA7 242 -DGDDADLPLMDEARQQLSRKIPLPSSQINPYRMIIIIRLVVLGFFFHYRVMHPVNDAFA
ZmCESA6 213 -DGDDADLPLMDEARQPLSRKIPLPSSQINPYRMIIIIRLVVLCFFFHYRVMHPVPDAFA
OsCESA3 248 -DGDDGDLPLMDEARQPLSRKVPIPSSQINPYRMVIIIRLVVLGFFFHYRVMHPVPDAFA
OsCESA5 247 -DGDDADLPLMDEARQPLSRKIPISSSLVNPYRMIIIIRLVVLGFFFHYRVMHPVPDAFA
TaCESA9B 1 ------------------------------------------------------------
ZmCESA3 238 TGSNGEDLQMADDARLPLSRIVPISPNELNLYRIVIVLRLIILCFFFQYRITHPVEDAYG
ZmCESA1 239 TGSNGEXMQMVDDARLPLSRIVPISSNQLNLYRVVIILRLIILCFFFQYRVSHPVRDAYG
ZmCESA2 238 TGSNGEDMQMVDDARLPLSRIVPISSNQLNLYRIVIILRLIILCFFFQYRISHPVRNAYG
OsCESA1 240 TGSNGEDMQMVDDARLPLSRIVPIPSNQLNLYRIVIILRLIILMFFFQYRVTHPVRDAYG
TaCESA5A 243 TGSNGEDMQMVDDARLPLSRIVPIPANQLNLYRIVIILRLIILCFFFQYRVTHPVRDAYG
HvCESA6 238 TGSNGEDMQMVDDARLPLSRIVPIPANQLNLYRIVIILRLIILCFFFQYRVSHPVRDAYG
TaCESA6B 233 TGSNGEDMQMVDDARLPLSRIVPIPANQLNLYRIVIILRLIILCFFFQYRVSHPVRDAYG
TaCESA6A 239 TGSNGEDMQMVDDARLPLSRIVPIPANQLNLYRIVIILRLIILCFFFQYRVSHPVRDAYG
TaCESA5D 243 TGSNGEDMQMVDDARLPLSRIVPIPANQLNLYRIVIILRLIILCFFFQYRVTHPVRDAYG
TaCESA5B 242 TGSNGEDMQMVDDARLPLSRIVPIPANQLNLYRIVIILRLIILCFFFQYRVTHPVRDAYG
HvCesA9 1 ------------------------------------------------------------
ZmCESA5 241 TDYNMEDALLNDETRQPLSRKVPIPSSRINPYRMVIVLRLAVLCIFLRYRITHPVNNAYP
OsCESA2 237 TDYNMEDALLNDETRQPLSRKVPISSSRINPYRMVIVLRLIVLCIFLHYRITNPVRNAYP
HvCESA3 238 TDYGMEDPLLNDETRQPLSRKVPIPSSRINPYRMVIVLRLIVLCIFLHYRITNPVRNAYP
TaCESA3B 254 TDYGMEDPLLNDETRQPLSRKVPIPSSRINPYRMVIVLRLIVLCIFLHYRITNPVRNAYP
TaCESA3D 254 TDYGMEDPLLNDETRQPLSRKVPIPSSRINPYRMVIVLRLIVLCIFLHYRITNPVRNAYP
TaCESA3A 75 TDYGMEDPLLNDETRQPLSRKVPIPSSRINPYRMVIVLRLIVLCIFLHYRITNPVRNAYP
HvCESA1 244 TEYNMEDALLNDETRQPLSRKVPIASSKINPYRMVIVLRLVVLSIFLHYRLTNPVRNAYP
TaCESA1D 176 TEYNMEDALLNDETRQPLSRKVPIASSKINPYRMVIVLRLVVLSIFLHYRLTNPVRNAYP
TaCESA1A 244 TEYNMEDALLNDETRQPLSRKVPIASSKINPYRMVIVLRLVVLSIFLHYRLTNPVRNAYP
TaCESA1B 244 TEYNMEDALLNDETRQPLSRKVPIASSKINPYRMVIVLRLVVLSIFLHYRLTNPVRNAYP
OsCESA8 244 TDYNMEDALLNDETRQPLSRKVPLPSSRINPYRMVIVLRLVVLSIFLHYRITNPVRNAYP
ZmCESA4 242 TDYNMEDALLNDETRQPLSRKVPLPSSRINPYRMVIVLRLIVLSIFLHYRITNPVRNAYP
ZmCESA9 244 TDYNMEDALLNDETRQPLSRKVPLPSSRINPYRMVIVLRLIVLSIFLHYRITNPVRNAYP
OsCESA10 1 --------------------------------MDVFVTTADPDGIAALDDDALLPAMDVF
OsCESA11 76 LWVTSVACELWLAASWLIAQLPKLSPANRVTYLDRLASRYEKG-----GEASRLAGVDVF
ZmCESA10 261 LWLASVICELWFAFSWILDQLPKWAPVTRETYLDRLALRYDRE-----GEACRLSPIDFF
OsCESA7 241 LWLASVICELWFALSWILDQLPKWSPVTRETYLDRLALRYERD-----GEPCRLAPIDFF
TaCESA4D 178 ------------------------------------------------------------
TaCESA4B 234 LWLVSVICELWFALSWILDQLPKWSPVTRETYLDRLALRYDRE-----GEPSRLSPIDFF
TaCESA4A 234 LWLVSVICELWFALSWILDQLPKWSPVTRETYLDRLALRYDRE-----GEPSRLSPIDFF
HvCESA4 231 LWLVSVICELWFALSWILDQLPKWSPVTRETYLDRLALRYDRE-----GEPSRLSPIDFF
ZmCESA11 211 LWMTSVICEIWFGFSWILDQFPKWYPINRETYVDRLIARYG-D-----GEESGLAPVDFF
OsCESA4 217 LWMTSVICEIWFGFSWILDQFPKWCPINRETYVDRLIARYG-D-----GEDSGLAPVDFF
HvCESA7/5 213 LWLTSVICEIWFGFSWILDQFPKWCPVNRETYVDRLIARYG-D-----GEDSGLAPVDFF
TaCESA7B 211 LWLTSVICEIWFGFSWILDQFPKWCPVNRETYVDRLIARYG-D-----GEDSGLAPVDFF
TaCESA7D 127 LWLTSVICEIWFGFSWILDQFPKWCPVNRETYVDRLIARYG-D-----GEDSGLAPVDFF
TaCESA8D 209 ------------------------------------------------------------
TaCESA8B 294 LWLTSIVCEIWFAVSWILDQFPKWYPIDRETYLDRLSLRYERE-----GEPSMLSPVDLF
HvCESA8 296 LWLTSIVCEIWFAVSWILDQFPKWYPIDRETYLDRLSLRYERE-----GEPSMLSPVDLF
OsCESA9 295 LWLTSIICEIWFAVSWILDQFPKWYPIDRETYLDRLSLRYERE-----GEPSLLSAVDLF
ZmCESA12 293 LWLVSIICEIWFAISWILDQFPKWFPIDRETYLDRLSLRYERE-----GEPSLLSAVDLF
ZmCESA13 293 LWLVSIICEIWFAVSWILDQFPKWFPIDRETYLDRLTLRYERE-----GEPSLLSSVDLF
OsCESA6 307 LWLISVICEIWFAMSWILDQFPKWLPIERETYLDRLSLRFDKE-----GQPSQLAPVDFF
ZmCESA8 309 LWLISVICEIWFAMSWILDQFPKWLPIERETYLDRLSLRFDKE-----GQPSQLAPIDFF
TaCESA2A 305 LWLISVICEIWFAMSWILDQFPKWFPIERETYLDRLTLRFDKE-----GQPSQLAPVDFF
TaCESA2B 305 LWLISVICEIWFAMSWILDQFPKWFPIERETYLDRLTLRFDKE-----GQPSQLAPVDFF
TaCESA2D 305 LWLISVICEIWFAMSWILDQFPKWFPIERETYLDRLTLRFDKE-----GQPSQLAPVDFF
HvCESA2 305 LWLISVICEIWFAMSWILDQFPKWFPIERETYLDRLTLRFDKE-----GQPSQLAPVDFF
ZmCESA7 301 LWLISVICEIWFAMSWILDQFPKWFPIERETYLDRLSLRFDKE-----GQPSQLAPIDFF
ZmCESA6 272 LWLISVICEIWFAMSWILDQFPKWFPIERETYLDRLSLRFDKE-----GHPSQLAPVDFF
OsCESA3 307 LWLISVICEIWFAMSWILDQFPKWFPIERETYLDRLTLRFDKE-----GQTSQLAPIDFF
OsCESA5 306 LWLISVICEIWFAMSWILDQFPKWFPIERETYLDRLTLRFDKE-----GQQSQLAPVDFF
TaCESA9B 1 ------------------------------------------------------------
ZmCESA3 298 LWLVSVICEVWFALSWLLDQFPKWYPINRETYLDRLALRYDRE-----GEPSQLAPIDVF
ZmCESA1 299 LWLVSVICEVWFALSWLLDQFPKWYPINRETYLDRLALRYDRE-----GEPSQLAPIDVF
ZmCESA2 298 LWLVSVICEVWFALSWLLDQFPKWYPINRETYLDRLALRYDRE-----GEPSQLAPIDVF
OsCESA1 300 LWLVSVICEIWFALSWLLDQFPKWYPINRETYLDRLALRYDRE-----GEPSQLAPIDVF
TaCESA5A 303 LWLVSVICEIWFALSWLLDQFPKWYPINRETYLDRLALRYDRE-----GEPSQLCPIDIF
HvCESA6 298 LWLVSVICEIWFALSWLLDQFPKWYPINRETYLDRLALRYDRE-----GEPSQLCPIDIF
TaCESA6B 293 LWLVSVICEIWFALSWLLDQFPKWYPINRETYLDRLALRYDRE-----GEPSQLCPIDIF
TaCESA6A 299 LWLVSVICEIWFALSWLLDQFPKWYPINRETYLDRLALRYDRE-----GEPSQLCPIDIF
TaCESA5D 303 LWLVSVICEIWFALSWLLDQFPKWYPINRETYLDRLALRYDRE-----GEPSQLCPIDIF
TaCESA5B 302 LWLVSVICEIWFALSWLLDQFPKWYPINRETYLDRLALRYDRE-----GEPSQLCPIDIF
HvCesA9 1 ------------------------------------------------------------
ZmCESA5 301 LWLLSVICEIWFALSWILDQFPKWSPINRETYLDRLALRYDRE-----GEPSQLAPVDIF
OsCESA2 297 LWLLSVICEIWFALSWILDQFPKWSPINRETYLDRLALRYDRE-----GEPSQLAPVDIF
HvCESA3 298 LWLLSVICEIWFAFSWILDQFPKWSPVNRETYLDRLALRYDRD-----GELSQLAPVDIF
TaCESA3B 314 LWLLSVICEIWFAFSWILDQFPKWSPVNRETYLDRLALRYDRD-----GELSQLAPVDIF
TaCESA3D 314 LWLLSVICEIWFAFSWILDQFPKWSPVNRETYLDRLALRYDRD-----GELSQLAPVDIF
TaCESA3A 135 LWLLSVICEIWFAFSWILDQFPKWSPVNRETYLDRLALRYDRD-----GELSQLAPVDIF
HvCESA1 304 LWLLSVICEIWFALSWILDQFPKWFPINRETYLDRLALRYDRE-----GEPSQLAAVDIF
TaCESA1D 236 LWLLSVICEIWFALSWILDQFPKWFPINRETYLDRLALRYDRE-----GEPSQLAAVDIF
TaCESA1A 304 LWLLSVICEIWFALSWILDQFPKWFPINRETYLDRLALRYDRE-----GEPSQLAAVDIF
TaCESA1B 304 LWLLSVICEIWFALSWILDQFPKWFPINRETYLDRLALRYDRE-----GEPSQLAAVDIF
OsCESA8 304 LWLLSVICEIWFALSWILDQFPKWFPINRETYLDRLALRYDRE-----GEPSQLAAVDIF
ZmCESA4 302 LWLLSVICEIWFALSWILDQFPKWFPINRETYLDRLALRYDRE-----GEPSQLAAVDIF
ZmCESA9 304 LWLLSVICEIWFALSWILDQFPKWFPINRETYLDRLALRYDRE-----GEPSQLAAVDIF
OsCESA10 29 VTTADPDKEPPLATANTVLSIYPRRGLPRRQ-----------------------------
OsCESA11 131 VAAADAAREPPLATANTVLSVLAADYPAGGVACYVHDDGADMLVFESLFEAAGFARRWIP
ZmCESA10 316 VSTVDPLKEPPIITANTVLSILAVDYPVDRVSCYVSDDGASMLLFDALSETAEFARRWVP
OsCESA7 296 VSTVDPLKEPPIITANTVLSILAVDYPVDRVSCYVSDDGASMLLFDTLSETAEFARRWVP
TaCESA4D 178 ------------------------------------------------------------
TaCESA4B 289 VSTVDPLKEPPIITANTVLSILAVDYPVDRNSCYVSDDGASMLCFDTLSETAEFARRWVP
TaCESA4A 289 VSTVDPLKEPPIITANTVLSILAVDYPVDRNSCYVSDDGASMLCFDTLSETAEFARRWVP
HvCESA4 286 VSTVDPLKEPPIITANTVLSILAVDYPVDRNSCYVSDDGASMLCFDTLSETAEFARRWVP
ZmCESA11 265 VSTVDPLKEPPLITANTVLSILAVDYPVEKISCYVSDDGSAMLTFESLAETAEYARKWVP
OsCESA4 271 VSTVDPLKEPPLITANTVLSILAVDYPVEKISCYVSDDGSAMLTFESLAETAEFARRWVP
HvCESA7/5 267 VSTVDPLKEPPLITANTVLSILAVDYPVEKISCYVSDDGSAMLTFESLAETAEFARRWVP
TaCESA7B 265 VSTVDPLKEPPLITANTVLSILAVDYPVEKISCYVSDDGAAMLTFESLAETAEFARRWVP
TaCESA7D 181 VSTVDPLKEPPLITANTVLSILAVDYPVEKISCYVSDDGAAMLTFESLAETAEFARRWVP
TaCESA8D 209 -------KEPPLVTANTVLSILAVDYPVDKVSCYVSDDGAAMLTFESLSETAEFARKWVP
TaCESA8B 349 VSTVDPLKEPPLVTANTVLSILAVDYPVDKVSCYVSDDGASMLSFESLSETAEFARKWVP
HvCESA8 351 VSTVDPLKEPPLVTANTVLSILAVDYPVDKVSCYVSDDGASMLSFESLSETAEFARKWVP
OsCESA9 350 VSTVDPLKEPPLVTANTVLSILAVDYPVDKVSCYVSDDGASMLTFESLSETAEFARKWVP
ZmCESA12 348 VSTVDPLKEPPLVTANTVLSILAVDYPVDKVSCYVSDDGASMLTFESLSETAEFARKWVP
ZmCESA13 348 VSTVDPLKEPPLVTANTVLSILAVDYPVDKVSCYVSDDGASMLTFEALSETAEFARKWVP
OsCESA6 362 VSTVDPSKEPPLVTANTVLSILSVDYPVEKVSCYVSDDGAAMLTFEALSETSEFAKKWVP
ZmCESA8 364 VSTVDPTKEPPLVTANTVLSILSVDYPVEKVSCYVSDDGAAMLTFEALSETSEFAKKWVP
TaCESA2A 360 VSTVDPAKEPPLVTANTILSILAVDYPVDKLSCYVSDDGAAMLTFEGLSETSEFAKKWVP
TaCESA2B 360 VSTVDPAKEPPLVTANTILSILAVDYPVDKLSCYVSDDGAAMLTFEGLSETSEFAKKWVP
TaCESA2D 360 VSTVDPAKEPPLVTANTILSILAVDYPVDKLSCYVSDDGAAMLTFEGLSETSEFAKKWVP
HvCESA2 360 VSTVDPAKEPPLVTANTILSILAVDYPVDKLSCYVSDDGAAMLTFEGLSETSEFAKKWVP
ZmCESA7 356 VSTVDPLKEPPLVTTNTVLSILSVDYPVDKVSCYVSDDGAAMLTFEALSETSEFAKKWVP
ZmCESA6 327 VSTVDPLKEPPLVTANTVLSILSVDYPVDKVSCYVSDDGAAMLTFEALSETSEFAKKWVP
OsCESA3 362 VSTVDPLKEPPLVTANTVLSILAVDYPVDKVSCYVSDDGAAMLTFEALSETSEFAKKWVP
OsCESA5 361 VSTVDPMKEPPLVTANTVLSILAVDYPVDKVSCYVSDDGAAMLTFEALSETSEFAKKWVP
TaCESA9B 1 -----------------------------------------MLTFXXXXXXXEFARKWVP
ZmCESA3 353 VSTVDPLKEPPLITGNTVLSILAVDYPVDKVSCYVSDDGSAMLTFEALSETAEFARKWVP
ZmCESA1 354 VSTVDPLKEPPLITANTVLSILSVDYPVDKVSCYVSDDGSAMLTFESLSETAEFARKWVP
ZmCESA2 353 VSTVDPLKEPPLITANTVLSILAVDYPVDKVSCYVSDDGSAMLTFESLSETAEFARKWVP
OsCESA1 355 VSTVDPLKEPPLITANTVLSILAVDYPVDKVSCYVSDDGSAMLTFEALSETAEFARKWVP
TaCESA5A 358 VSTVDPLKEPPLITANTVLSILAVDYPVDKVSCYVSDDGSAMLTFESLSETAEFARKWVP
HvCESA6 353 VSTVDPLKEPPLITANTVLSILAVDYPVDKVSCYVSDDGSAMLTFESLSETAEFARKWVP
TaCESA6B 348 VSTVDPLKEPPLITANTVLSILAVDYPVDKVSCYVSDDGSAMLTFESLSETAEFARKWVP
TaCESA6A 354 VSTVDPLKEPPLITANTVLSILAVDYPVDKVSCYVSDDGSAMLTFESLSETAEFARKWVP
TaCESA5D 358 VSTVDPLKEPPLITANTVLSILAVDYPVDKVSCYVSDDGSAMLTFESLSETAEFARKWVP
TaCESA5B 357 VSTVDPLKEPPLITANTVLSILAVDYPVDKVSCYVSDDGSAMLTFESLSETAEFARKWVP
HvCesA9 1 -----------------------------------------MLTFESLSETAEFARKWVP
ZmCESA5 356 VSTVDPMKEPPLVTANTVLSILAVDYPVDKVSCYVSDDGAAMLTFDALSETSEFARKWVP
OsCESA2 352 VSTVDPMKEPPLVTANTVLSILAVDYPVDKVSCYVSDDGAAMLTFDALAETSEFARKWVP
HvCESA3 353 VSTVDPMKEPPLVTANTVLSILAVDYPVDKVSCYVSDDGAAMLTFDALAETSEFARKWVP
TaCESA3B 369 VSTVDPMKEPPLVTANTVLSILAVDYPVDKVSCYVSDDGAAMLTFDALAETSEFARKWVP
TaCESA3D 369 VSTVDPMKEPPLVTANTVLSILAVDYPVDKVSCYVSDDGAAMLTFDALAETSEFARKWVP
TaCESA3A 190 VSTVDPMKEPPLVTANTVLSILAVDYPVDKVSCYVSDDGAAMLTFDALAETSEFARKWVP
HvCESA1 359 VSTVDPLKEPPIVTANTVLSILAVDYPVDKVSCYVSDDGASMLTFDALAETSEFARKWVP
TaCESA1D 291 VSTVDPLKEPPIVTANTVLSILAVDYPVDKVSCYVSDDGASMLTFDALAETSEFARKWVP
TaCESA1A 359 VSTVDPLKEPPIVTANTVLSILAVDYPVDKVSCYVSDDGASMLTFDALAETSEFARKWVP
TaCESA1B 359 VSTVDPLKEPPIVTANTVLSILAVDYPVDKVSCYVSDDGASMLTFDALAETSEFARKWVP
OsCESA8 359 VSTVDPMKEPPLVTANTVLSILAVDYPVDKVSCYVSDDGAAMLTFDALAETSEFARKWVP
ZmCESA4 357 VSTVDPMKEPPLVTANTVLSILAVDYPVDKVSCYVSDDGAAMLTFDALAETSEFARKWVP
ZmCESA9 359 VSTVDPMKEPPLVTANTVLSILAVDYPVDKVSCYVSDDGAAMLTFDALAETSEFARKWVP
OsCESA10 60 -------------------------------------------------VVQVLIDSAGS
OsCESA11 191 FCRRHGVEPRAPELYFARGVDYLRDRAAPSFVKDRRAM--KREYEEFKVRMNHLAARARK
ZmCESA10 376 FCKKFAVEPRAPEFYFSQKIDYLKDKVQPTFVKERRAM--KREYEEFKVRINALVAKAQK
OsCESA7 356 FCKKFTIEPRAPEFYFSQKIDYLKDKVQPTFVKERRAM--KREYEEFKVRINALVAKAQK
TaCESA4D 178 ------------------------------------------------------------
TaCESA4B 349 FCKKFAIEPRAPEFYFSQKIDYLKDKVQPTFVKERRAM--KREYEEFKVRINGLVAKAEK
TaCESA4A 349 FCKKFAIEPRAPEFYFSQKIDYLKDKVQPTFVKERRAM--KREYEEFKVRINGLVAKAEK
HvCESA4 346 FCKKFAIEPRAPEFYFSQKIDYLKDKVQPTFVKERRAM--KREYEEFKVRINGLVAKAEK
ZmCESA11 325 FCKKYAIEPRAPEFYFSQKIDYLKDKIHPSFVKERRAM--KRDYEEYKVRINALVAKAQK
OsCESA4 331 FCKKYSIEPRAPEFYFSQKIDYLKDKIHPSFVKERRAM--KRDYEEYKVRINALVAKAQK
HvCESA7/5 327 FCKKFSIEPRTPEFYFSQKIDYLKDKIHPSFVKERRAM--KRDYEEFKVRINALVAKAQK
TaCESA7B 325 FCKKFSIEPRTPEFYFSQKIDYLKDKIHPSFVKERRAM--KRDYEEFKVRINALVAKAQK
TaCESA7D 241 FCKKFSIEPRTPEFYFSQKIDYLKDKIHPSFVKERRAM--KRDYEEFKVRINALVAKAQK
TaCESA8D 262 FCKKFNIEPRAPEFYFSRKVDYLKDKVQPTFVQERRAMKMKREYEEFKVRINALVSKAQK
TaCESA8B 409 FCKKFNIEPRAPEFYFSRKVDYLKDKVQPTFVQERRAM--KREYEEFKVRINALVSKAQK
HvCESA8 411 FCKKFNIEPRAPEFYFSRKVDYLKDKVQPTFVQERRAM--KREYEEFKVRINALVSKAQK
OsCESA9 410 FCKKFSIEPRAPEFYFSQKVDYLKDKVHPNFVQERRAM--KREYEEFKVRINALVAKAQK
ZmCESA12 408 FCKKFGIEPRAPEFYFSLKVDYLKDKVQPTFVQERRAM--KREYEEFKVRINALVAKAMK
ZmCESA13 408 FCKKFCIEPRAPEFYFSLKVDYLKDKVQPTFVQERRAM--KREYEEFKVRINALVAKAMK
OsCESA6 422 FCKKFNIEPRAPEWYFQQKIDYLKDKVAASFVRERRAM--KRDYEEFKVRINALVAKAQK
ZmCESA8 424 FSKKFNIEPRAPEWYFQQKIDYLKDKVAASFVRERRAM--KREYEEFKVRINALVAKAQK
TaCESA2A 420 FCKKYSIEPRAPEWYFQQKIDYLKDKVVPNFVRDRRAM--KREYEEFKIRINALVAKAQK
TaCESA2B 420 FCKKYSIEPRAPEWYFQQKIDYLKDKVVPNFVRDRRAM--KREYEEFKIRINALVAKAQK
TaCESA2D 420 FCKKYSIEPRAPEWYFQQKIDYLKDKVVPNFVRDRRAM--KREYEEFKIRINALVAKAQK
HvCESA2 420 FCKKYSIEPRAPEWYFQQKIDYLKDKVVPNFVRDRRAM--KREYEEFKIRINALVAKAQK
ZmCESA7 416 FCKRYNIEPRAPEWYFQQKIDYLKDKVAANFVRERRAM--KREYEEFKVRINALVAKAQK
ZmCESA6 387 FCKRYSLEPRAPEWYFQQKIDYLKDKVAPNFVRERRAM--KREYEEFKVRINALVAKAQK
OsCESA3 422 FCKKYSIEPRAPEWYFQQKIDYLKDKVAPYFVRERRAM--KREYEEFKVRINALVAKAQK
OsCESA5 421 FCKRYSLEPRAPEWYFQQKIDYLKDKVAPNFVRERRAM--KREYEEFKVRINALVAKAQK
TaCESA9B 20 FVKKYDIEPRAPEFYFCQKIDYLKDKVQPSFVKDRRAM--KREYEEFKIRINALVSKALK
ZmCESA3 413 FCKKHNIEPRAPEFYFAQKIDYLKDKIQPSFVKERRAM--KREYEEFKVRINALVAKAQK
ZmCESA1 414 FCKKHNIEPRAPEFYFAQKIDYLKDKIQPSFVKERRAM--KREYEEFKVRINALVAKAQK
ZmCESA2 413 FCKKHNIEPRAPEFYFAQKIDYLKDKIQPSFVKERRAM--KREYEEFKIRINALVAKAQK
OsCESA1 415 FCKKHNIEPRAPEFYFAQKIDYLKDKIQPSFVKERRAM--KREYEEFKVRINALVAKAQK
TaCESA5A 418 FCKKHNIEPRAPEFYFQQKIDYLKDKIQPSFVKERRAM--KREYEEFKIRINALVAKAQK
HvCESA6 413 FCKKHNIEPRAPEFYFQQKIDYLKDKIQPSFVKERRAM--KREYEEFKIRINALVAKAQK
TaCESA6B 408 FCKKHNIEPRAPEFYFQQKIDYLKDKIQPSFVKERRAM--KREYEEFKIRINALVAKAQK
TaCESA6A 414 FCKKHNIEPRAPEFYFQQKIDYLKDKIQPSFVKERRAM--KREYEEFKIRINALVAKAQK
TaCESA5D 418 FCKKHNIEPRAPEFYFQQKIDYLKDKIQPSFVKERRAM--KREYEEFKIRINALVAKAQK
TaCESA5B 417 FCKKHNIEPRAPEFYFQQKIDYLKDKIQPSFVKERRAM--KREYEEFKIRINALVAKAQK
HvCesA9 20 FCKKHNIEPRAPEFYFQQKIDYLKDKIQPSFVKERRAM--KREYEEFKIRINALVAKAQK
ZmCESA5 416 FCKKYNIEPXAPEWYFAQKIDYLKDKVQTSFVKERRAM--KREYEEFKVRINGLVAKAQK
OsCESA2 412 FCKKYSIEPRAPEWYFAQKIDYLKDKVQASFVKDRRAM--KREYEEFKVRVNALVAKAQK
HvCESA3 413 FCKKYNIEPRAPEWYFAQKIDFLKDKVQTSFVKDRRAM--KREYEEFKVRVNSLVAKAEK
TaCESA3B 429 FCKKYNIEPRAPEWYFAQKIDFLKDKVQTSFIKDRRAM--KREYEEFKVRVNSLVAKAEK
TaCESA3D 429 FCKKYNIEPRAPEWYFAQKIDFLKDKVQTSFVKDRRAM--KREYEEFKVRVNSLVAKAEK
TaCESA3A 250 FCKKYNIEPRAPEWYFAQKIDFLKDKVQTSFVKDRRAM--KREYEEFKVRVNSLVAKAEK
HvCESA1 419 FVKKYDIEPRAPEWYFSQKIDYLKDKVQPSFVKDRRAM--KREYEEFKIRINGLVSKALK
TaCESA1D 351 FVKKYDIEPRAPEFYFCQKIDYLKDKVQPSFVKDRRAM--KREYEEFKIRINALVSKALK
TaCESA1A 419 FVKKYDIEPRAPEFYFCQKIDYLKDKVQPSFVKDRRAM--KREYEEFKIRINALVSKALK
TaCESA1B 419 FVKKYDIEPRAPEFYFCQKIDYLKDKVQPSFVKDRRAM--KREYEEFKIRINALVSKALK
OsCESA8 419 FVKKYNIEPRAPEWYFSQKIDYLKDKVHPSFVKDRRAM--KREYEEFKVRINGLVAKAQK
ZmCESA4 417 FVKKYNIEPRAPEWYFSQKIDYLKDKVHPSFVKDRRAM--KREYEEFKVRVNGLVAKAQK
ZmCESA9 419 FVKKYNIEPRAPEWYFSQKIDYLKDKVHPSFVKDRRAM--KREYEEFKIRVNGLVAKAQK
OsCESA10 71 VPQLGV-------------ADGSKLID--------VASVDVCLPALVYVCREKRRGHAHH
OsCESA11 249 VPEEGWIMSDGTPWPGNNSRDHPAMIQVLLGHPGDRDVDGGELPRLFYVSREKRPGFRHH
ZmCESA10 434 KPEEGWVMQDGTPWPGNNTRDHPGMIQVYLGNQGALDVEGHELPRLVYVSREKRPGYNHH
OsCESA7 414 KPEEGWVMQDGTPWPGNNTRDHPGMIQVYLGSQGALDVEGSELPRLVYVSREKRPGYNHH
TaCESA4D 178 ------------------------------------------------------------
TaCESA4B 407 KPEEGWVMQDGTPWPGNNTRDHPGMIQVYLGSQGALDVEGHELPRLVYVSREKRPGHNHH
TaCESA4A 407 KPEEGWVMQDGTPWPGNNTRDHPGMIQVYLGSQGALDVEGHELPRLVYVSREKRPGHNHH
HvCESA4 404 KPEEGWVMQDGTPWPGNNTRDHPGMIQVYLGSQGALDVEGHELPRLVYVSREKRPGHNHH
ZmCESA11 383 TPDEGWIMQDGTPWPGNNPRDHPGMIQVFLGETGARDFDGNELPRLVYVSREKRPGYQHH
OsCESA4 389 TPEEGWIMQDGTPWPGNNPRDHPGMIQVFLGETGARDFDGNELPRLVYVSREKRPGYQHH
HvCESA7/5 385 TPEEGWVMQDGTPWPGNNSRDHPGMIQVFLGETGARDYDGNELPRLVYVSREKRPGYQHH
TaCESA7B 383 TPEEGWVMQDGTPWPGNNSRDHPGMIQVFLGETGARDYDGNELPRLVYVSREKRPGYQHH
TaCESA7D 299 TPEEGWVMQDGTPWPGNNSRDHPGMIQVFLGETGARDYDGNELPRLVYVSREKRPGYQHH
TaCESA8D 322 VPEEGWIMKDGTPWPGNNTRDHPGMIQVFLGHSGGLDTEGNELPRLVYVSREKRPGFQHH
TaCESA8B 467 VPEEGWIMKDGTPWPGNNTRDHPGMIQVFLGHSGGLDTEGNELPRLVYVSREKRPGFQHH
HvCESA8 469 VPDEGWIMKDGTPWPGNNTRDHPGMIQVFLGHSGGLDTEGNELPRLVYVSREKRPGFQHH
OsCESA9 468 VPAEGWIMKDGTPWPGNNTRDHPGMIQVFLGHSGGHDTEGNELPRLVYVSREKRPGFQHH
ZmCESA12 466 VPAEGWIMKDGTPWPGNNTRDHPGMIQVFLGHSGGHDTEGNELPRLVYVSREKRPGFQHH
ZmCESA13 466 VPAEGWIMKDGTPWPGNNTRDHPGMIQVFLGHSGGHDTEGNELPRLVYVSREKRPGFQHH
OsCESA6 480 VPEEGWTMQDGSPWPGNNVRDHPGMIQVFLGQSGGRDVEGNELPRLVYVSREKRPGYNHH
ZmCESA8 482 VPEEGWTMQDGSPWPGNNVRDHPGMIQVFLGQSGGRDVEGNELPRLVYVSREKRPGYNHH
TaCESA2A 478 VPEEGWTMQDGTPWPGNNVRDHPGMIQVFLGQSGGLDVEGNELPRLVYVSREKRPGYNHH
TaCESA2B 478 VPEEGWTMQDGTPWPGNNVRDHPGMIQVFLGQSGGLDVEGNELPRLVYVSREKRPGYNHH
TaCESA2D 478 VPEEGWTMQDGTPWPGNNVRDHPGMIQVFLGQSGGLDVEGNELPRLVYVSREKRPGYNHH
HvCESA2 478 VPEEGWTMQDGTPWPGNNVRDHPGMIQVFLGQSGGLDVEGNELPRLVYVSREKRPGYNHH
ZmCESA7 474 VPEEGWTMQDGTPWPGNNVRDHPGMIQVFLGQSGGLDCEGNELPRLVYVSREKRPGYNHH
ZmCESA6 445 VPEEGWTMQDGTPWPGNNVRDHPGMIQVFLGQSGGHDVEGNELPRLVYVSREKRPGYNHH
OsCESA3 480 VPEEGWTMQDGTPWPGNNVRDHPGMIQVFLGQSGGHDIEGNELPRLVYVSREKRPGYNHH
OsCESA5 479 VPEEGWTMQDGTPWPGNNVRDHPGMIQVFLGQSGGHDVEGNELPRLVYVSREKRPGYNHH
TaCESA9B 78 VPEEGWIMQDGTPWPGNNTRDHPGMIQVFLGHSGGLDTEGNELPRLVYVSREKRPGFQHH
ZmCESA3 471 IPEEGWTMADGTPWPGNNPRDHPGMIQVFLGHSGGLDTDGNELPRLVYVSREKRPGFQHH
ZmCESA1 472 VPEEGWTMADGTAWPGNNPRDHPGMIQVFLGHSGGLDTDGNELPRLVYVSREKRPGFQHH
ZmCESA2 471 VPEEGWTMADGTAWPGNNPRDHPGMIQVFLGHSGGLDTDGNELPRLVYVSREKRPGFQHH
OsCESA1 473 VPEEGWTMADGTAWPGNNPRDHPGMIQVFLGHSGGLDTDGNELPRLVYVSREKRPGFQHH
TaCESA5A 476 VPEEGWTMADGTAWPGNNPRDHPGMIQVFLGHSGGLDTEGNELPWLVYVSREKRPGFQHH
HvCESA6 471 VPEEGWTMADGTAWPGNNPRDHPGMIQVFLGHSGGLDTDGNELPRLVYVSREKRPGFQHH
TaCESA6B 466 VPEEGWTMADGTAWPGNNPRDHPGMIQVFLGHSGGLDTDGNELPRLVYVSREKRPGFQHH
TaCESA6A 472 VPEEGWTMADGTAWPGNNPRDHPGMIQVFLGHSGGLDTDGNELPRLVYVSREKRPGFQHH
TaCESA5D 476 VPEEGWTMADGTAWPGNNPRDHPGMIQVFLGHSGGLDTDGNELPRLVYVSREKRPGFQHH
TaCESA5B 475 VPEEGWTMADGTAWPGNNPRDHPGMIQVFLGHSGGLDTDGNELPRLVYVSREKRPGFQHH
HvCesA9 78 VPEEGWTMADGTAWPGNNPRDHPGMIQVFLGHSGGLDTDGNELPRLVYVSREKRPGFQHH
ZmCESA5 474 VPEEGWIMQDGTPWPGNNTRDHPGMIQVFLGHSGGLDVEGNELPRLVYVSREKRPGFQHH
OsCESA2 470 VPEEGWIMQDGTPWPGNNTRDHPGMIQVFLGHSGGLDTEGNELPRLVYVSREKRPGFQHH
HvCESA3 471 VPEEGWIMQDGTPWPGNNTRDHPGMLQVFLGHSGGLDTDGNELPRLVYVSREKRPGFQHH
TaCESA3B 487 VPEEGWIMQDGTPWPGNNTRDHPGMLRVFLGHSGGLDSDGNELPRLVYVSREKRAGFQHH
TaCESA3D 487 VPEEGWIMQDGTPWPGNNTRDHPGMLQVFLGHSGGLDSDGNELPRLVYVSREKRAGFQHH
TaCESA3A 308 VPEEGWIMQDGTPWPGNNTRDHPGMLQVFLGHSGGLDSDGNELPRLVYVSREKRAGFQHH
HvCESA1 477 VPEEGWIMQDGTPWPGNNTRDHPGMIQVFLGHSGGLDTEGNELPRLVYVSREKRPGFQHH
TaCESA1D 409 VPEEGWIMQDGTPWPGNNTRDHPGMIQVFLGHSGGLDTEGNELPRLVYVSREKRPGFQHH
TaCESA1A 477 VPEEGWIMQDGTPWPGNNTRDHPGMIQVFLGHSGGLDTEGNELPRLVYVSREKRPGFQHH
TaCESA1B 477 VPEEGWIMQDGTPWPGNNTRDHPGMIQVFLGHSGGLDTEGNELPRLVYVSREKRPGFQHH
OsCESA8 477 VPEEGWIMQDGTPWPGNNTRDHPGMIQVFLGHSGGLDTEGNELPRLVYVSREKRPGFQHH
ZmCESA4 475 VPEEGWIMQDGTPWPGNNTRDHPGMIQVFLGHSGGLDTEGNELPRLVYVSREKRPGFQHH
ZmCESA9 477 VPEEGWIMQDGTPWPGNNTRDHPGMIQVFLGHSGGLDTEGNELPRLVYVSREKRPGFQHH
OsCESA10 110 RKAGAMN-----------APFILDLDCDHYVNNSQALRAGICFMIERGGGGAAEDAVAVA
OsCESA11 309 GKAGAMNALLRVSAVLTNGAYVLNLDCDHCVNNSSALREAMCFMMDPVAGN------RTC
ZmCESA10 494 KKAGAMNALVRVSAVLTNAPFILNLDCDHYVNNSKAVREAMCFLMDPQLGK------KLC
OsCESA7 474 KKAGAMNSLVRVSAVLTNAPFILNLDCDHYVNNSKAVREAMCFLMDKQLGK------KLC
TaCESA4D 178 ------------------------------------------------------------
TaCESA4B 467 KKAGAMNALVRVSAVLTNAPFILNLDCDHYVNNSKAVREAMCFLMDPQLGK------KLC
TaCESA4A 467 KKAGAMNALVRVSAVLTNAPFILNLDCDHYVNNSKAVREAMCFLMDPQLGK------KLC
HvCESA4 464 KKAGAMNALVRVSAVLTNAPFILNLDCDHYVNNSKAVREAMCFLMDPQLGK------KLC
ZmCESA11 443 KKAGAMNALVRVSAVLTNAPYILNLDCDHYVNNSKAVREAMCFMMDPTVGR------DVC
OsCESA4 449 KKAGAMNALVRVSAVLTNAPYILNLDCDHYVNNSKAVREAMCFMMDPSVGR------DVC
HvCESA7/5 445 KKAGAMNALVRVSAVLTNAPYILNLDCDHYVNNSKAVREAMCFMMDPSVGR------DVC
TaCESA7B 443 KKAGAMNALVRVSAVLTNAPYILNLDCDHYVNNSKAVREAMCFMMDPSVGR------DVC
TaCESA7D 359 KKAGAMNALVRVSAVLTNAPYILNLDCDHYVNNSKAVREAMCFMMDPSVGR------DVC
TaCESA8D 382 KKAGAMNALIRVSAVLTNAPFMLNLDCDHYINNSKAIRESMCFLMDPQVGR------KVC
TaCESA8B 527 KKAGAMNALIRVSAVLTNAPFMLNLDCDHYINNSKAIRESMCFLMDPQVGR------KVC
HvCESA8 529 KKAGAMNALIRVSAVLTNAPFMLNLDCDHYINNSKAIRESMCFLMDPQVGR------KVC
OsCESA9 528 KKAGAMNALIRVSAVLTNAPFMLNLDCDHYINNSKAIREAMCFLMDPQVGR------KVC
ZmCESA12 526 KKAGAMNALIRVSAVLTNAPFMLNLDCDHYINNSKAIREAMCFLMDPQVGR------KVC
ZmCESA13 526 KKAGAMNALIRVSAVLTNAPFMLNLDCDHYINNSKAIREAMCFLMDPQVGR------KVC
OsCESA6 540 KKAGAMNALVRVSAVLSNAPYLLNLDCDHYINNSKAIREAMCFMMDPLVGK------KVC
ZmCESA8 542 KKAGAMNALVRVSAVLSNAAYLLNLDCDHYINNSKAIKEAMCFMMDPLVGK------KVC
TaCESA2A 538 KKAGAMNALVRVSAVLTNAPYMLNLDCDHYVNNSKAVKEAMCFMMDPLVGK------KVC
TaCESA2B 538 KKAGAMNALVRVSAVLTNAPYMLNLDCDHYVNNSKAVKEAMCFMMDPLVGK------KVC
TaCESA2D 538 KKAGAMNALVRVSAVLTNAPYMLNLDCDHYVNNSKAVKEAMCFMMDPLVGK------KVC
HvCESA2 538 KKAGAMNALVRVSAVLTNAPYMLNLDCDHYVNNSKAVKEAMCFMMDPLVGK------KVC
ZmCESA7 534 KKAGAMNALVRVSAVLTNAPYLLNLDCDHYINNSKAIKEAMCFMMDPLLGK------KVC
ZmCESA6 505 KKAGAMNALVRVSAVLTNAPYLLNLDCDHYINNSKAIKEAMCFMMDPLLGK------KVC
OsCESA3 540 KKAGAMNALVRVSAVLTNAPYMLNLDCDHYINNSKAIKEAMCFMMDPLVGK------KVC
OsCESA5 539 KKAGAMNALVRVSAVLTNAPYMLNLDCDHYINNSKAIKEAMCFMMDPLVGK------KVC
TaCESA9B 138 KKAGAMNALIRVSAVLTNGAYLLNVDCDHYFNSSKALREAMCFMMDPALGR------KTC
ZmCESA3 531 KKAGAMNALIRVSAVLTNGAYLLNVDCDHYFNSSKALREAMCFMMDPALGR------KTC
ZmCESA1 532 KKAGAMNALIRVSAVLTNGAYLLNVDCDHYFNSSKALREAMCFMMDPALGR------KTC
ZmCESA2 531 KKAGAMNALIRVSAVLTNGAYLLNVDCDHYFNSSKALREAMCFMMDPALGR------KTC
OsCESA1 533 KKAGAMNALIRVSAVLTNGAYLLNVDCDHYFNSSKALREAMCFMMDPALGR------KTC
TaCESA5A 536 KKAGAMNALIRVSAVLTNGAYLLNVDCDHYFNSSKALREAMCFMMDPALGR------KTC
HvCESA6 531 KKAGAMNALIRVSAVLTNGAYLLNVDCDHYFNSSKALREAMCFMMDPALGR------KTC
TaCESA6B 526 KKAGAMNALIRVSAVLTNGAYLLNVDCDHYFNSSKALREAMCFMMDPALGR------KTC
TaCESA6A 532 KKAGAMNALIRVSAVLTNGAYLLNVDCDHYFNSSKALREAMCFMMDPALGR------KTC
TaCESA5D 536 KKAGAMNALIRVSAVLTNGAYLLNVDCDHYFNSSKALREAMCFMMDPALGR------KTC
TaCESA5B 535 KKAGAMNALIRVSAVLTNGAYLLNVDCDHYFNSSKALREAMCFMMDPALGR------KTC
HvCesA9 138 KKAGAMNALIRVSAVLTNGAYLLNVDCDHYFNSSKALREAMCFMMDPALGR------KTC
ZmCESA5 534 KKAGAMNALVRVSAVLTNGQYMLNLDCDHYINNSKALREAMCFLMDPNLGR------NVC
OsCESA2 530 KKAGAMNALVRVSAVLTNGQYLLNLDCDHYINNSKALREAMCFLMDPNLGR------RVC
HvCESA3 531 KKAGAMNALVRVSAVLTNGQYMLNLDCDHYINNSSALREAMCFLMDPNLGR------KIC
TaCESA3B 547 KKAGAMNALVRVSAVLTNGQYMLNLDCDHYINNSSALREAMCFLMDPNLGR------KIC
TaCESA3D 547 KKAGAMNALVRVSAVLTNGQYMLNLDCDHYINNSSALREAMCFLMDPNLGR------KIC
TaCESA3A 368 KKAGAMNALVRVSAVLTNGQYMLNLDCDHYINNSSALREAMCFLMDPNLGR------KIC
HvCESA1 537 KKAGAMNALVRVSAVLTNGQYMLNLDCDHYINNSKAVREAMCFLMDPNLGP------QVC
TaCESA1D 469 KKAGAMNALVRVSAVLTNGQYMLNLDCDHYINNSKAVREAMCFLMDPNLGP------QVC
TaCESA1A 537 KKAGAMNALVRVSAVLTNGQYMLNLDCDHYINNSKAVREAMCFLMDPNLGP------QVC
TaCESA1B 537 KKAGAMNALVRVSAVLTNGQYMLNLDCDHYINNSKAVREAMCFLMDPNLGP------QVC
OsCESA8 537 KKAGAMNALVRVSAVLTNGQYMLNLDCDHYINNSKALREAMCFLMDPNLGR------SVC
ZmCESA4 535 KKAGAMNALVRVSAVLTNGQYMLNLDCDHYINNSKALREAMCFLMDPNLGR------SVC
ZmCESA9 537 KKAGAMNALVRVSAVLTNGQYMLNLDCDHYINNSKALREAMCFLMDPNLGR------SVC
OsCESA10 159 FVQFPQRVDGVDPSDRYANHNRVFFDCTELGLDGLQGPIYVGTGCLFRRVALYSVDLPRW
OsCESA11 363 FVQFALRDSG--------GGDSVFFDIEMKCLDGIQGPVYVGSGCCFSRKALYGFEPAAA
ZmCESA10 548 YVQFPQRFDGIDRHDRYANRNVVFFDINMKGLDGIQGPVYVGTGCVFNRQALYGYDPPRP
OsCESA7 528 YVQFPQRFDGIDRHDRYANRNTVFFDINMKGLDGIQGPVYVGTGTVFNRQALYGYDPPRP
TaCESA4D 178 ------------------------------------------------------------
TaCESA4B 521 YVQFPQRFDGIDLHDRYANRNVVFFDVTPHPP----------------------------
TaCESA4A 521 YVQFPQRFDGIDLHDRYANRNVVFFDINMKGLDGIQGPVYVGTGCVFNRQALYGYDPPRP
HvCESA4 518 YVQFPQRFDGIDLHDRYANRNVVFFDINMKGLDGIQGPVYVGTGCVFNRQALYGYDPPRP
ZmCESA11 497 YVQFPQRFDGIDRSDRYANRNVVFFDVNMKGLDGLQGPVYVGTGCCFNRQALYGYGPPSL
OsCESA4 503 YVQFPQRFDGIDRSDRYANRNVVFFDVNMKGLDGLQGPVYVGTGCCFYRQALYGYGPPSL
HvCESA7/5 499 YVQFPQRFDGIDRSDRYANRNVVFFDVNMKGLDGIQGPVYVGTGCCFYRQALYGYGPPSL
TaCESA7B 497 YVQFPQRFDGIDRSDRYANRNVVFFDVNMKGLDGIQGPVYVGTGCCFYRQALYGYGPPSL
TaCESA7D 413 YVQFPQRFDGIDRSDRYANRNVVFFDVNMKGLDGIQGPVYVGTGCCFYRQALYGYGPPSL
TaCESA8D 436 YVQFPQRFDGIDAHDRYANRNTVFFDINMKGLDGIQGPVYVGTGCVFRRQALYGYNPPSG
TaCESA8B 581 YVQFPQRFDGIDAHDRYANRNTVFFDINMKGLDGIQGPVYVGTGCVFRRQALYGYNPPSG
HvCESA8 583 YVQFPQRFDGIDAHDRYANRNTVFFDINMKGLDGIQGPVYVGTGCVFRRQALYGYNPPSG
OsCESA9 582 YVQFPQRFDGIDVHDRYANRNTVFFDINMKGLDGIQGPVYVGTGCVFRRQALYGYNPPKG
ZmCESA12 580 YVQFPQRFDGIDVHDRYANRNTVFFDINMKGLDGIQGPVYVGTGCVFRRQALYGYNPPKG
ZmCESA13 580 YVQFPQRFDGIDMHDRYANRNTVFFDINMKGLDGIQGPVYVGTGCVFRRQALYGYNPPKG
OsCESA6 594 YVQFPQRFDGIDRHDRYANRNVVFFDINMKGLDGIQGPIYVGTGCVFRRQALYGYDAPKT
ZmCESA8 596 YVQFPQRFDGIDKNDRYANRNVVFFDINMKGLDGIQGPIYVGTGCVFRRQALYGYDAPKT
TaCESA2A 592 YVQFPQRFDSIDRHDRYANKNVVFFDINMKGLDGIQGPIYVGTGCVFRRQALYGYDAPKT
TaCESA2B 592 YVQFPQRFDSIDRHDRYANKNVVFFDINMKGLDGIQGPIYVGTGCVFRRQALYGYDAPKT
TaCESA2D 592 YVQFPQRFDSIDRHDRYANKNVVFFDINMKGLDGIQGPIYVGTGCVFRRQALYGYDAPKT
HvCESA2 592 YVQFPQRFDSIDRHDRYANKNVVFFDINMKGLDGIQGPIYVGTGCVFRRQALYGYDAPKT
ZmCESA7 588 YVQFPQRFDGIDRHDRYANRNVVFFDINMKGLDGIQGPIYVGTGCVFRRQALYGYDAPKT
ZmCESA6 559 YVQFPQRFDGIDRHDRYANRNVVFFDINMKGLDGIQGPIYVGTGCVFRRQALYGYDAPKT
OsCESA3 594 YVQFPQRFDGIDRHDRYANRNVVFFDINMKGLDGIQGPIYVGTGCVFRRQALYGYDAPKT
OsCESA5 593 YVQFPQRFDGIDRHDRYANRNVVFFDINMKGLDGIQGPIYVGTGCVFRRQALYGYDAPKS
TaCESA9B 192 YVQFPQRFDGIDLHDRYANRNIVFFDINMKGLDGIQGPMYVGTGCCFNRQALYGYDPVLT
ZmCESA3 585 YVQFPQRFDGIDLHDRYANRNIVFFDINMKGLDGIQGPVYVGTGCCFNRQALYGYDPVLT
ZmCESA1 586 YVQFPQRFDGIDLHDRYANRNIVFFDINMKGLDGIQGPVYVGTGCCFNRQALYGYDPVLT
ZmCESA2 585 YVQFPQRFDGIDLHDRYANRNIVFFDINMKGLDGIQGPVYVGTGCCFNRQALYGYDPVLT
OsCESA1 587 YVQFPQRFDGIDLHDRYANRNIVFFDINMKGLDGIQGPVYVGTGCCFNRQALYGYDPVLT
TaCESA5A 590 YVQFPQRFDGIDLHDRYANRNIVFFDINMKGLDGIQGPMYVGTGCCFNRQALYGYDPVLT
HvCESA6 585 YVQFPQRFDGIDLHDRYANRNIVFLDINMKGLDGIQGPMYVGTGCCFNRQALYGYDPVLT
TaCESA6B 580 YVQFPQRFDGIDLHDRYANRNIVFFDINMKGLDGIQGPMYVGTGCCFNRQALYGYDPVLT
TaCESA6A 586 YVQFPQRFDGIDLHDRYANRNIVFFDINMKGLDGIQGPMYVGTGCCFNRQALYGYDPVLT
TaCESA5D 590 YVQFPQRFDGIDLHDRYANRNIVFFDINMKGLDGIQGPMYVGTGCCFNRQALYGYDPVLT
TaCESA5B 589 YVQFPQRFDGIDLHDRYANRNIVFFDINMKGLDGIQGPMYVGTGCCFNRQALYGYDPVLT
HvCesA9 192 YVQFPQRFDGIDLHDRYANRNIVFFDINMKGLDGIQGPMYVGTGCCFNRQALYGYDPVLT
ZmCESA5 588 YVQFPQRFDGIDRNDRYANRNTVFFDINLRGLDGIQGPVYVGTGCVFNRTALYGYEPPVK
OsCESA2 584 YVQFPQRFDGIDRNDRYANRNTVFFDINLRGLDGLQGPVYVGTGCVFNRTALYGYEPPIK
HvCESA3 585 YVQFPQRFDGIDTNDRYANRNTVFFDINLRGLDGIQGPVYVGTGCVFNRTALYGYEPPMK
TaCESA3B 601 YVQFPQRFDGIDTNDRYANRNTVFFDINLRGLDGIQGPVYVGTGCVFNRTALYGYEPPMK
TaCESA3D 601 YVQFPQRFDGIDTNDRYANRNTVFFDINLRGLDGIQGPVYVGTGCVFNRTALYGYEPPMK
TaCESA3A 422 YVQFPQRFDGIDTNDRYANRNTVFFDINLRGLDGIQGPVYVGTGCVFNRTALYGYEPPMK
HvCESA1 591 YVQFPQRFDGIDRNDRYANRNTVFFDINLRGLDGIQGPVYVGTGCVFNRTAIYGYEPPIK
TaCESA1D 523 YVQFPQRFDGIDRNDRYANRNTVFFDINLRGLDGIQGPVYVGTGCVFNRTAIYGYEPPIK
TaCESA1A 591 YVQFPQRFDGIDRNDRYANRNTVFFDINLRGLDGIQGPVYVGTGCVFNRTAIYGYEPPIK
TaCESA1B 591 YVQFPQRFDGIDRNDRYANRNTVFFDINLRGLDGIQGPVYVGTGCVFNRTAIYGYEPPIK
OsCESA8 591 YVQFPQRFDGIDRNDRYANRNTVFFDINLRGLDGIQGPVYVGTGCVFNRTALYGYEPPIK
ZmCESA4 589 YVQFPQRFDGIDRNDRYANRNTVFFDINLRGLDGIQGPVYVGTGCVFNRTALYGYEPPIK
ZmCESA9 591 YVQFPQRFDGIDRNDRYANRNTVFFDINLRGLDGIQGPVYVGTGCVFNRTALYGYEPPIK
OsCESA10 219 RPRRSLGCR----------LLGEDERLWSRL------KQMVI------------------
OsCESA11 415 ADDGDD-MDTAADWRR-MCCFGRGKRMNAMRRS---------------------------
ZmCESA10 608 EKRPKMTCDCWPSWCCCCCCFGGGKRGKARKDKKGDGG------EEPRRGLLGFYRKRSK
OsCESA7 588 EKRPKMTCDCWPSWCCCCCCFGGGKRGKSHKNKKGGGGGEGGGLDEPRRGLLGFYKKRSK
TaCESA4D 178 ------------------------------------------------------------
TaCESA4B 553 -SLPPSDTS---------------------------------------------------
TaCESA4A 581 EKRPKMTCDCWPSWCCCCCCFGGGKHRKSDKDKKG-------GDDEPRRGLLGFYKKRGK
HvCESA4 578 EKRPKMTCDCWPSWCCCCCCFGGGKHRKSSKDKKGGGG----GDDEPRRGLLGFYKKRGK
ZmCESA11 557 PALPKSSIC---SWCC-CCCPKKKVER--SE------REI--------------------
OsCESA4 563 PALPKSSVC---SWCC-CCCPKKKAEK--SE------KEM--------------------
HvCESA7/5 559 PALPKSSAC---SFCC--CCPKNKVEK--TE------KEM--------------------
TaCESA7B 557 PALPKSSAC---SFCC-CCCPKKKVEK--TE------KEM--------------------
TaCESA7D 473 PALPKSSAC---SFCC-CCCPKKKVEK--TE------KEM--------------------
TaCESA8D 496 PKRPKM------VTCDCCPCFGRKKRKGGK------------------------------
TaCESA8B 641 PKRPKM------VTCDCCPCFGRKKRKGGK------------------------------
HvCESA8 643 PKRPKM------VTCDCCPCFGRKKRKGGK------------------------------
OsCESA9 642 PKRPKM------VTCDCCPCFGRKKRKHGK------------------------------
ZmCESA12 640 PKRPKM------VTCDCCPCFGRKKRKHAK------------------------------
ZmCESA13 640 PKRPKM------VTCDCCPCFGRKKRKDAK------------------------------
OsCESA6 654 KKPPSRTCNCWPKWCCCCCC-GNRHTKKKTTKPKPE-KKK--------------------
ZmCESA8 656 KKPPSRTCNCWPKWCLSCCC-SRNKNKKKTTKPKTE-KKK--------------------
TaCESA2A 652 KKPPSRTCNCWPKWCVCCFCFGNRKNKKKVTKPKTE-KKK--------------------
TaCESA2B 652 KKPPSRTCNCWPKWCVCCFCFGNRKNKKKVTKPKTE-KKK--------------------
TaCESA2D 652 KKPPSRTCNCWPKWCVCCFCFGNRKNKKKVTKPKTE-KKK--------------------
HvCESA2 652 KKPPSRTCNCWPKWCVCCFCFGNRKNKKKVTKPKTE-KKK--------------------
ZmCESA7 648 KKPPSRTCNCWPKWCFCCCCFGNRKQKKTTKP-KTE-KKK--------------------
ZmCESA6 619 KKPPSRTCNCWPKWCICCCCFGNRKTKKKTKTSKPKFEKI--------------------
OsCESA3 654 KKPPSRTCNCWPKWCICCCCFGDRKSKKKTTKPKTE-KKK--------------------
OsCESA5 653 KKPPSRTCNCWPKWCICCCCFGNRTNKKKTAKPKTE-KKK--------------------
TaCESA9B 252 EADLE------PNIVVKSCCGG-RKKKSKS---YM--DNK--------------------
ZmCESA3 645 EADLE------PNIIIKSCCGG-RKKKDKS---YI--DSK--------------------
ZmCESA1 646 EADLE------PNIVIKSCCGR-RKKKNKS---YM--DSQ--------------------
ZmCESA2 645 EADLE------PNIVVKSCCGR-RKRKNKS---YM--DSQ--------------------
OsCESA1 647 EADLE------PNIVVKSCCGG-RKKKSKS---YM--DSK--------------------
TaCESA5A 650 EADLE------PNIVVKSCCGG-RKKKSKS---YM--DNK--------------------
HvCESA6 645 EADLE------PNIVVKSCCGG-RKKKNKS---YM--DTK--------------------
TaCESA6B 640 EADLE------PNIVVKSCCGG-RKKKSKS---YM--DNK--------------------
TaCESA6A 646 EADLE------PNIVVKSCCGG-RKKKSKS---YM--DNK--------------------
TaCESA5D 650 EADLE------PNIVVKSCCGG-RKKKSKS---YM--DNK--------------------
TaCESA5B 649 EADLE------PNIVVKSCCGG-RKKKSKS---YM--DNK--------------------
HvCesA9 252 EADLE------PNIVVKSCCGG-RKKKNKS---YM--DNK--------------------
ZmCESA5 648 KK-K--------PGFFSSLCGG-RKKTSKS---K-KSSEK--------------------
OsCESA2 644 QK-R--------PGYFSSLCGG-RKKTKKS---KEKSTEK--------------------
HvCESA3 645 KK-E--------SGLFSKLCGG-RTSK-------LK--ES--------------------
TaCESA3B 661 SK-E--------SGLFSKLCGG-RTSK-------SKSTGS--------------------
TaCESA3D 661 SK-E--------SGLFSKLCGG-RTSK-------SKSTGS--------------------
TaCESA3A 482 NK-E--------SGLFSKLCGG-RTSK-------SKSTGS--------------------
HvCESA1 651 AK-K--------PSFLASLCGG-KKKASKS---KKRSSDK--------------------
TaCESA1D 583 AK-K--------PGFLASLCGG-KKKASKS---KKRSSDK--------------------
TaCESA1A 651 AK-K--------PGFLASLCGG-KKKASKS---KKRSSDK--------------------
TaCESA1B 651 AK-K--------PGFLASLCGG-KKKTSKS---KKRSSDK--------------------
OsCESA8 651 QKKK--------GSFLSSLCGG-RKKASKS---KKKSSDK--------------------
ZmCESA4 649 QK-K--------GGFLSSLCGG-RKKASKS---KK-GSDK--------------------
ZmCESA9 651 QK-K--------GGFLSSLCGG-RKKGSKS---KK-GSDK--------------------
OsCESA10 ------------------------------------------------------------
OsCESA11 446 -------------------MSAVPLLDSEDDSDEQEEEEAAGRRRRLRAYRAALERHFGQ
ZmCESA10 662 KDKLGGGSVAGSKKGGGLYKKHQRAFELEEIEEGLEG--YDELERSSLMSQKSFEKRFGQ
OsCESA7 648 KDKLGGGAASL-AGGKKGYRKHQRGFELEEIEEGLEG--YDELERSSLMSQKSFEKRFGQ
TaCESA4D 178 ------------------------------------------------------------
TaCESA4B 561 ------------------------------------------------------------
TaCESA4A 634 KDKLGGGPK------KGSYRKQQRGYELEEIEEGIEG--YDELERSSLMSQKSFQKRFGQ
HvCESA4 634 KDKLGGGPK------KGSYRKQQRGYELEEIEEGIEG--YDELERSSLMSQKSFQKRFGQ
ZmCESA11 585 ------------NRDSRREDLESAIFNLREIDNY------DEYERSMLISQMSFEKSFGL
OsCESA4 591 ------------HRDSRREDLESAIFNLREIDNY------DEYERSMLISQMSFEKSFGL
HvCESA7/5 586 ------------HRDSRREDLESAIFNLREIDNY------DEYERSMLISQMSFEKSFGQ
TaCESA7B 585 ------------HRDSRREDLESAIFNLREIDNY------DEYERSMLISQMSFEKSFGQ
TaCESA7D 501 ------------HRDSRREDLESAIFNLREIDNY------DEYERSMLISQMSFEKSFGQ
TaCESA8D 520 ----------------------------DGLPEGV-ADGGMDGDKEQMMSQMNFEKRFGQ
TaCESA8B 665 ----------------------------DGLPEGV-ADGGMDGDKEQMMSQMNFEKRFGQ
HvCESA8 667 ----------------------------DGLPEGV-ADGGMDGDKEQMMSQMNFEKRFGQ
OsCESA9 666 ----------------------------DGLPEAVAADGGMDSDKEMLMSQMNFEKRFGQ
ZmCESA12 664 ----------------------------DGLPEGT-ADMGVDSDKEMLMSHMNFEKRFGQ
ZmCESA13 664 ----------------------------DGLPEGT-ADIGVDSDKEMLMSQMNFEKRFGQ
OsCESA6 692 ------------RLFFKKAENQSPAYALGEIEEG-APG--AETDKAGIVNQQKLEKKFGQ
ZmCESA8 694 ------------RLFFKKAENPSPAYALGEIDEG-APG--ADIEKAGIVNQQKLEKKFGQ
TaCESA2A 691 ------------RLFFKKEENQSPAYALSEIDEA-AAG--AETQKAGIVNQQKLEKKFGQ
TaCESA2B 691 ------------RLFFKKEENQSPAYALSEIDEA-AAG--AETQKAGIVNQQKLEKKFGQ
TaCESA2D 691 ------------RLFFKKEENQSPAYALSEIDEA-AAG--AETQKAGIVNQQKLEKKFGQ
HvCESA2 691 ------------RLFFKKEENQSPAYALSEIDGA-AAG--AETQKAGIVNQQKLEKKFGQ
ZmCESA7 686 ------------LLFFKKEENQSPAYALGEIDEA-APG--AENEKAGIVNQQKLEKKFGQ
ZmCESA6 659 ------------KKLFKKKENQAPAYALGEIDEA-APG--AENEKASIVNQQKLEKKFGQ
OsCESA3 693 ------------RSFFKRAENQSPAYALGEIEEG-APG--AENEKAGIVNQQKLEKKFGQ
OsCESA5 692 ------------RLFFKRAENQSPAYALGEIDEG-APG--AENEKAGIVNQQKLEKKFGQ
TaCESA9B 280 ------------NRMMKRTESSAPIFNMDDIEEG-IEG--YEDERSMLMSQKRLEKRFGQ
ZmCESA3 673 ------------NRDMKRTESSAPIFNMEDIEEG-FEG--YEDERSLLMSQKSLEKRFGQ
ZmCESA1 674 ------------SRIMKRTESSAPIFNMEDIEEG-IEG--YEDERSVLMSQRKLEKRFGQ
ZmCESA2 673 ------------SRIMKRTESSAPIFNMEDIEEG-IEG--YEDERSVLMSQRKLEKRFGQ
OsCESA1 675 ------------NRMMKRTESSAPIFNMEDIEEG-IEG--YEDERSVLMSQKRLEKRFGQ
TaCESA5A 678 ------------NRMMKRTESSAPIFNMDDIEEG-IEG--YEDERSMLMSQKRLEKRFGQ
HvCESA6 673 ------------TRMMKRTESSAPIFNMEDIEEG-IEG--YEDERSMLMSQKRLEKRFGQ
TaCESA6B 668 ------------NRMMKRTESSAPIFNMDDIEEG-IEG--YEDERSMLMSQKRLEKRFGQ
TaCESA6A 674 ------------NRMMKRTESSAPIFNMEDIEEG-IEG--YEDERSMLMSQKRLEKRFGQ
TaCESA5D 678 ------------NRMMKRTESSAPIFNMDDIEEG-IEG--YEDERSMLMSQKRLEKRFGQ
TaCESA5B 677 ------------NRMMKRTESSAPIFNMDDIEEG-IEG--YEDERSMLMSQKRLEKRFGQ
HvCesA9 280 ------------NRMMKRTESSAPIFNMEDIEEG-IEG--YEDERSMLMSQKRLEKRFGQ
ZmCESA5 674 ------------KKSHRHADSSVPVFNLEDIEEG-IEGSQFDDEKSLIMSQMSLEKRFGQ
OsCESA2 671 ------------KKSHKHVDSSVPVFNLEDIEEG-IEGSGFDDEKSLLMSQMSLEKRFGQ
HvCESA3 666 ------------KKSDKHVDGSVPVFNLEDIEEG-IEGSGFDDEKSLLMSQMSLEKRFGQ
TaCESA3B 684 ------------KKSDKHADGSVPVFNLEDIEEG-IEGSGFDDEKSLLMSQMSLEKRFGQ
TaCESA3D 684 ------------KKSDKHADGSVPMFNLEDIEEG-IEGSGFDDEKSLLMSQMSLEKRFGQ
TaCESA3A 505 ------------KKSDKHADGSVPVFNLEDIEEG-IEGSGFDDEKSLLMSQMSLEKRFGQ
HvCESA1 678 ------------KKSNKHVDSSVPVFNLEDIEEG-VEGAGFDDEKSVLMSQMSLEKRFGQ
TaCESA1D 610 ------------KKSNKHVDSSVPVFNLEDIEEG-VEGAGFDDEKSVLMSQMSLEKRFGQ
TaCESA1A 678 ------------KKSNKHVDSSVPVFNLEDIEEG-VEGAGFDDEKSVLMSQMSLEKRFGQ
TaCESA1B 678 ------------KKSNKHVDSSVPVFNLEDIEEG-VEGAGFDDEKSVLMSQMSLEKRFGQ
OsCESA8 679 ------------KKSNKHVDSAVPVFNLEDIEEG-VEGAGFDDEKSLLMSQMSLEKRFGQ
ZmCESA4 675 ------------KKSQKHVDSSVPVFNLEDIEEG-VEGAGFDDEKSLLMSQMSLEKRFGQ
ZmCESA9 677 ------------KKSQKHVDSSVPVFNLEDIEEG-VEGAGFDDEKSLLMSQMSLEKRFGQ
OsCESA10 ------------------------------------------------------------
OsCESA11 487 SPAFIASAFEEQGRRRGGDGGSPDATVAPARSLLKEAIHVVSCAFEERTRWGKEIGWMYG
ZmCESA10 720 SPVFIASTLVEDG-------GLPQGAAADPAALIKEAIHVISCGYEEKTEWGKEIGWIYG
OsCESA7 705 SPVFIASTLVEDG-------GLPQGAAADPAALIKEAIHVISCGYEEKTEWGKEIGWIYG
TaCESA4D 178 ---------------------------------------------------KNDIGWIYG
TaCESA4B 561 ------------------------------------------------CFTPAQIGWIYG
TaCESA4A 686 SPVFIASTLVEDG-------GLPQGAAADPAGLIKEAIHVISCGYEEKTEWGKEIGWIYG
HvCESA4 686 SPVFIASTLVEDG-------GLPQGAAADPAGLIKEAIHVISCGYEEKTEWGKEIGWIYG
ZmCESA11 627 SSVFIESTLMENG-------GVPES--ANPSTLIKEAIHVISCGYEEKTEWGKEIGWIYG
OsCESA4 633 SSVFIESTLMENG-------GVPES--ANPSTLIKEAIHVISCGYEEKTEWGKEIGWIYG
HvCESA7/5 628 SSVFIESTLMENG-------GVPES--ADPSTLIKEAIHVISCGYEEKTEWGKELGWIYG
TaCESA7B 627 SSVFIESTLMENG-------GVPES--VDPSTLIKEAIHVISCGYEEKTEWGKELGWIYG
TaCESA7D 543 SSVFIESTLMENG-------GVPES--VDPSTLIKEAIHVISCGYEEKTEWGKELGWIYG
TaCESA8D 551 SAAFVTSTFMEEG-------GVXXX--X--XXXLKEAIHVISCGYEDKTDWGLELGWIYG
TaCESA8B 696 SAAFVTSTFMEEG-------GVPPS--SSPAALLKEAIHVISCGYEDKTDWGLELGWIYG
HvCESA8 698 SAAFVTSTFMEEG-------GVPPS--SSPAALLKEAIHVISCGYEDKTDWGLELGWIYG
OsCESA9 698 SAAFVTSTLMEEG-------GVPPS--SSPAALLKEAIHVISCGYEDKTDWGLELGWIYG
ZmCESA12 695 SAAFVTSTLMEEG-------GVPPS--SSPAALLKEAIHVISCGYEDKTDWGLELGWIYG
ZmCESA13 695 SAAFVTSTLMEEG-------GVPPS--SSPAALLKEAIHVISCGYEDKTDWGLELGWIYG
OsCESA6 737 SSVFVASTLLENG-------GTLKS--ASPASLLKEAIHVISCGYEDKTDWGKEIGWIYG
ZmCESA8 739 SSVFVASTLLENG-------GTLKS--ASPASLLKEAIHVISCGYEDKTDWGKEIGWIYG
TaCESA2A 736 SAVFVASTLLENG-------GTLRC--DSPASLLKEAIHVIGCGYEDKTDWGKEIGWIYG
TaCESA2B 736 SAVFVASTLLENG-------GTLRC--DSPASLLKEAIHVIGCGYEDKTDWGKEIGWIYG
TaCESA2D 736 SAVFVASTLLENG-------GTLRC--DSPASLLKEAIHVIGCGYEDKTDWGKEIGWIYG
HvCESA2 736 SAVFVASTLLENG-------GTLRC--DSPASLLKEAIHVIGCGYEDKTDWGKEIGWIYG
ZmCESA7 731 SSVFVTSTLLENG-------GTLKS--ASPASLLKEAIHVISCGYEDKTDWGKEIGWIYG
ZmCESA6 704 SSVFVASTLLENG-------GTLKS--ASPASLLKEAIHVISCGYEDKTGWGKDIGWIYG
OsCESA3 738 SSVFVASTLLENG-------GTLKS--ASPASLLKEAIHVISCGYEDKTDWGKEIGWIYG
OsCESA5 737 SSVFVASTLLENG-------GTLKS--ASPASLLKEAIHVISCGYEDKTDWGKEIGWIYG
TaCESA9B 325 SPIFTASTFMTQG-------GIPPS--TNPASLLKEAIHVISCGYEDKTEWGKEIGWIYG
ZmCESA3 718 SPIFIASTFMTQG-------GIPPS--TNPGSLLKEAIHVISCGYEDKTEWGKEIGWIYG
ZmCESA1 719 SPIFIASTFMTQG-------GIPPS--TNPASLLKEAIHVISCGYEDKTEWGKEIGWIYG
ZmCESA2 718 SPIFIASTFMTQG-------GIPPS--TNPASLLKEAIHVISCGYEDKTEWGKEIGWIYG
OsCESA1 720 SPIFIASTFMTQG-------GIPPS--TNPASLLKEAIHVISCGYEDKTEWGKEIGWIYG
TaCESA5A 723 SPIFTASTFMTQG-------GIPPS--TNPASLLKEAIHVISCGYEDKTEWGKEIGWIYG
HvCESA6 718 SPIFTASTFMTQG-------GIPPS--TNPASLLKEAIHVISCGYEDKTEWGKEIGWIYG
TaCESA6B 713 SPIFTASTFMTQG-------GIPPS--TNPASLLKEAIHVISCGYEDKTEWGKEIGWIYG
TaCESA6A 719 SPIFTASTFMTQG-------GIPPS--TNPASLLKEAIHVISCGYEDKTEWGKEIGWIYG
TaCESA5D 723 SPIFTASTFMTQG-------GIPPS--TNPASLLKEAIHVISCGYEDKTEWGKEIGWIYG
TaCESA5B 722 SPIFTASTFMTQG-------GIPPS--TNPASLLKEAIHVISCGYEDKTEWGKEIGWIYG
HvCesA9 325 SPIFTASTFMTQG-------GIPPS--TNPASLLKEAIHVISCGYEDKTEWGKEIGWIYG
ZmCESA5 721 SSVFVASTLMEYG-------GVPQS--ATPESLLKEAIHVISCGYEDKTDWGTEIGWIYG
OsCESA2 718 SSVFVASTLMEYG-------GVPQS--ATPESLLKEAIHVISCGYEDKSDWGTEIGWIYG
HvCESA3 713 SSVFVASTLMEYG-------GVPQS--ATPESLLKEAIHVISCGYEDRSDWGREIGWIYG
TaCESA3B 731 SSVFVASTLMEYG-------GVPQS--ATPESLLKEAIHVISCGYEDRSDWGREIGWIYG
TaCESA3D 731 SSVFVASTLMEYG-------GVPQS--ATPESLLKEAIHVISCGYEDRSDWGREIGWIYG
TaCESA3A 552 SSVFVASTLMEYG-------GVPQS--ATPESLLKEAIHVISCGYEDRSDWGREIGWIYG
HvCESA1 725 SAAFVASTLMEYG-------GVPQS--STPESLLKEAIHVISCGYEDKSEWGTEIGWIYG
TaCESA1D 657 SAAFVASTLMEYG-------GVPQS--STPESLLKEAIHVISCGYEDKSEWGTEIGWIYG
TaCESA1A 725 SAAFVASTLMEYG-------GVPQS--STPESLLKEAIHVISCGYEDKSEWGTEIGWIYG
TaCESA1B 725 SAAFVASTLMEYG-------GVPQS--STPESLLKEAIHVISCGYEDKSEWGTEIGWIYG
OsCESA8 726 SAAFVASTLMEYG-------GVPQS--ATPESLLKEAIHVISCGYEDKTEWGTEIGWIYG
ZmCESA4 722 SAAFVASTLMEYG-------GVPQS--ATPESLLKEAIHVISCGYEDKTEWGTEIGWIYG
ZmCESA9 724 SAAFVASTLMEYG-------GVPQS--ATPESLLKEAIHVISCGYEDKIEWGTEIGWIYG
OsCESA10 ------------------------------------------------------------
OsCESA11 547 G---GVATGFRMHARGWSSAYCSPARPAFRRYARASPADVLAGASRRAVAAMGILLSRRH
ZmCESA10 773 SVTEDILTGFKMHCRGWKSVYCTPTRPAFKGSAPINLSDRLHQVLRWALGSVEIFMS-RH
OsCESA7 758 SVTEDILTGFKMHCRGWKSVYCTPARAAFKGSAPINLSDRLHQVLRWALGSVEIFMS-RH
TaCESA4D 187 SVTEDILTGFKMHCRGWKSVYCTPTRPAFKGSAPINLSDRLHQVLRWAL-----------
TaCESA4B 573 SVTEDILTGFKMHCRGWKSVYCTPTRPAFKGSAPINLTDRLHQVLRWALGSVEIFMS-RH
TaCESA4A 739 SVTEDILTGFKMHCRGWKSVYCTPTRPAFKGSAPINLSDRLHQVLRWALGSVEIFMS-RH
HvCESA4 739 SVTEDILTGFKMHCRGWKSVYCTPTRPAFKGSAPINLSDRLHQVLRWALGSVEIFMS-RH
ZmCESA11 678 SVTEDILTGFKMHCRGWRSIYCMPVRPAFKGSAPINLSDRLHQVLRWALVSVEIFFS-RH
OsCESA4 684 SVTEDILTGFKMHCRGWRSIYCMPIRPAFKGSAPINLSDRLHQVLRWALGSVEIFLS-RH
HvCESA7/5 679 SVTEDILTGFKMHCRGWRSIYCMPIRPAFKGSAPINLSDRLHQVLRWALGSVEIFFS-RH
TaCESA7B 678 SVTEDILTGFKMHCRGWRSIYCMPIRPAFKGSAPINLSDRLHQVLRWALGSVEIFFS-RH
TaCESA7D 594 SVTEDILTGFKMHCRGWRSIYCMPIRPAFKGSAPINLSDRLHQVLRWALGSVEIFFS-RH
TaCESA8D 600 SITEDILTGFKMHCRGWRSIYCMPKLAAFKGSAPINLSDRLNQVLRWALGSVEIFFS-RH
TaCESA8B 747 SITEDILTGFKMHCRGWRSIYCMPKLAAFKGSAPINLSDRLNQVLRWALGSVEIFFS-RH
HvCESA8 749 SITEDILTGFKMHCRGWRSIYCMPKLAAFKGSAPINLSDRLNQVLRWALGSVEIFFS-RH
OsCESA9 749 SITEDILTGFKMHCRGWRSVYCMPKRAAFKGSAPINLSDRLNQVLRWALGSVEIFFS-RH
ZmCESA12 746 SITEDILTGFKMHCRGWRSVYCMPKRAAFKGSAPINLSDRLNQVLRWALGSVEIFFS-RH
ZmCESA13 746 SITEDILTGFKMHCRGWRSVYCMPKRAAFKGSAPINLSDRLNQVLRWALGSVEIFFS-RH
OsCESA6 788 SITEDILTGFKMHCHGWRSIYCIPKRPAFKGSAPLNLSDRLHQVLRWALGSVEIFFS-KH
ZmCESA8 790 SITEDILTGFKMHCHGWRSIYCIPKRPAFKGSAPLNLSDRLHQVLRWALGSVEIFFS-KH
TaCESA2A 787 SVTEDILTGFKMHCHGWRSIYCIPKRPAFKGSAPLNLSDRLNQVLRWALGSIEIFFS-NH
TaCESA2B 787 SVTEDILTGFKMHCHGWRSIYCIPKRPAFKGSAPLNLSDRLNQVLRWALGSIEIFFS-NH
TaCESA2D 787 SVTEDILTGFKMHCHGWRSIYCIPKRPAFKGSAPLNLSDRLNQVLRWALGSIEIFFS-NH
HvCESA2 787 SVTEDILTGFKMHCHGWRSIYCIPKRPAFKGSAPLNLSDRLNQVLRWALGSIEIFFS-NR
ZmCESA7 782 SVTEDILTGFKMHCHGWRSIYCIPKRVAFKGSAPLNLSDRLHQVLRWALGSIEIFFS-NH
ZmCESA6 755 SVTEDILTGFKMHCHGWRSIYCIPKRAAFKGSAPLNLSDRFHQVLRWALGSIEILFS-NH
OsCESA3 789 SVTEDILTGFKMHCHGWRSIYCIPKLPAFKGSAPLNLSDRLHQVLRWALGSVEIFFS-NH
OsCESA5 788 SVTEDILTGFKMHCHGWRSIYCIPKRAAFKGSAPLNLSDRLHQVLRWALGSIEIFFS-NH
TaCESA9B 376 SVTEDILTGFKMHARGWISIYCMPPRPCFKGSAPINLSDRLNQVLRWALGSVEILFS-RH
ZmCESA3 769 SVTEDILTGFKMHARGWISIYCMPLRPCFKGSAPINLSDRLNQVLRWALGSVEILLS-RH
ZmCESA1 770 SVTEDILTGFKMHARGWQSIYCMPPRPCFKGSAPINLSDRLNQVLRWALGSVEILLS-RH
ZmCESA2 769 SVTEDILTGFKMHARGWQSIYCMPPRPCFKGSAPINLSDRLNQVLRWALGSVEILLS-RH
OsCESA1 771 SVTEDILTGFKMHARGWISIYCMPPRPCFKGSAPINLSDRLNQVLRWALGSVEILLS-RH
TaCESA5A 774 SVTEDILTGFKMHARGWISIYCMPPRPCFKGSAPINLSDRLNQVLRWALGSVEILFS-RH
HvCESA6 769 SVTEDILTGFKMHARGWISIYCMPPRPCFKGSAPINLSDRLNQVLRWALGSVEILFS-RH
TaCESA6B 764 SVTEDILTGFKMHARGWISIYCMPPRPCFKGSAPINLSDRLNQVLRWALGSVEILFS-RH
TaCESA6A 770 SVTEDILTGFKMHARGWISIYCMPPRPCFKGSAPINLSDRLNQVLRWALGSVEILFS-RH
TaCESA5D 774 SVTEDILTGFKMHARGWISIYCMPPRPCFKGSAPINLSDRLNQVLRWALGSVEILFS-RH
TaCESA5B 773 SVTEDILTGFKMHARGWISIYCMPPRPCFKGSAPINLSDRLNQVLRWALGSVEILFS-RH
HvCesA9 376 SVTEDILTGFKMHARGWISIYCMPPRPCFKGSAPINLSDRLNQVLRWALGSVEILFS-RH
ZmCESA5 772 SVTEDILTGFKMHARGWRSIYCMPKRPAFKGSAPINLSDRLNQVLRWALGSIEILFS-RH
OsCESA2 769 SVTEDILTGFKMHARGWRSIYCMPKRPAFKGSAPINLSDRLNQVLRWALGSVEILFS-RH
HvCESA3 764 SVTEDILTGFKMHARGWRSIYCMPKRPAFKGSAPINLSDRLNQVLRWALGSVEILFS-RH
TaCESA3B 782 SVTEDILTGFKMHARGWRSIYCMPKRPAFKGSAPINLSDRLNQVLRWALGSVEILFS-RH
TaCESA3D 782 SVTEDILTGFKMHARGWRSIYCMPKRPAFKGSAPINLSDRLNQVLRWALGSVEILFS-RH
TaCESA3A 603 SVTEDILTGFKMHARGWRSIYCMPKRPAFKGSAPINLSDRLNQVLRWALGSVEILFS-RH
HvCESA1 776 SVTEDILTGFKMHARGWRSVYCMPKRPAFKGSAPINLSDRLNQVLRWALGSVEILFS-RH
TaCESA1D 708 SVTEDILTGFKMHARGWRSVYCMPKRPAFKGSAPINLSDRLNQVLRWALGSVEILFS-RH
TaCESA1A 776 SVTEDILTGFKMHARGWRSVYCMPKRPAFKGSAPINLSDRLNQVLRWALGSVEILFS-RH
TaCESA1B 776 SVTEDILTGFKMHARGWRSVYCMPKRPAFKGSAPINLSDRLNQVLRWALGSVEILFS-RH
OsCESA8 777 SVTEDILTGFKMHARGWRSIYCMPKRPAFKGSAPINLSDRLNQVLRWALGSVEILFS-RH
ZmCESA4 773 SVTEDILTGFKMHARGWRSIYCMPKRPAFKGSAPINLSDRLNQVLRWALGSVEILFS-RH
ZmCESA9 775 SVTEDILTGFKMHARGWRSIYCMPKRPAFKGSAPINLSDRLNQVLRWALGSVEILFS-RH
OsCESA10 ------------------------------------------------------------
OsCESA11 604 SPVWAG---RRLGLLQRLGYVARASYPLASLPLTVYCALPAVCLLTGKSTFPSD-VSYYD
ZmCESA10 832 CPLRYAYG-GRLKWLERFAYTNTIVYPFTSIPLLAYCTIPAVCLLTGKFIIPTL--NNLA
OsCESA7 817 CPLWYAYG-GRLKWLERFAYTNTIVYPFTSIPLLAYCTIPAVCLLTGKFIIPTL--NNLA
TaCESA4D 236 -----------------FAYTNTIVYPFTSIPLIAYCTIPAVCLLTGKFIIPTL--NNLA
TaCESA4B 632 CPLWYAYG-GRLKWLERFAYTNTIVYPFTSIPLIAYCTIPAVCLLTGKFIIPTL--NNLA
TaCESA4A 798 CPLWYAYG-GRLKWLERFAYTNTIVYPFTSIPLIAYCTIPAVCLLTGKFIIPTL--NNLA
HvCESA4 798 CPLWYAYG-GRLKWLERFAYTNTIVYPFTSIPLIAYCTIPAVCLLTGKFIIPTL--NNLA
ZmCESA11 737 CPLWYGYGGGRLKWLQRLSYINTIVYPFTSLPLVAYCCLPAICLLTGKFIIPTL--SNAA
OsCESA4 743 CPLWYGYGGGRLKWLQRLSYINTIVYPFTSLPLIAYCCLPAICLLTGKFIIPTL--SNAA
HvCESA7/5 738 CPLWYGYGGGRLRWLQRLSYINTIVYPFTSVPLVAYCCLPAICLLTGKFIIPIL--SNAA
TaCESA7B 737 CPLWYGYGGGRLRWLQRLSYINTIVYPFTSVPLVAYCCLPAICLLTGKFIIPIL--SNAA
TaCESA7D 653 CPLWYGYGGGRLRWLQRLSYINTIVYPFTSVPLVAYCCLPAICLLTGKFIIPIL--SNAA
TaCESA8D 659 SPLLYGYKGGNLKWLERFAYINTTIYPFTSLPLLAYCTLPAVCLLTGKFIMPPI--STFA
TaCESA8B 806 SPLLYGYKGGNLKWLERFAYINTTIYPFTSLPLLAYCTLPAVCLLTGKFIMPPVSISTFA
HvCESA8 808 SPLLYGYKGGNLKWLERFAYINTTIYPFTSLPLLAYCTLPAVCLLTGKFIMPPI--STFA
OsCESA9 808 SPLLYGYKNGNLKWLERFSYINTTIYPFTSLPLLAYCTLPAVCLLTGKFIMPPI--STFA
ZmCESA12 805 SPLLYGYKNGNLKWLERFAYINTTIYPFTSLPLLAYCTLPAVCLLTGKFIMPSI--STFA
ZmCESA13 805 SPLLYGYKNGNLKWLERFAYINTTIYPFTSLPLLAYCTLPAVCLLTGKFIMPSI--STFA
OsCESA6 847 CPLWYGYG-GGLKFLERFSYINSIVYPWTSIPLLAYCTLPAICLLTGKFITPEL--TNVA
ZmCESA8 849 CPLWYGYG-GGLKFLERFSYINSIVYPWTSIPLLAYCTLPAICLLTGKFITPEL--TNVA
TaCESA2A 846 CPLWYGYG-GGLKFLERFSYINSIVYPWTSIPLLAYCTLPAICLLTGKFITPEL--SNLA
TaCESA2B 846 CPLWYGYG-GGLKFLERFSYINSIVYPWTSIPLLAYCTLPAICLLTGKFITPEL--SNLA
TaCESA2D 846 CPLWYGYG-GGLKFLERFSYINSIVYPWTSIPLLAYCTLPAICLLTGKFITPEL--SNLA
HvCESA2 846 CPLWYGYG-GGLKFLERFSYINSIVYPWTSIPLLAYCTLPAICLLTGKFITPEL--SNLA
ZmCESA7 841 CPLWYGYG-GGLKFLERFSYINSIVYPWTSIPLLAYCTLPAICLLTGKFITPEL--NNVA
ZmCESA6 814 CPLWYGYG-GGLKFLERFSYINSIVYPWTSIPLLAYCTLPAICLLTGKFITPEL--NNVA
OsCESA3 848 CPLWYGYG-GGLKCLERFSYINSIVYPFTSIPLLAYCTLPAICLLTGKFITPEL--TNVA
OsCESA5 847 CPLWYGYG-GGLKCLERFSYINSIVYPWTSIPLLAYCTLPAICLLTGKFITPEL--TNIA
TaCESA9B 435 CPIWYNYG-GRLKLLERVAYINTIVYPLTSLPLIAYCVLPAICLLTNKFIIPEI--SNYA
ZmCESA3 828 CPIWYGYN-GRLKLLERLAYINTIVYPITSIPLVAYCVLPAICLLTNKFIIPAI--SNYA
ZmCESA1 829 CPIWYGYN-GRLKLLERLAYINTIVYPITSIPLIAYCVLPAICLLTNKFIIPEI--SNYA
ZmCESA2 828 CPIWYGYN-GRLKLLERLAYINTIVYPITSVPLIAYCVLPAICLLTNKFIIPEI--SNYA
OsCESA1 830 CPIWYGYN-GRLKLLERLAYINTIVYPITSIPLIAYCVLPAICLLTNKFIIPEI--SNYA
TaCESA5A 833 CPIWYNYG-GRLKLLERVAYINTIVYPITSLPLIAYCVLPAICLLTNKFIIPEI--SNYA
HvCESA6 828 CPIWYNYG-GRLKLLERVAYINTIVYPITSLPLIAYCVLPAICLLTNKFIIPEI--SNYA
TaCESA6B 823 CPIWYNYG-GRLKLLERVAYINTIVYPLTSLPLIAYCVLPAICLLTNKFIIPEI--SNYA
TaCESA6A 829 CPIWYNYG-GRLKLLERVAYINTIVYPLTSLPLIAYCVLPAICLLTNKFIIPEI--SNYA
TaCESA5D 833 CPIWYNYG-GRLKLLERVAYINTIVYPITSLPLIAYCVLPAICLLTNKFIIPEI--SNYA
TaCESA5B 832 CPIWYNYG-GRLKLLERVAYINTIVYPITSLPLIAYCVLPAICLLTNKFIIPEI--SNYA
HvCesA9 435 CPIWYNYG-GRLKLLERVAYINTIVYPITSLPLIAYCVLPAICLLTNKFIIPEI--SNYA
ZmCESA5 831 CPIWYGYG-GRLKFLERFAYINTTIYPLTSIPLLLYCILPAVCLLTGKFIIPKI--SNLE
OsCESA2 828 CPIWYGYG-GRLKFLERFAYINTTIYPLTSIPLLLYCILPAICLLTGKFIIPEI--SNFA
HvCESA3 823 CPIWYGYG-GRLKFLERFAYINTTIYPLTSIPLLIYCILPAVCLLTGKFIIPQI--SNIA
TaCESA3B 841 CPIWYGYG-GRLKFLERFAYINTTIYPLTSIPLLIYCILPAVCLLTGKFIIPQI--SNIA
TaCESA3D 841 CPIWYGYG-GRLKFLERFAYINTTIYPLTSIPLLIYCILPAVCLLTGKFIIPQI--SNIA
TaCESA3A 662 CPIWYGYG-GRLKFLERFAYINTTIYPLTSIPLLIYCILPAVCLLTGKFIIPQI--SNIA
HvCESA1 835 CPLWYGYG-GRLKFLERFAYINTTIYPLTSLPLLVYCILPAICLLTGKFIMPEI--SNLA
TaCESA1D 767 CPLWYGYG-GRLKFLERFAYINTTIYPLTSLPLLVYCILPAICLLTGKFIMPEI--SNLA
TaCESA1A 835 CPLWYGYG-GRLKFLERFAYINTTIYPLTSLPLLVYCILPAICLLTGKFIMPEI--SNLA
TaCESA1B 835 CPLWYGYG-GRLKFLERFAYINTTIYPLTSLPLLVYCILPAICLLTGKFIMPEI--SNLA
OsCESA8 836 CPIWYGYG-GRLKFLERFAYINTTIYPLTSIPLLIYCVLPAICLLTGKFIIPEI--SNFA
ZmCESA4 832 CPLWYGYG-GRLKFLERFAYINTTIYPLTSIPLLIYCILPAICLLTGKFIIPEI--SNFA
ZmCESA9 834 CPLWYGYG-GRLKFLERFAYINTTIYPLTSLPLLIYCILPAICLLTGKFIIPEI--SNFA
OsCESA10 ------------------------------------------------------------
OsCESA11 660 GVLLILLLFSVAASVALELRWSRVPLRAWWRDEKLWMVTATSASLAAVFQGILSACTGID
ZmCESA10 889 SIWFIALFLSIIATSVLELRWSGVSIEDWWRNEQFWVIGGVSAHLFAVFQGFLKVLGGVD
OsCESA7 874 SIWFIALFLSIIATGVLELRWSGVSIEDWWRNEQFWVIGGVSAHLFAVFQGLLKVLGGVD
TaCESA4D 277 SIWFIALFMSIIATGVLELRWSGVSIEDWWRNEQFWVIGGVSAHLFAVF-----------
TaCESA4B 689 SIWFIALFMSIIATGVLELRWSGVSIEDWWRNEQFWVIGGVSAHLFAVFQGFLKVLGGVD
TaCESA4A 855 SIWFIALFMSIIATGVLELRWSGVSIEDWWRNEQFWVIGGVSAHLFAVFQGFLKVLGGVD
HvCESA4 855 SIWFIALFMSIIATGVLELRWSGVSIEDWWRNEQFWVIGGVSAHLFAVFQGFLKVLGGVD
ZmCESA11 795 TIWFLGLFMSIIVTSVLELRWSGIGIEDWWRNEQFWVIGGVSAHLFAVFQGILKMIAGLD
OsCESA4 801 TIWFLGLFISIIVTSVLELRWSGIGIEDWWRNEQFWVIGGVSAHLFAVFQGILKMIAGLD
HvCESA7/5 796 TIWFLGLFTSIILTSVLELRWSGIGIEDWWRNEQFWVIGGVSAHLFAVFQGILKMVIGLD
TaCESA7B 795 TIWFLGLFTSIILTSVLELRWSGIGIEDWWRNEQFWVIGGVSAHLFAVFQGILKMVIGLD
TaCESA7D 711 TIWFLGLFTSIILTSVLELRWSGIGIEDWWRNEQFWVIGGVSAHLFAVFQGILKMVIGLD
TaCESA8D 717 SLFFISLFISIFATGILELRWSGVSIEEWWRNEQFWVIGGVSAHLFAVIQGLLKVLAGID
TaCESA8B 866 SLFFISLFISIFATGILELRWSGVSIEEWWRNEQFWVIGGVSAHLFAVIQGLLKVLAGID
HvCESA8 866 SLFFISLFISIFATGILELRWSGVSIEEWWRNEQFWVIGGVSAHLFAVIQGLLKVLAGID
OsCESA9 866 SLFFIALFISIFATGILEMRWSGVSIEEWWRNEQFWVIGGVSAHLFAVVQGLLKVLAGID
ZmCESA12 863 SLFFIALFMSIFATGILEMRWSGVSIEEWWRNEQFWVIGGVSAHLFAVVQGLLKVLAGID
ZmCESA13 863 SLFFIALFMSIFATGILEMRWSGVSIEEWWRNEQFWVIGGVSAHLFAVVQGLLKVLAGID
OsCESA6 904 SLWFMSLFICIFVTGILEMRWSGVAIDDWWRNEQFWVIGGVSSHLFAVFQGLLKVLAGVD
ZmCESA8 906 SIWFMALFICISVTGILEMRWSGVAIDDWWRNEQFWVIGGVSAHLFAVFQGLLKVFAGID
TaCESA2A 903 SIWYMSLFICIFATGILEMRWARVAVDDWWRNEQFWVIGGVSAHLFAVFQGLLKVIAGVD
TaCESA2B 903 SIWYMSLFICIFATGILEMRWARVAVDDWWRNEQFWVIGGVSAHLFAVFQGLLKVIAGVD
TaCESA2D 903 SIWYMSLFICIFATGILEMRWARVAVDDWWRNEQFWVIGGVSAHLFAVFQGLLKVIAGVD
HvCESA2 903 SIWYMSLFICIFATGILEMRWARVAVDDWWRNEQFWVIGGVSAHLFAVFQGLLKVIAGVD
ZmCESA7 898 SLWFMSLFICIFATSILEMRWSGVGIDDWWRNEQFWVIGGVSSHLFAVFQGLLKVIAGVD
ZmCESA6 871 SLWFMSLFICIFATSILEMRWSGVGIDDWWRNEQFWVIGGVSSHLFAVFQGLLKVIAGVD
OsCESA3 905 SLWFMSLFICIFATGILEMRWSGVGIDDWWRNEQFWVIGGVSSHLFALFQGLLKVIAGID
OsCESA5 904 SLWFMSLFICIFATGILEMRWSGVGIDDWWRNEQFWVIGGVSSHLFAVFQGLLKVIAGID
TaCESA9B 492 GMFFILMFASIFATGILELRWSGVGIEDWWRNEQFWVIGGTXXXXXXXXXXXXX--XXXX
ZmCESA3 885 GAFFILLFASIFATGILELRWSGVGIEDWWRNEQFWVIGGTSAHLFAVFQGLLKVLAGID
ZmCESA1 886 GMFFILLFASIFATGILELRWSGVGIEDWWRNEQFWVIGGTSAHLFAVFQGLLKVLAGID
ZmCESA2 885 GMFFILLFASIFATGILELRWSGVGIEDWWRNEQFWVIGGTSAHLFAVFQGLLKVLAGID
OsCESA1 887 GMFFILLFASIFATGILELRWSGVGIEDWWRNEQFWVIGGTSAHLFAVFQGLLKVLAGID
TaCESA5A 890 GTFFILMFASIFATGILELRWSGVGIEDWWRNEQFWVIGGTSAHLFAVFQGLLKVLAGID
HvCESA6 885 GMFFILMFASIFATGILELRWSGVGIEDWWRNEQFWVIGGTSAHLFAVFQGLLKVLAGID
TaCESA6B 880 GMFFILMFASIFATGILELRWSGVGIEDWWRNEQFWVIGGTSAHLFAVFQGLLKVLAGID
TaCESA6A 886 GMFFILMFASIFATGILELRWSGVGIEDWWRNEQFWVIGGTSAHLFAVFQGLLKVLAGID
TaCESA5D 890 GMFFILMFASIFATGILELRWSGVGIEDWWRNEQFWVIGGTSAHLFAVFQGLLKVLAGID
TaCESA5B 889 GMFFILMFASIFATGILELRWSGVGIEDWWRNEQFWVIGGTSAHLFAVFQGLLKVLAGID
HvCesA9 492 GMFFILMFASIFATGILELRWSGVGIEDWWRNEQFWVIGGTSAHLFAVFQGLLKVLAGID
ZmCESA5 888 SVWFISLFISIFATGILEMRWSGVGIDEWWRNEQFWVIGGISAHLFAVFQGLLKVLAGID
OsCESA2 885 SIWFISLFLSIFATGILEMRWSGVGIDEWWRNEQFWVIGGISAHLFAVFQGLLKVLAGID
HvCESA3 880 SIWFISLFISIFATGILEMRWSGVGIDEWWRNEQFWVIGGISAHLFAVFQGLLKVLAGID
TaCESA3B 898 SIWFISLFISIFATGILEMRWSGVGIDEWWRNEQFWVIGGISAHLFAVFQGLLKVLAGID
TaCESA3D 898 SIWFISLFISIFATGILEMRWSGVGIDEWWRNEQFWVIGGISAHLFAVFQGLLKVLAGID
TaCESA3A 719 SIWFISLFISIFATGILEMRWSGVGIDEWWRNEQFWVIGGISAHLFAVFQGLLKVLAGID
HvCESA1 892 SIWFIALFLSIFATGILEMRWSGVGIDEWWRNEQFWVIGGISAHLFAVFQGLLKVLAGID
TaCESA1D 824 SIWFIALFLSIFATGILEMRWSGVGIDEWWRNEQFWVIGGISAHLFAVFQGLLKVLAGID
TaCESA1A 892 SIWFIALFLSIFATGILEMRWSGVGIDEWWRNEQFWVIGGISAHLFAVFQGLLKVLAGID
TaCESA1B 892 SIWFIALFLSIFATGILEMRWSGVGIDEWWRNEQFWVIGGISAHLFAVFQGLLKVLAGID
OsCESA8 893 SIWFISLFISIFATGILEMRWSGVGIDEWWRNEQFWVIGGISAHLFAVFQGLLKVLAGID
ZmCESA4 889 SIWFISLFISIFATGILEMRWSGVGIDEWWRNEQFWVIGGISAHLFAVFQGLLKVLAGID
ZmCESA9 891 SIWFISLFISIFATGILEMRWSGVGIDEWWRNEQFWVIGGISAHLFAVFQGLLKVLAGID
OsCESA10 ------------------------------------------------------------
OsCESA11 720 VAFSTETAASPPKRPAAGNDDGEEEAALASEITMRWTNLLVAPTSVVVANLAGVVAAVAY
ZmCESA10 949 TSFTVTSKA-----------AGDEADAFGDLYLFKWTTLLVPPTTLIIINMVGIVAGVSD
OsCESA7 934 TNFTVTSKA-----------AADETDAFGELYLFKWTTLLVPPTTLIIINMVGIVAGVSD
TaCESA4D 326 ------------------------------------------------------------
TaCESA4B 749 TNFTVTSKA-----------GADEADAFGDLYLFKWTTLLIPPTTLIIINMVGIVAGVSD
TaCESA4A 915 TNFTVTSKA-----------GADEADAFGDLYLFKWTTLLIPPTTLIIINMVGIVAGVSD
HvCESA4 915 TNFTVTSKA-----------GADEADAFGDLYLFKWTTLLIPPTTLIIINMVGIVAGVSD
ZmCESA11 855 TNFTVTAKA-----------T--DDTEFGELYLFKWTTVLIPPTSILVLNLVGVVAGFSA
OsCESA4 861 TNFTVTAKA-----------T--DDTEFGELYVFKWTTVLIPPTSILVLNLVGVVAGFSD
HvCESA7/5 856 TNFTVTSKA-----------A--EDGDFAELYVFKWTTVLIPPTTILVLNLVGVVAGFSD
TaCESA7B 855 TNFTVTSKA-----------A--EDGDFAELYVFKWTTVLIPPTTILVLNLVGVVAGFSD
TaCESA7D 771 TNFTVTSKA-----------A--EDGDFAELYVFKWTTVLIPP------------AGFSD
TaCESA8D 777 TNFTVTSKA-----------TGDEDDEFAELYAFKWTTLLIPPTTLLVINIIGVVAGISD
TaCESA8B 926 TNFTVTSKA-----------TGDEDDEFAELYAFKWTTLLIPPTTLLVINIIGVVAGISD
HvCESA8 926 TNFTVTSKA-----------TGDEDDEFAELYAFKWTTLLIPPTTLLVINIIGVVAGISD
OsCESA9 926 TNFTVTSKA-----------TGDEDDEFAELYAFKWTTLLIPPTTLLILNIIGVVAGVSD
ZmCESA12 923 TNFTVTSKA-----------TGDEDDEFAELYAFKWTTLLIPPTTLLIINVIGVVAGISD
ZmCESA13 923 TNFTVTSKA-----------TGDEDDEFAELYAFKWTTLLIPPTTLLIINIIGVVAGISD
OsCESA6 964 TSFTVTSKA------------GD-DEEFSELYTFKWTTLLIPPTTLLLLNFIGVVAGVSN
ZmCESA8 966 TSFTVTSKA------------GD-DEEFSELYTFKWTTLLIPPTTLLLLNFIGVVAGISN
TaCESA2A 963 TSFTVTTKA------------GD-DEEFSELYTFKWTTLLIPPTTLLLLNFIGVVAGISN
TaCESA2B 963 TSFTVTTKA------------GD-DEEFSELYTFKWTTLLIPPTTLLLLNFIGVVAGISN
TaCESA2D 963 TSFTVTTKA------------GD-DEEFSELYTFKWTTLLIPPTTLLLLNFIGVVAGISN
HvCESA2 963 TSFTVTTKA------------GD-DEEFSELYTFKWTTLLIPPTTLLLLNFIGVVAGISN
ZmCESA7 958 TSFTVTSKG------------GD-DEEFSELYTFKWTTLLIPPTTLLLLNFIGVVAGVSN
ZmCESA6 931 TSFTVTSKG------------GD-DEEFSELYTFKWTTLLIPPTTLLLLNFIGVVAGISN
OsCESA3 965 TSFTVTSKG------------GD-DEEFSELYTFKWTTLLIPPTTLLLLNFIGVVAGVSN
OsCESA5 964 TSFTVTSKG------------GD-DEEFSELYTFKWTTLLIPPTTLLLLNFIGVVAGVSN
TaCESA9B 550 XXXXXXXXX------------NDEDGDFAELYVFKWTSLLIPPTTVLVINLVGMVAGISY
ZmCESA3 945 TNFTVTSKA------------TDDDGDFAELYVFKWTTLLIPPTTVLVINLVGIVAGVSY
ZmCESA1 946 TNFTVTSKA------------SDEDGDFAELYVFKWTSLLIPPTTVLVINLVGMVAGISY
ZmCESA2 945 TNFTVTSKA------------SDEDGDFAELYVFKWTSLLIPPTTVLVINLVGMVAGISY
OsCESA1 947 TNFTVTSKA------------SDEDGDFAELYVFKWTSLLIPPTTVLVINLVGMVAGISY
TaCESA5A 950 TNFTVTSKA------------NDEDGDFAELYVFKWTSLLIPPTTVLVINLVGMVAGISY
HvCESA6 945 TNFTVTSKA------------NDEDGDFAELYVFKWTSLLIPPTTVLVINLVGMVAGISY
TaCESA6B 940 TNFTVTSKA------------NDEDGDFAELYVFKWTSLLIPPTTVLVINLVGMVAGISY
TaCESA6A 946 TNFTVTSKA------------NDEDGDFAELYVFKWTSLLIPPTTVLVINLVGMVAGISY
TaCESA5D 950 TNFTVTSKA------------NDEDGDFAELYVFKWTSLLIPPTTVLVINLVGMVAGISY
TaCESA5B 949 TNFTVTSKA------------NDEDGDFAELYVFKWTSLLIPPTTVLVINLVGMVAGISY
HvCesA9 552 TNFTVTSKA------------NDEDGDFAELYVFKWTSLLIPPTTVLVINLVGMVAGISY
ZmCESA5 948 TSFTVTSKA------------TDEEGDFAELYMFKWTTLLIPPTTILIINLVGVVAGISY
OsCESA2 945 TSFTVTSKA------------SDEEGDFAELYMFKWTTLLIPPTTILIINLVGVVAGISY
HvCESA3 940 TSFTVTSKA------------SDEDNDFAELYMFKWTTLLIPPTTILIINLVGVVAGTSY
TaCESA3B 958 TSFTVTSKA------------SDEDNDFAELYMFKWTTLLIPPTTILIINLVGVVAGTSY
TaCESA3D 958 TSFTVTSKA------------SDEDNDFAELYMFKWTTLLIPPTTILIINLVGVVAGTSY
TaCESA3A 779 TSFTVTSKA------------SDEDNDFAELYMFKWTTLLIPPTTILIINLVGVVAGTSY
HvCESA1 952 TNFTVTSKA------------NDEEGDFAELYMFKRTTLLIPPTTILIINMVGVVAGTSY
TaCESA1D 884 TNFTVTSKA------------NDEEGDFAELYMFKWTTLLIPPTTILIINMVGVVAGTSY
TaCESA1A 952 TNFTVTSKA------------NDEEGDFAELYMFKWTTLLIPPTTILIINMVGVVAGTSY
TaCESA1B 952 TNFTVTSKA------------NDEEGDFAELYMFKWTTLLIPPTTILIINMVGVVAGTSY
OsCESA8 953 TNFTVTSKA------------SDEDGDFAELYMFKWTTLLIPPTTILIINLVGVVAGISY
ZmCESA4 949 TNFTVTSKA------------SDEDGDFAELYMFKWTTLLIPPTTILIINLVGVVAGISY
ZmCESA9 951 TNFTVTSKA------------SDEDGDFAELYMFKWTTLLIPPTTILIINLVGVVAGISY
OsCESA10 ------------------------------------------------------------
OsCESA11 780 GVDHGYYQSWGALGAKLALAGWVVAHLQGFLRGLLAPRDRAPPTIAVLWSVVFVSVASLL
ZmCESA10 998 AVNNG-YGSWGPLFGKLFFSFWVIVHLYPFLKGLMGRQNR-TPTIVVLWSILLASIFSLV
OsCESA7 983 AVNNG-YGSWGPLFGKLFFSFWVILHLYPFLKGLMGRQNR-TPTIVVLWSILLASIFSLV
TaCESA4D 326 ---NG-YGSWGPLFGKLFFSFWVIVHLYPFLKGLMGRQNR-TPTIVVLWSVLLASIFSLV
TaCESA4B 798 AVNNG-YGSWGPLFGKLFFSFWVIVHLYPFLKGLMGRQNR-TPTIVVLWSVLLASIFSLV
TaCESA4A 964 AVNNG-YGSWGPLFGKLFFSFWVIVHLYPFLKGLMGRQNR-TPTIVVLWSVLLASIFSLV
HvCESA4 964 AVNNG-YGSWGPLFGKLFFSFWVIVHLYPFLKGLMGRQNR-TPTIVVLWSVLLASIFSLV
ZmCESA11 902 ALNSG-YESWGPLFGKVFFAMWVIMHLYPFLKGLMGRQNR-TPTIVVLWSVLLASVFSLL
OsCESA4 908 ALNSG-YESWGPLFGKVFFAMWVIMHLYPFLKGLMGRQNR-TPTIVVLWSVLLASVFSLL
HvCESA7/5 903 ALNSG-YESWGPLFGKVFFSMWVIMHLYPFLKGLMGRQNR-TPTIVILWSVLLASVFSLL
TaCESA7B 902 ALNSG-YESWGPLFGKVFFAMWVIMHLYPFLKGLMGRQNR-TPTIVILWSVLLASVFSLL
TaCESA7D 806 ALNSG-YESWGPLFGKVFFAMWVIMHLYPFLKGLMGRQNR-TPTIVILWSVLLASVFSLL
TaCESA8D 826 AINNG-YQSWGPLFGKLFFAFWVIVHLYPFLKGLMGRQNR-TPTIVIIWSVLLASIFSLL
TaCESA8B 975 AINNG-YQSWGPLFGKLFFAFWVIVHLYPFLKGLMGRQNR-TPTIVIIWSVLLASIFSLL
HvCESA8 975 AINNG-YQSWGPLFGKLFFAFWVIVHLYPFLKGLMGRQNR-TPTIVIIWSVLLASIFSLL
OsCESA9 975 AINNG-SEAWGPLFGKLFFAFWVIVHLYPFLKGLMGRQNR-TPTIVVIWSVLLASIFSLL
ZmCESA12 972 AINNG-YQSWGPLFGKLFFAFWVIVHLYPFLKGLMGRQNR-TPTVVVIWSILLASIFSLL
ZmCESA13 972 AINNG-YQSWGPLFGKLFFAFWVIVHLYPFLKGLMGRQNR-TPTIVVIWSVLLASIFSLL
OsCESA6 1011 AINNG-YESWGPLFGKLFFAFWVIVHLYPFLKGLVGRQNR-TPTIVIVWSILLASIFSLL
ZmCESA8 1013 AINNG-YESWGPLFGKLFFAFWVIVHLYPFLKGLVGRQNR-TPTIVIVWSILLASIFSLL
TaCESA2A 1010 AINNG-YESWGPLFGKLFFAFWVIVHLYPFLKGLLGRQNR-TPTIVIVWSILLASIISLL
TaCESA2B 1010 AINNG-YESWGPLFGKLFFAFWVIVHLYPFLKGLLGRQNR-TPTIVIVWSILLASIISLL
TaCESA2D 1010 AINNG-YESWGPLFGKLFFAFWVIVHLYPFLKGLLGRQNR-TPTIVIVWSILLASIISLL
HvCESA2 1010 AINNG-YESWGPLFGKLFFAFWVIVHLYPFLKGLLGRQNR-TPTIVIVWSILLASIISLL
ZmCESA7 1005 AINNG-YESWGPLFGKLFFAFWVIVHLYPFLKGLVGRQNR-TPTIVIVWSILLASIFSLL
ZmCESA6 978 AINNG-YESWGPLFGKLFFAFWVIVHLYPFLKGLVGRQNR-TPTIVIVWSILLASIFSLL
OsCESA3 1012 AINNG-YESWGPLFGKLFFAFWVIVHLYPFLKGLVGRQNR-TPTIVIVWSILLASIFSLL
OsCESA5 1011 AINNG-YESWGPLFGKLFFAFWVIVHLYPFLKGLVGRQNR-TPTIVIVWSILLASIFSLL
TaCESA9B 598 AINSG-YQSWGPLFGKLFFSIWVILHLYPFLKGLMGKQNR-TPTIVIVWSILLASIFSLL
ZmCESA3 993 AINSG-YQSWGPLFGKLFFAIWVILHLYPFLKGLMGKQNR-TPTIVIVWSVLLASIFSLL
ZmCESA1 994 AINSG-YQSWGPLFGKLFFSIWVILHLYPFLKGLMGRQNR-TPTIVIVWSILLASIFSLL
ZmCESA2 993 AINSG-YQSWGPLFGKLFFSIWVILHLYPFLKGLMGRQNR-TPTIVIVWSILLASIFSLL
OsCESA1 995 AINSG-YQSWGPLFGKLFFSIWVILHLYPFLKGLMGRQNR-TPTIVIVWSILLASIFSLL
TaCESA5A 998 AINSG-YQSWGPLFGKLFFSIWVILHLYPFLKGLMGKQNR-TPTIVIVWSILLASIFSLL
HvCESA6 993 AINSG-YQSWGPLFGKLFFSIWVILHLYPFLKGLMGKQNR-TPTIVIVWSILLASIFSLL
TaCESA6B 988 AINSG-YQSWGPLFGKLFFSIWVILHLYPFLKGLMGKQNR-TPTIVIVWSILLASIFSLL
TaCESA6A 994 AINSG-YQSWGPLFGKLFFSIWVILHLYPFLKGLMGKQNR-TPTIVIVWSILLASIFSLL
TaCESA5D 998 AINSG-YQSWGPLFGKLFFSIWVILHLYPFLKGLMGKQNR-TPTIVIVWSILLASIFSLL
TaCESA5B 997 AINSG-YQSWGPLFGKLFFSIWVILHLYPFLKGLMGKQNR-TPTIVIVWSILLASIFSLL
HvCesA9 600 AINSG-YQSWGPLFGKLFFSIWVILHLYPFLKGLMGKQNR-TPTIVIVWSILLASIFSLL
ZmCESA5 996 AINSG-YQSWGPLFGKLFFAFWVIVHLYPFLKGLMGKQNR-TPTIVVVWAILLASIFSLM
OsCESA2 993 AINSG-YQSWGPLFGKLFFAFWVIVHLYPFLKGLMGRQNR-TPTIVVVWAILLASIFSLL
HvCESA3 988 AINSG-YQSWGPLFGKLFFAFWVIIHLYPFLKGLMGRQNR-TPTIVVVWAILLASIFSLL
TaCESA3B 1006 AINSG-YQSWGPLFGKLFFAFWVIIHLYPFLKGLMGRQNR-TPTIVVVWAILLASIFSLL
TaCESA3D 1006 AINSG-YQSWGPLFGKLFFAFWVIIHLYPFLKGLMGRQNR-TPTIVVVWAILLASIFSLL
TaCESA3A 827 AINSG-YQSWGPLFGKLFFAFWVIIHLYPFLKGLMGRQNR-TPTIVVVWAILLASIFSLL
HvCESA1 1000 AINSG-YQSWGPLFGKLFFAFWVIVHLYPFLKGLMGRQNR-TPTIVIVWAVLLASIFSLL
TaCESA1D 932 AINSG-YQSWGPLFGKLFFAFWVIVHLYPFLKGLMGRQNR-TPTIVIVWAVLLASIFSLL
TaCESA1A 1000 AINSG-YQSWGPLFGKLFFAFWVIVHLYPFLKGLMGRQNR-TPTIVIVWAVLLASIFSLL
TaCESA1B 1000 AINSG-YQSWGPLFGKLFFAFWVIVHLYPFLKGLMGRQNR-TPTIVIVWAVLLASIFSLL
OsCESA8 1001 AINSG-YQSWGPLFGKLFFAFWVIVHLYPFLKGLMGRQNR-TPTIVVVWAILLASIFSLL
ZmCESA4 997 AINSG-YQSWGPLFGKLFFAFWVIVHLYPFLKGLMGRQNR-TPTIVVVWAILLASIFSLL
ZmCESA9 999 AINSG-YQSWGPLFGKLFFAFWVIVHLYPFLKGLMGRQNR-TPTIVVVWAILLASIFSLL
OsCESA10 -------------------------------------------------
OsCESA11 840 WVHAASFSAPTAA-PTTEQPI-L--------------------------
ZmCESA10 1056 WVRIDPFIPKAK-GPILKPCG-VEC------------------------
OsCESA7 1041 WVRIDPFIPKPK-GPVLKPCG-VSC------------------------
TaCESA4D 381 WVRIDPFIAKPK-GPIL--------------------------------
TaCESA4B 856 WVRIDPFIAKPK-GPILKPCG-VQC------------------------
TaCESA4A 1022 WVRIDPFIAKPK-GPILKPCG-VQC------------------------
HvCESA4 1022 WVRIDPFIAKPK-GPILKPCG-VQC------------------------
ZmCESA11 960 WVKIDPFVGGTET-VNTNNCNTHLLIHHRSAAVVPRRTCFWCCKRGLPA
OsCESA4 966 WVKIDPFIGSSET-TTTNSCANFDC------------------------
HvCESA7/5 961 WVKIDPFISGAET-VATGACSSIDC------------------------
TaCESA7B 960 WVKIDPFITGAET-VATGACSSIDC------------------------
TaCESA7D 864 WVKIDPFITGAET-VATGACSSIDC------------------------
TaCESA8D 884 WVRIDPFTVKAK-GPDVKQCG-INC------------------------
TaCESA8B 1033 WVRIDPFTVKAK-GPDVKQCG-INC------------------------
HvCESA8 1033 WVRIDPFTVKAK-GPDVKQCG-INC------------------------
OsCESA9 1033 WVRIDPFTIKAR-GPDVRQCG-INC------------------------
ZmCESA12 1030 WVRIDPFIVRTK-GPDVRQCG-INC------------------------
ZmCESA13 1030 WVRIDPFIVRTK-GPDVRQCG-INC------------------------
OsCESA6 1069 WVRIDPFLAKNN-GPLLEECG-LDCN-----------------------
ZmCESA8 1071 WVRVDPFLAKSN-GPLLEECG-LDCN-----------------------
TaCESA2A 1068 WVRVNPFLAKTD-GPLLEECG-LDCT-----------------------
TaCESA2B 1068 WVRVNPFLAKTD-GPLLEECG-LDCT-----------------------
TaCESA2D 1068 WVRVNPFLAKTD-GPLLEECG-LDCT-----------------------
HvCESA2 1068 WVRVNPFLAKTD-GPLLEECG-LDCT-----------------------
ZmCESA7 1063 WVRIDPFLAKDD-GPLLEECG-LDCN-----------------------
ZmCESA6 1036 WVRIDPFLAKDD-GPLLEECG-LDCN-----------------------
OsCESA3 1070 WVRIDPFLAKND-GPLLEECG-LDCN-----------------------
OsCESA5 1069 WVRIDPFLAKND-GPLLEECG-LDCN-----------------------
TaCESA9B 656 WVKIDPFISDTQKAVAMGQCG-VNC------------------------
ZmCESA3 1051 WVKIDPFISPTQKALSRGQCG-VNC------------------------
ZmCESA1 1052 WVKIDPFISPTQKAAALGQCG-VNC------------------------
ZmCESA2 1051 WVKIDPFISPTQKAAALGQCG-VNC------------------------
OsCESA1 1053 WVKIDPFISPTQKAVALGQCG-VNC------------------------
TaCESA5A 1056 WVKIDPFISDTQKAVAMGQCG-VNC------------------------
HvCESA6 1051 WVKIDPFISDTQKAVAMGQCG-VNC------------------------
TaCESA6B 1046 WVKIDPFISDTQKAVAMGQCG-VNC------------------------
TaCESA6A 1052 WVKIDPFISDTQKAVAMGQCG-VNC------------------------
TaCESA5D 1056 WVKIDPFISDTQKAVAMGQCG-VNC------------------------
TaCESA5B 1055 WVKIDPFISDTQKAVAMGQCG-VNC------------------------
HvCesA9 658 WVKIDPFISDTQKAVAMGQCG-VNC------------------------
ZmCESA5 1054 WVRIDPFTTRVT-GPDIAKCG-INC------------------------
OsCESA2 1051 WVRIDPFTTRVT-GPDTQKCG-INC------------------------
HvCESA3 1046 WVRIDPFTTRVT-GPDIQMCG-INC------------------------
TaCESA3B 1064 WVRIDPFTTRVT-GPDIQMCG-INC------------------------
TaCESA3D 1064 WVRIDPFTTRVT-GPDIRMCG-INC------------------------
TaCESA3A 885 WVRIDPFTTRVT-GPDIQMCG-INC------------------------
HvCESA1 1058 WVCVDPFTTRLA-GPNIQTCG-INC------------------------
TaCESA1D 990 WVRVDPFTTRLA-GPNIQTCG-INC------------------------
TaCESA1A 1058 WVRVDPFTTRLA-GPNIQTCG-INC------------------------
TaCESA1B 1058 WVRVDPFTTRLA-GPNIQTCG-INC------------------------
OsCESA8 1059 WVRIDPFTTRVT-GPDTQTCG-INC------------------------
ZmCESA4 1055 WVRIDPFTTRVT-GPDTQTCG-INC------------------------
ZmCESA9 1057 WVRIDPFTNRVT-GPDTRTCG-INC------------------------
